# RNA-sequencing (transcriptomic) data collected in liver and lung of male and female B6C3F1 mice exposed to various dose levels of 4-methylimidazole for 2, 5, or 28 days

**DOI:** 10.1016/j.dib.2021.107420

**Published:** 2021-09-23

**Authors:** Michael B. Black, Melvin E. Andersen, Salil N. Pendse, Susan J. Borghoff, Michael Streicker, Patrick D. McMullen

**Affiliations:** a ScitoVation LLC 100 Capitola Dr., Suite 106, Durham, NC 27713, USA; b ToxStrategies Inc. 1249 Kildaire Farm Rd. #134, Cary, NC 27511, USA; c Integrated Laboratory Systems (ILS) 601 Keystone Park Drive, Suite 200 Morrisville, NC 27560, USA

**Keywords:** 4-Methylimidazole, 4-MeI, Mouse lung and liver, RNA-Seq, Dose response

## Abstract

The National Toxicology Program (NTP) reported that chronic exposure to varying dietary concentrations of 4-methylimidazole (4-MeI) increased lung tumors in female and male mice [1]. In this study, mice (male and female B6C3F1 mice) were either administered 4-MeI by oral gavage (0, 50, 100, 200, or 300 mg/kg/day) for 2 days or exposed for 5 and 28 days to 4-MeI in the diet (0, 150, 300, 1250, or 2500 ppm) and whole transcriptome (RNA-Sequencing) data from 4-MeI-exposed B6C3F1 mice to determine whether changes occurred in the target (lung) and nontarget (liver) tissues. This analysis was conducted to provide information with which to evaluate biological processes affected by exposure to 4-MeI, with a focus on identifying key events that could be used to propose a plausible mode of action (MoA) for mouse lung tumors [2].

## Specifications Table

**Table:** 

Subject area	Biology
Specific subject area	Transcriptomic changes characteristic of carcinogenic activity in target (lung) vs. nontarget (liver) mouse tissues on exposure to test substance.
Type of data	Tables, graphs, figures
How data were acquired	*In vivo* lung & liver transcriptomics using Illumina NextSeq 500. In-life data; clinical observations, body weight, and food consumption.
Data format	Raw data: Transcriptomics; Illumina FASTQ files and individual animal body weights and food consumption. Analyzed data: Fig. 1, Fig. 2, Fig. 3 and Table 1, Table 2, Table 3.
Parameters for data collection	FASTQ files were mapped to iGenomes UCSC mm10 reference, reads per sample counted with HTSeq. Body weight, food consumption, and clinical observations are reported in the study report appendices (Supplemental Data File 1).
Description of data collection	Male and female mice exposed to 4-MeI for 2, 5, and 28 days at four dose levels, plus vehicle-only controls. Eight animals per sex were exposed at each dose level and time point, with the six samples per condition (sex, dose, and time), yielding the best total RNA used for whole-transcriptome sequencing. Samples were sequenced by 75 bp paired-end reads, with four bar-coded pairs of reads per sample (Supplemental Data Files 2, 3, and 4). Body-weight changes, food consumption, and clinical observations were recorded over the course of the study (Supplemental Data File 1).
Data source location	Integrated Laboratory Systems, Morrisville, NC, USA NCBI GEO, National Library of Medicine, Washington, DC, USA. In-life data as Supplemental Data File 1
Data accessibility	Repository name: NCBI GEO Data identification number: GEO Accession Number GSE129622 Direct link to data: [https://www.ncbi.nlm.nih.gov/geo/query/acc.cgi?acc=GSE129622]
Related research article	Susan J. Borghoff, Seneca E. Fitch, Michael B. Black, Patrick D. McMullen, Melvin E. Andersen, Grace A. Chappell. 2021. A systematic approach to evaluate plausible mode of actions for mouse lung tumors in mice exposed to 4-methylimidazole. Regulatory Toxicology and Pharmacology 124:104977 [10.1016/j.yrtph.2021.104977] published online.

## Value of the Data

• Gene expression data provide a powerful tool for identifying key molecular initiating and key events to inform a mode of action (MoA) for mouse lung tumors in mice exposed to high levels of 4-MeI [3], [4].
• Gene expression data provide the ability to generate cellular mechanistic hypotheses relative to cellular biology preceding apical adverse endpoints [5].
• These data are useful in considering plausible MoAs for mouse lung tumors that occur in mice with chronic dietary exposure to 4-MeI.

## 1 Data Description

Tables 1 and 2 provide summary data of in-life animal observations, including food consumption and body-weight changes with exposure to 4-MeI in male mice and female mice, respectively. Supplemental Data File 1 provides the final report from the animal study, with complete descriptions of the experimental design, animal model, dose levels, and endpoints collected over the 28-day exposure period, as outlined in Tables 4 and 5, below, in the Experimental Design, Materials, and Methods section.

**Table 1:** In-life animal observations: Mean feed consumption and body weight change in male mice exposed to 4-MeI (Supplemental Data File 1 provides complete raw data for both male and female mice).

Day of Termination	Dose Level	Mean Feed Consumption (g/kg body weight/day)^1^ ±SD	Mean 4-MeI Consumed (mg/kg body weight/day) ±SD	Initial Group Mean Body Weight (g) ±SD	Final Group Mean Body Weight (g) ±SD	Mean Body Weight Gain^2^ (g) ±SD
**Day 2 (Gavage)**	0 mg/kg/day	n/a	n/a	23.5±1.4	24.5±1.5	1.0±0.7
	50 mg/kg/day	n/a	n/a	24.3±2.2	25.1±1.4	0.9±1.6
	100 mg/kg/day	n/a	n/a	24.2±1.7	24.5±1.7	0.4±0.7
	200 mg/kg/day	n/a	n/a	23.4±1.5	23.4±1.3	0.0±0.9
	300 mg/kg/day	n/a	n/a	23.8±2.1	23.6±2.6	-0.2±1.9

**Day 5 (Feed)**	0 ppm	224.1±40.0	n/a	24.0±1.7	24.7±1.6	0.7±0.6
	150 ppm	222.2±60.7	33.3±9.1	23.6±2.0	24.3±1.9	0.8±1.1
	300 ppm	216.6±43.1	65.0±12.9	24.0±2.0	24.7±1.4	0.7±1.0
	1250 ppm	235.7±31.8	294.6±39.7	23.3±1.8	24.9±1.6	1.6±0.6
	2500 ppm	242.4±41.2	605.9±103.0	23.2±1.6	24.4±1.1	1.2±1.2

**Day 28 (Feed)**	0 ppm	267.5±66.4	n/a	23.4±1.9	26.3±1.8	2.8±1.0
	150 ppm	260.3±40.3	39.0±6.0	24.1±1.9	25.5±2.0	1.4±1.1*
	300 ppm	277.2±47.4	83.2±14.2	24.0±1.8	25.2±1.9	1.1±1.2*
	1250 ppm	244.9±29.9	306.1±37.4	23.3±1.7	25.3±1.9	1.9±0.9
	2500 ppm	258.5±36.4	646.2±90.9	22.7±1.2	25.6±0.9	2.9±0.8^

Abbreviations: SD = standard deviation, n/a = not applicable.

1 Feed consumption was calculated based on food consumed after 1 week/ body weights of mice divided by number of days exposed.

2 Body-weight gain determined from individual animal data based on difference between final mean body weight and initial mean body weight.

⁎ Statistically significant decrease compared to concurrent control (Dunnett's test *p*< 0.05).

^ Statistically significant linear trend test (*p*< 0.05).

**Table 2:** In-life animal observations: Mean feed consumption and body weight change in female mice exposed to 4-MeI (Supplemental Data File 1 provides raw data for both male and female mice).

Day of termination	Dose Level	Mean Feed Consumption (g/kg body weight/day)^1^ ±SD	Mean 4-MeI Consumed (mg/kg body weight/day) ± SD	Initial Group Mean Body Weight (g) ± SD	Final Group Mean Body Weight (g) ± SD	Mean Body Weight Gain^2^ (g) ± SD
**Day 2 (Gavage)**	0 mg/kg/day	n/a	n/a	19.0±0.8	18.9±0.9	-0.1±0.5
	50 mg/kg/day	n/a	n/a	19.1±1.1	18.6±1.1	-0.6±0.7
	100 mg/kg/day	n/a	n/a	18.3±1.1	18.3±0.9	0.0±0.8
	200 mg/kg/day	n/a	n/a	18.5±1.0	17.5±1.5	-1.0±0.9
	300 mg/kg/day	n/a	n/a	18.7±1.1	17.8±1.3	-1.0±0.7

**Day 5** **(Feed)**	0 ppm	285.0±102.3	n/a	18.1±1.0	19.4±1.1	1.3±0.7
	150 ppm	334.9±47.4	50.2±7.1	18.3±0.9	19.7±0.7	1.4±0.7
	300 ppm	295.5±91.4	88.6±27.4	18.2±0.9	19.4±0.9	1.2±0.6
	1250 ppm	228.9±40.2	286.1±50.2	18.5±0.8	19.2±0.9	0.8±0.5
	2500 ppm	308.3±61.3	770.7±153.2	18.5±1.1	19.2±1.4	0.7±0.8

**Day 28 (Feed)**	0 ppm	362.6±53.6	NA	18.3±0.8	20.9±1.0	2.6±0.7
	150 ppm	330.9±34.5	49.6±5.2	18.5±1.0	21.4±1.1	2.8±0.7
	300 ppm	367.6±45.9	110.3±13.8	18.5±0.8	20.7±1.1	2.2±0.6
	1250 ppm	309.8±52.8	387.3±66.0	18.3±1.2	20.5±1.3	2.1±0.8
	2500 ppm	299.7±39.8*	749.3±99.4	17.8±0.7	20.3±1.1	2.5±0.9

Abbreviations: SD = standard deviation, n/a = not applicable.

1 Feed consumption was calculated based on food consumed after 1 week/ body weights of mice divided by number of days exposed.

2 Body-weight gain determined from individual animal data based on difference between final mean body weight and initial mean body weight.

⁎ Statistically significant decrease compared to concurrent control (Dunnett's test *p*< 0.05).

Table 3 provides the differential gene expression from feature count data, generated after normalization in DESeq2. Fold change was computed relative to time-specific, vehicle-only control animals. The data provided in this table are the number of differentially expressed genes using a false discovery rate (FDR)-corrected *p*-value of < 0.05, an absolute value of fold change of > 1.2-fold (which had to be lowered from a standard fold change (FC) of 1.5 due to limited signal), and both thresholds applied simultaneously.

**Table 3:** Differential gene expression from feature count data, generated after normalization in DESeq2. Fold change was computed relative to time-specific, vehicle-only control animals. Shown are the number of differentially expressed genes (DEGs) using an FDR-corrected p-value of < 0.05, an absolute value of fold change of > 1.2-fold (which had to be lowered from a standard FC of 1.5 due to limited signal), and both thresholds applied simultaneously. These data are derived from the RNA-Seq count tables in NCBI GEO accession GSE129622.

Sex			Male	Female	Male	Female	Male	Female	Male	Female
Exposure (Gavage)			50 mg/kg-d		100 mg/kg-d		200 mg/kg-d		300 mg/kg-d	
**Day 2**	Liver	FDR<0.05 |FC|>1.2 FDR & |FC|	20 2153 19	1 1374 1	679 2844 577	14 2054 13	0 1672 0	2202 4559 1902	2089 4003 1665	3877 5467 3253
	Lung	FDR<0.05 |FC|>1.2 FDR & |FC|	4 101 3	0 102 0	6 166 4	11 258 10	28 380 24	1012 1374 770	153 873 129	1537 1537 1056

Exposure (feed)			150 ppm		300 ppm		1250 ppm		2500 ppm	

**Day 5**	Liver	FDR<0.05 |FC|>1.2 FDR & |FC|	0 948 0	0 849 0	5 1005 5	1 780 1	20 1333 19	5 883 5	88 1378 82	16 1160 16
	Lung	FDR<0.05 |FC|>1.2 FDR & |FC|	12 287 7	0 55 0	8 285 5	37 278 20	56 186 26	46 289 34	7 156 7	13 155 11

**Day 28**	Liver	FDR<0.05 |FC|>1.2 FDR & |FC|	74 1524 56	0 864 0	4 959 3	0 1025 0	155 1516 102	5 1129 5	21 1335 21	230 1888 182
	Lung	FDR<0.05 |FC|>1.2 FDR & |FC|	1 199 1	1 207 1	23 257 17	42 422 29	54 451 45	7 489 7	9 267 9	20 650 11

**Fig. 1:**
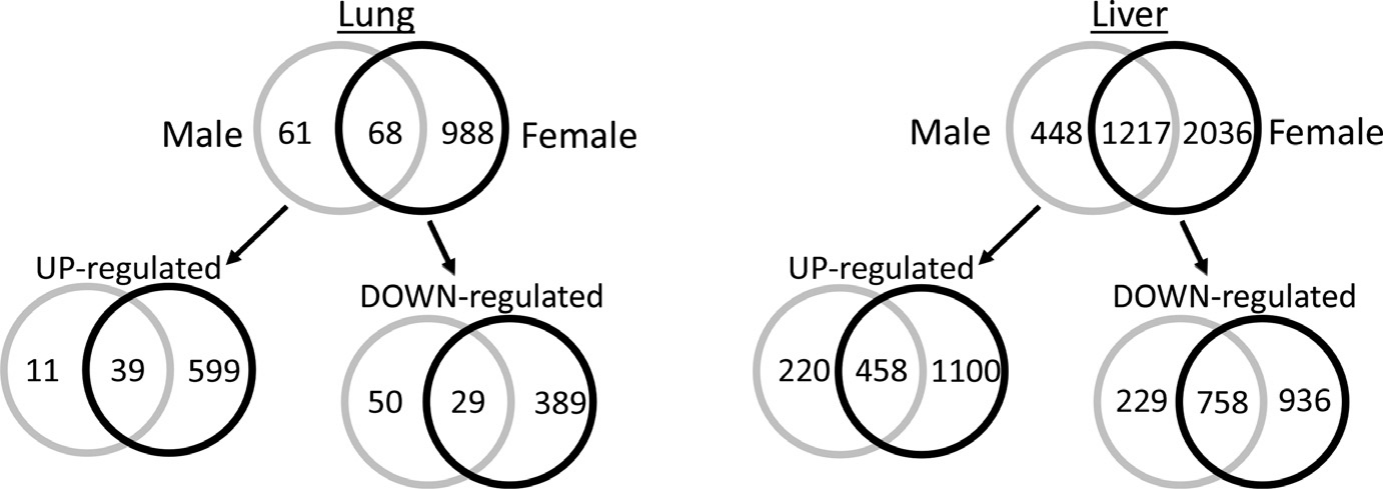
An example of the distribution of differentially expressed genes (DEGs) between male and female mice, broken down based on the overlap from Day 2 maximum dose (300 mg/kg-d) where both sexes exhibit maximal differential expression by any criteria. Genes shown in the Venn diagrams are significant by both FDR<0.05 and a |FC|>1.2-fold at 300 mg/kg-d after administration of 4-MeI for 2 days. While the liver has a larger proportion of genes in common between male and female mice than does the lung, there were still a large number of sex-specific DEGs, particularly in females, when a less stringent criterion of |FC|>1.2 was used.

Fig. 1 provides an example using Venn diagrams showing the distribution of differentially expressed genes (DEGs) between male and female mice exposed to the highest dose level of 4-MeI (300 mg/kg-d) for 2-days of oral gavage dosing. Both sexes exhibit maximal differential expression using any criteria selected. Genes shown in these Venn diagrams were identified as significant by both FDR< 0.05 and a |FC|> 1.2-fold. While the liver has a larger proportion of genes in common between male and female mice than does the lung, there were still a large number of sex-specific DEGs, particularly in females, when a less stringent criterion of |FC|> 1.2 was used.

**Fig. 2:**
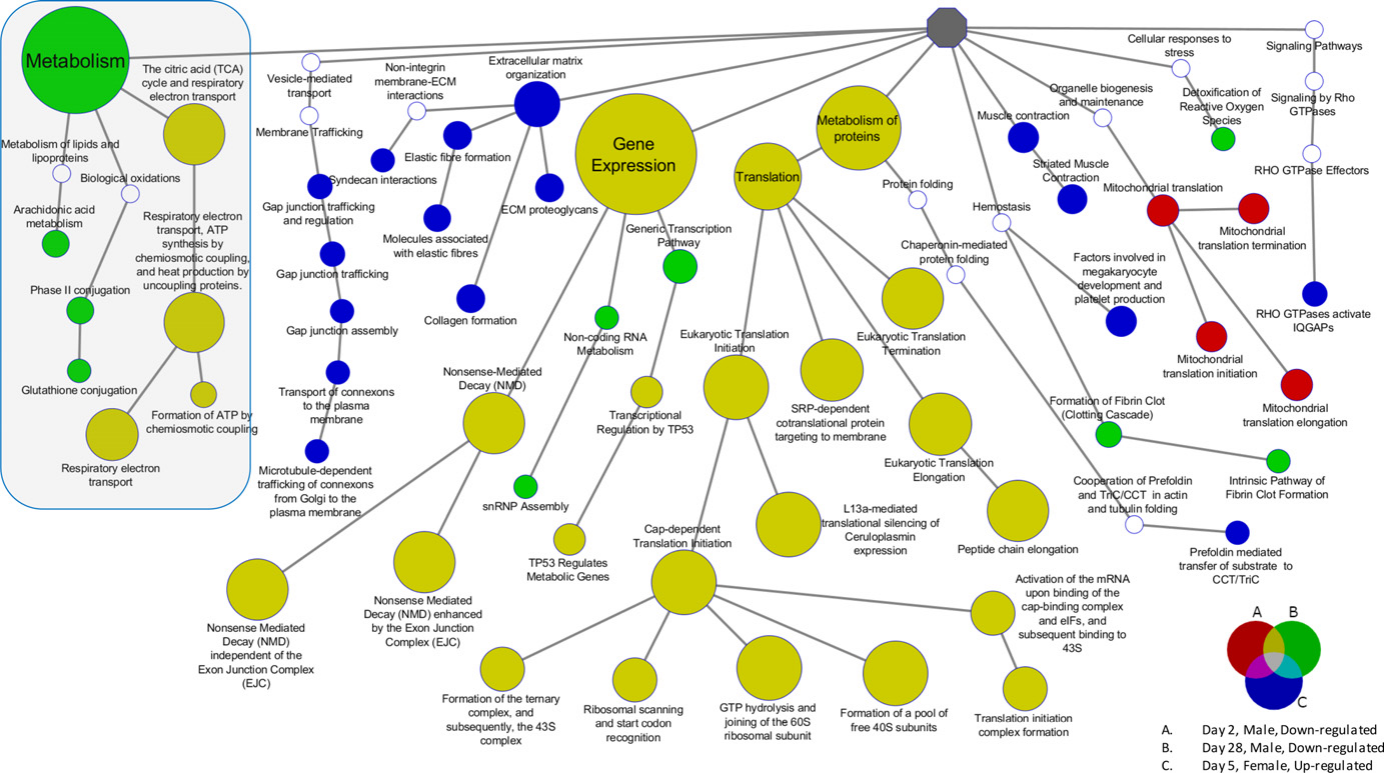
Reactome ontology enrichment for lung using genes rank-ordered by fold change and selecting the top 500 up-regulated and top 500 down-regulated genes from the highest dose level at each time point (2-, 5-, and 28 days). The red nodes (male mice) indicate categories enriched with down-regulated genes at 2 days, the green node (male mice) with down-regulated genes at 28 days, and the blue (female mice) with up-regulated genes at 5 days. The mustard-color nodes (male mice) indicate categories simultaneously enriched for down-regulated genes at both 2 and 28 days. All colored nodes had a minimum of five elements and an enrichment FDR < 0.05 (Fisher's exact test). White nodes were not significantly enriched and are included for continuity of the ontology hierarchy. The shaded area highlights mitochondrial functional pathways and metabolism, including arachidonic acid metabolism.

Fig. 2 provides an example of a map of the ontology enrichment for the lungs of mice exposed to 4-MeI where the genes are rank-ordered by fold change based on the selection of the top 500 genes up-regulated and top 500 genes down-regulated from the highest dose (2 days) or dietary exposure level (5 or 28 days).

**Fig. 3:**
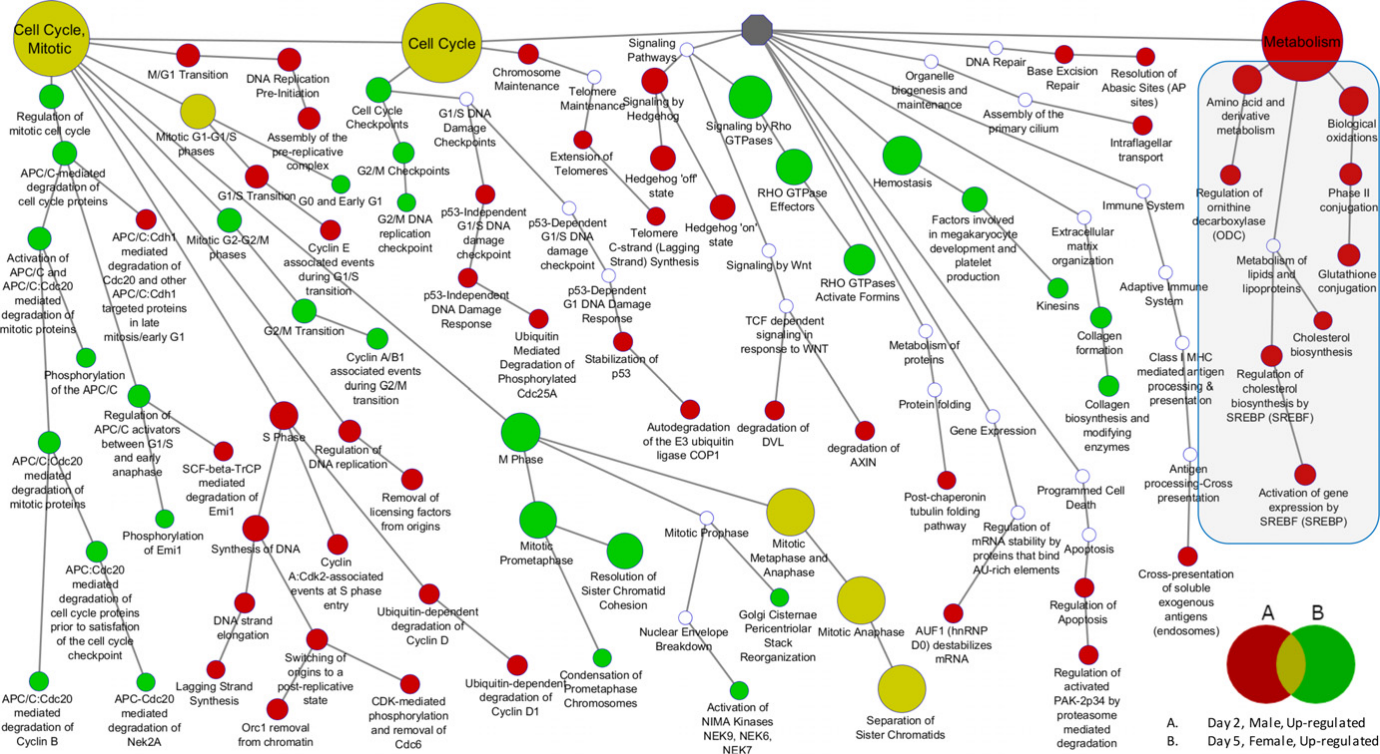
Reactome ontology enrichment for liver using genes rank-ordered by fold change and selecting the top 500 up-regulated and top 500 down-regulated genes from the maximum exposure concentration group. The red nodes (male mice) indicate categories enriched with up-regulated genes that occurred with administration of 4-MeI for 2 days, and the green node (female mice) with up-regulated genes that occurred after 5 days of dietary exposure. The mustard-color node indicates categories simultaneously enriched for both males (2-day) and females (5-day). All colored nodes had a minimum of five elements and an enrichment FDR < 0.05 (Fisher's exact test). White nodes were not significantly enriched and are included for continuity of the ontology hierarchy. The shaded area highlights metabolism processes associated with amino acid metabolism, lipid metabolism, and glutathione metabolism. Temporal differences in the specific processes enriched in cell-cycle processes for each sex are indicated by the mustard-colored nodes downstream of “cell cycle” and “cell cycle, mitotic,” indicating no overlap in enriched elements between the sexes for these different exposure times. Nodes enriched for males at 2 days (red nodes) were not enriched for female mice at 5 days (green nodes), and vice versa.

Fig. 3 provides an example of a map of the ontology enrichment for the liver of mice exposed to 4-MeI where the genes are rank-ordered by fold change based on the selection of top 500 up-regulated and top 500 down-regulated genes from the dose level (2 days) or dietary exposure level (5 days).

Supplemental file 1 provides the final report from the study in which mice were exposed to 4-MeI by oral gavage for 2 days, and then via the diet for 5 or 28 days, prior to collection of liver and lungs for further processing. This report contains the in-life data from the animal study, such as the individual body weights and food consumption information, to summarize the data reported in Tables 1 and 2. It also provide more details on the experimental animal study protocol.

Supplemental file 2 [2_Day2_DGE.xlsx], provides Day 2 liver and lung differential gene expression results (DESeq2) from male and female mice. Tables (four tabs in single Excel file) with gene identifiers include the Log_2_ fold change, the standard error of the fold change, and the p-value and FDR-corrected p-value for each gene.

Supplemental file 3 [3_Day5_DGE.xlsx], provides Day 5 liver and lung differential gene expression results (DESeq2) from male and female mice. Tables (four tabs in single Excel file), with gene identifiers, include the Log_2_ fold change, the standard error of the fold change, and the p-value and FDR-corrected p-value for each gene.

Supplemental file [4_Day28_DGE.xlsx] provides Day 28 liver and lung differential gene expression results (DESeq2) in male and female mice. Tables (four tabs in single Excel file), with gene identifiers, include Log_2_ fold change, the standard error of the fold change, and the p-value and FDR-corrected *p*-value for each gene.

## 2 Experimental Design, Materials and Methods

### 2.1 Animal husbandry

Male and female B6C3F1 mice (n=120/sex, body weight: 16.3–27.6 g, and age: 9 weeks) (CRL International, Inc.) were acclimated for at least 14 days prior to study start; the study was conducted at Integrated Laboratory Services (ILS), Inc. All procedures were in compliance with the Animal Welfare Act Regulations, 9 CFR 1-4, and animals were handled and treated according to the *Guide for the Care and Use of Laboratory Animals* [6].

### 2.2 Test substance

4-MeI was purchased from Sigma Aldrich (Lot Batch, MKBV5083V) and prepared in diet formulations (NTP 2000 rodent diet, Zeigler, Gardners, PA) at CRL (Ashland, OH) and gavage dose formulations at ILS, Inc in sterile USP water at dose concentrations of 0 (water only as vehicle control), 5, 10, 20, and 30 mg/mL. Dose formulations were protected from the light and analyzed under conditions of use and found to be stable.

### 2.3 Study design

Table 4 provides an outline of the study design. For oral gavage, a dose volume of 10 mL/kg was administered each day for 2 days. Dose formulations via feed were *ad libitum* for 5 and 28 days. Animals were evaluated twice daily and once on weekends for mortality/moribundity. Body weights were evaluated at the study start, weekly, and at termination. Food consumption (groups 6–15) was calculated from the start of exposure to the termination date for the 5- and 28-day exposure groups. Animals were euthanized approximately 6 h after the final dose for groups 1–5, and then on Day 5 or Day 28 for animals designated to the dietary study. All animals survived to scheduled termination except for two male mice in Group 5 (300 mg/kg-d, 2-day gavage) which were found dead following the first day of dosing. There were no clinical abnormalities associated with toxicity observed in any animals during the course of the study. Of the eight animals sampled, the six with the highest yield and quality of recovered RNA were used for sequencing. The complete in-life report with clinical observational data is available as Supplemental Data File 1.

**Table 4:** The group number, number of animals per group, test substance and dose level, dose route, and day of study termination are identified. Six animals per group were analyzed only for changes in transcriptomics. (Note: animals 070 and 071 were found dead after dose administration on Day 0 and, thus, are absent from the data table).

Group Number	Sex (M/F)	Test Substance	Test-Article Dose Level	Dose Route	Day of Termination
1	8/8	Vehicle Control	0 mg/kg/day	Oral-gavage	2
2	8/8	4-MeI	50 mg/kg/day	Oral-gavage	
3	8/8	4-MeI	100 mg/kg/day	Oral-gavage	
4	8/8	4-MeI	200 mg/kg/day	Oral-gavage	
5	8/8	4-MeI	300 mg/kg/day	Oral-gavage	

6	8/8	Vehicle Control	0 ppm	Oral-diet	5
7	8/8	4-MeI	150 ppm	Oral-diet	
8	8/8	4-MeI	300 ppm	Oral-diet	
9	8/8	4-Me	1250 ppm	Oral-diet	
10	8/8	4-MeI	2500 ppm	Oral-diet	

11	8/8	Vehicle Control	0 ppm	Oral-diet	28
12	8/8	4-MeI	150 ppm	Oral-diet	
13	8/8	4-MeI	300 ppm	Oral-diet	
14	8/8	4-MeI	1250 ppm	Oral-diet	
15	8/8	4-MeI	2500 ppm	Oral-diet	

At the end of the study, the right lung was harvested for gene expression analysis. The lung was perfused with RNAlater, and a section of the left liver lobe was cubed and fully immersed in RNAlater (≥ 5 volumes) and stored at 2–8°C for 1–30 days and then at -15°C to -25°C indefinitely. The left lung was perfused with 10% neutral buffered formalin (NBF), and the remaining liver lobe was immersed in NBF for 18–24 h and transferred to 70% histology-grade alcohol prior to paraffin embedding. RNA was extracted from liver and lung from each animal, cDNA libraries were prepared, transcriptomes were sequenced using next-generation sequencing, and FASTQ files were prepared prior to data analysis.

### 2.4 RNA sequencing

Sequencing was carried out using 1- to 2-µg total cellular RNA using Illumina standard procedures for their TrueSeq® stranded mRNA HT kits. Sequencing was performed on an Illumina NextSeq 500, and binary base call (BCL) files were uploaded to Illumina BaseSpace for processing and FASTQ file generation. After preparation of the mRNA from eight animals per exposure group, the six samples with the highest yield were used for sequencing. Table 5 provides a summary of the samples of liver and lung collected from the male and female mice for the RNA-Seq experiment, including the day and 4-MeI concentrations and the number of biological replicates collected for analysis.

**Table 5:** A total of six mice per condition (sex, dose, and time) (Table 4) were used for the RNA-Seq experiment (the six animals with the best total RNA yield among the eight animals per exposure group). The raw data (FASTQ files in NCBI sequence read archive (SRA) as part of GEO accession GSE129622) consist of four pairs of paired-end read files per sample, while the mm10 feature count data in the NCBI GEO accession consist of total counts per genomic feature per sample (six tab-delimited text files, one for each sex and tissue at each of the three sample time points). The vehicle control (VC) was sterile water for the gavage study and untreated feed for the dietary study).

Tissue	Liver					Lung				
Sex	Male and Female B6C3F1 mice					Male and Female B6C3F1 mice				
2-Day (gavage, mg/kg-d)	0 (VC)	50	100	200	300	O (VC)	50	100	200	300
5- & 28-Day (feed, ppm)	0 (VC)	150	300	1250	2500	0 (VC)	150	300	1250	2500

No. of biological replicates	Six per exposure, sex, and time					Six per exposure, sex, and time, except for females at 2500 ppm, 5 days where n=5				

Total Samples for RNA-Seq	180					179				
FASTQ files (four pairs per sample)	1440					1432				

#### 2.4.1 FASTQ file processing

The design of the sequencing experiment meant that each biological sample consisted of four barcoded sets of reads, with a pair of FASTQ files (forward and reverse reads) for each barcode set. To eliminate low quality reads from processing, each FASTQ file was processed by read trimming, where each sequence read was trimmed from both ends to eliminate all bases with a PHRED33 score (measure of quality of nucleobases generated by automated DNA sequencing) of less than 21 [7]. Also, any read of less than 65bp generated as a result of trimming was discarded. For the samples from 4-MeI exposed mice, this typically eliminated less than 0.5% of the total reads in any given FASTQ file, thus effectively only eliminating the few poor-quality reads in a sample.

After trimming, each pair of read files (FASTQ file) were mapped to the UCSC mm10 reference genome (http://genome.ucsc.edu) using the short-read mapping algorithm BOWTIE2 and indexed reference genomes provided publicly as a resource by Illumina's iGenome project [8]. Each mapped pair of FASTQ files produced a single SAM (sequence alignment map) format output file [9]. These were, in turn, sorted by genomic coordinates, and then merged into a single mapped read BAM (binary alignment map) file, to produce a single mapped read file for each biological sample.

Raw gene expression data were extracted from the BAM files by counting each pair of reads that maps to an annotated genomic feature in the reference mm10 using the Python tool HTSeq [10]. Once each biological sample was counted, the counts are merged into a single tab-delimited table for statistical processing. Both FASTQ files and the count tables as text files were deposited in the NCBI GEO expression database, accessible under accession GSE129622.

#### 2.4.2 Differential gene expression analysis

The tabulated genomic feature count data was processed in DESeq2, a BioConductor package (ver. 3.4) in the open-source statistical language R (ver.3.3.2) [11]. DESeq2 uses a dispersion correction of the count data based on the negative binomial distribution and a maximum likelihood model to impute the prior data distribution for statistical testing. Empirical Bayesian statistics are applied to linear combinations of factors to test differential expression for multiple contrasts simultaneously. To avoid bias and unnecessary computation in the dispersion correction, the data set was pre-filtered to exclude any annotated genomic feature for which there were no counts in any biological sample. The final output of DESeq2 is a table of estimated Log_2_ fold change, p-values for the defined contrasts tested, and Benjamini-Hochberg corrected false discovery p-values (FDR) [12].

We determined the significance of differential expression using multiple thresholds, either singly or in combination. A statistical threshold of an FDR< 0.05 is a commonly used significance threshold in whole transcriptomic analysis. Additionally, some minimum magnitude of change in gene expression is typically applied as a selection criterion. In this study, with six replicates per dose and time, fold change thresholds of 1.2-fold, up- or down-regulated (|FC|> 1.2 fold), was applied, because the more commonly applied FC threshold of 1.5-fold was not sensitive enough. A fold change of 1.2-fold is equal to a Log_2_ fold change of 0.263. Finally, the application of both a statistical threshold and the smaller FC criterion (FC=1.2) (FDR< 0.05 & |FC|> 1.2 fold) permitted identification of a larger numbers of genes whose differential expression from controls was statistically significant, allowing a better opportunity to identify enriched pathways. The complete differential expression tables that provide the data described above are provided as Supplemental Data Files 2, 3, and 4 (Supplemental_2_Day2_DGE.xlsx, Supplemental_3_Day5_DGE.xlsx, and Supplemental_4_Day28_DGE.xlsx), for data from male and female mouse liver and lung following exposure to various concentrations of 4-MeI for 2 days, 5 days, and 28 days, respectively.

Reactome ontology enrichment was performed using an in-house software tool (GoFigureMaps) that performs a Fisher's exact test of over-representation of query genes relative to defined pathway elements. This software produces a graphical representation of the ontology enrichment in the context of the ontology hierarchy of cellular pathways, referred to as a bubblemap [13,14].

## Ethics Statement

The Animal Study final report described in Supplemental Data File 1 was conducted at ILS. ILS has an Office of Laboratory Animal Welfare (OLAW) Assurance (A3490-01), and therefore, it uses the standards of the ILAR (2011) *Guide for the Care and Use of Laboratory Animals* [6], the PHS (2015) *Policy for Humane Care and Use of Laboratory Animals* [15], and USDA, (2020) Animal Welfare Act [16].

## CRediT authorship contribution statement

**Michael B. Black:** Methodology, Formal analysis, Data curation, Writing – original draft. **Melvin E. Andersen:** Conceptualization, Investigation, Writing – review & editing, Funding acquisition. **Salil N. Pendse:** Formal analysis, Data curation, Writing – review & editing. **Susan J. Borghoff:** Writing – review & editing. **Michael Streicker:** Investigation. **Patrick D. McMullen:** Writing – review & editing, Project administration.

## Declaration of Competing Interest

The authors declare the following financial interests/personal relationships, which may be considered as potential competing interests:

This work was funded by the American Beverage Association. No authors received personal fees. ScitoVation, ToxStrategies, and ILS provide scientific consulting services to various entities in the private sector and conduct research or testing related to food and beverage safety.

## Appendix Supplementary materials

Image, application 1

Image, application 2

Image, application 3

Image, application 4
