# Supplementary material for: RNA-sequencing (transcriptomic) data collected in liver and lung of male and female B6C3F1 mice exposed to various dose levels of 4-methylimidazole for 2, 5, or 28 days
Source: Data Brief. 2021 Sep 23;38:107420. doi: 10.1016/j.dib.2021.107420 (PMC8502903; doi:10.1016/j.dib.2021.107420)
Supplement: Supplementary file 1 [file mmc1.pdf]

100 Capitola Dr., Suite 106
Research Triangle Park, NC 27713
www.scitovation.com

Supplemental file 1: In life study report for data in brief:

Michael B. Black, Melvin E. Andersen, Salil N. Pendse, Susan J. Borghoff, Patrick D. McMullen.

Analysis of Gene Expression (RNA-Seq) and Cell Proliferation in Livers and Lungs of Male and Female B6C3F1 Mice Exposed to Various Dose Levels of 4-Methylimidazole for 2, 5, or 28 days

FINAL REPORT

Study Title

Short Term Toxicity and Genomics Study of 4-Methylimidazole in B6C3F1 Male and Female Mice

ILS Project-Study Numbers

50074.0001

Author

Michael A. Streicker, B.S., LATG

Performing Laboratory

Integrated Laboratory Systems, Inc.

601 Keystone Park Drive, Suite 200

Morrisville, NC 27560 USA

Sponsor

Scitovation, LLC

6 Davis Drive

RTP, NC 27709

Date of Submission

28 February 2017

Final Report Approval

Michael A. Streicker, B.S., LATG	Date
Study Director	
Integrated Laboratory Systems, Inc.	

This Final Report was reviewed by:

Cheryl Hobbs, Ph.D.	Date
Director of Toxicology	
Integrated Laboratory Systems, Inc.	

______________________________ ____________________

Leslie Recio, Ph.D., DABT Date

Vice President, Research and Development

Integrated Laboratory Systems, Inc.

TABLE OF CONTENTS

STUDY SUMMARY 8

INTRODUCTION 9

1.1 Background 9

1.2 Purpose 10

1.3 Sponsor 10

1.4 Testing Facility 10

1.5 Study Dates 11

TEST ARTICLE 11

2.1 Test Article: 4-Methylimidazole 11

2.2 Vehicle (Feed): NTP 2000 13

2.3 Vehicle (Oral Gavage): Sterile Water 13

2.4 Archival Samples 14

2.5 Dose Formulations Analysis 14

EXPERIMENTAL DESIGN 15

3.1 Test System 15

3.2 Animal Husbandry 16

3.3 Allocation 18

3.4 Group Designation 18

3.5 Dose Administration 19

3.6 In-Life Animal Observations 20

3.7 Termination 21

3.8 Statistical Analysis 22

4.1 Dose Formulation Analysis 23

5.1 In-Life Animal Observations 23

6.1 Necropsy Gross Observations 28

SUMMARY 28

KEY PERSONNEL 29

Tables:

Table 1. Group Designation, Animal Identification, and Dose Levels 18

Table 2. Male Mice: Body Weight Change 25

Table 3. Female Mice: Body Weight Change 26

Table 4. Male Dose Group Mean Food Consumption 27

Table 5. Female Dose Group Mean Food Consumption 27

Appendices:

Appendix I: Clinical Observation Data 30

Appendix II: Body Weight Data 64

Appendix III: Food Consumption Data 97

Appendix IV: Necropsy Data 102

Appendix V: Certificate of Analysis and CRL Formulations Report 114

Appendix VI: p Value Table 174

Appendix VII: Study Protocol and Amendments 177

STUDY SUMMARY

Male and female B6C3F1 mice (120/sex) were allocated to one of fifteen designated dose groups. The animals were administered 0, 50, 100, 200 or 300 mg/kg 4-methylimidazole in water via oral gavage for 2 days or 0, 150, 300, 1250 or 2500 ppm via feed for 5 or 28 days. Clinical observations and body weights with food consumption were recorded weekly. On Days 2, 5 or 28, the animals were humanely euthanized. Blood was collected and held for potential analysis. Lung, liver, kidneys (separately), testes (separately), ovaries (separately) and brain were cubed and fully immersed in RNAlater. Liver and lung were fixed in 10% neutral buffered formalin.

There were no clinical observations noted during the study that would be associated with toxicity. There were no statistical changes in final body weight or body weight gain of groups exposed to 4-methylimidazole for 2 or 5 days compared to the control groups; however, a statistically significant lower body weight gain was measured in the male 150 and 300 ppm dose groups with a corresponding statistically positive (decreasing) linear trend test at the 28-day time point. There was a statistically significant decrease in measured feed consumed by female mice exposed to 2500 ppm of 4-methylimidazole for 28 days compared to the control group. No other statistical differences in measured food consumption were found for male or female mice. There were minimal necropsy observations relevant to 4-methylimidazole noted for either males or females at any of the time points at any tested dose level.

RNA was extracted from liver and lung from each animal, cDNA libraries were prepared and transcriptomes sequenced using next generation sequencing, and FASTQ files were delivered to ScitoVation for analysis.

INTRODUCTION

1.1 Background

4-Methylimidazole (4-MEI) is present in caramel colorings manufactured with ammonia catalysts and has potential human exposure in a wide variety of food and beverage products. In previous studies, groups of male and female rats and mice were fed diets containing 4-MEI for durations of 15 days, 14 weeks, or 2 years. Results of the 15-day study indicated a no-observed-adverse-effect-level (NOAEL) of 2500 ppm in rats and mice (highest dose tested). In the 14-week study, NOAELs of 1250 ppm (male rats), 5000 ppm (female rats), and 10,000 ppm (male and female mice; highest dose tested) were determined based primarily on the results of tissue histopathology. Alterations related to 4-MEI exposure included anemia, hepatocytic vacuolation, testicular degeneration (rats), prostatic atrophy (rats), and renal degeneration and dilation (mice).

For the 2-year study, 4-MEI concentrations in feed were 0, 625, 1250, or 2500 ppm (male rats), 0, 1250, 2500 or 5000 ppm (female rats), and 0, 312, 625, or 1250 ppm (male and female mice). Results indicated no evidence of carcinogenic activity in male rats, equivocal evidence of carcinogenicity (mononuclear cell leukemia) in female rats, and clear evidence of carcinogenicity (alveolar/bronchiolar adenoma or carcinoma) in male and female mice. Nonneoplastic effects in the 2-year study were observed in the liver of male and female rats (histiocytosis, chronic inflammation, focal fatty hepatoctye changes, and eosinophilic focus) and in the lungs of female mice (alveolar epithelium hyperplasia). 4-MEI was negative in genetic toxicology studies conducted by the NTP (Ames *Salmonella* test and rat or mouse micronucleus

tests).

In this study, a short-term in-life toxicity study with 4-MEI and concurrent measurement of genomic responses in target tissues was conducted. The animal model was the B6C3F1 mouse, since this species was susceptible to the formation of lung tumors following lifetime exposure of 4-MEI. The target tissues were the lung and liver. The study assessed dose-response and temporal relationships. Doses of 4-MEI were selected based on effects observed in previous toxicity studies with 4-MEI. Altered gene expression was compared to tissue cell proliferation and histopathology. The pattern and dose response of observed alterations provided signatures for 4-MEI effects. These signatures should identify early events occurring in target tissues that would be of value for understanding the mode-of-action and dose response of 4-MEI. The information gained from genomic screening could then be used for designing more specific *in vivo* and/or *in vitro* 4-MEI studies targeted to the understanding of induction of mouse lung tumors and relevance of effects to humans.

1.2 Purpose

The purpose of this study was to assess dose-response and time-dependent tissue and cellular responses following 4-MEI exposure. The collection of data on both gene expression and toxicological endpoints (histopathology and cell proliferation), allowed characterization of the relationship of gene expression signatures with pathway alterations in tissues associated with 4-MEI exposure.

1.3 Sponsor

ScitoVation, LLC

6 Davis Drive

RTP, NC 27709

Study Monitor

Chris Bartlett

Telephone No.:	(919) 558-1400
E-mail:	cbartlett@scitovation.com

1.4 Testing Facility

Shipping Address:	601 Keystone Park Drive, Suite 200
	Morrisville, NC 27560 USA
Mailing Address:	P.O. Box 13501

Research Triangle Park, NC 27709 USA

Study Director

Michael A. Streicker, B.S., LATG

Telephone No.:	(919) 281-1110 ext. 721
Facsimile No.:	(919) 281-1118
E-mail:	mstreicker@ils-inc.com

1.5 Study Dates

Animal Arrival Date:	14 September 2016
Experimental Start Date:	29 September 2016
Experimental In-Life Termination Date:	27 October 2016

TEST ARTICLE

2.1 Test Article: **4-Methylimidazole**

CAS No.:	822-36-6
Source:	Sigma Aldrich
Lot/Batch No.:	MKBV5083V

ILS Repository No.:	16-135
Description:	Pale Yellow Crystal
Purity:	98%
Expiration Date:	27 July 2017
Dose Formulation:	Diets containing 4-MEI were prepared at Charles River (CRL) (Ashland, OH) using a twin-shell blender according to CRL SOPs in NTP 2000 feed at dose concentrations of 0, 150, 300, 1250 and 2500 ppm. The feed for the 28-day dose groups was prepared approximately every two weeks. Prepared diets were shipped under ambient conditions to ILS. Once received at ILS, feed was stored at ambient conditions for dose administration or stored refrigerated for subsequent feedings.<br><br>4-MEI was prepared once at ILS in sterile USP water at dose concentrations of 0, 5, 10, 20 and 30 mg/mL and aliquoted into vials to be used daily during the study.
Storage:	
Test Article:	Room temperature and protected from light
Dose Formulation:	Between 1 and 10°C (~4°C) and protected from light until placed in feed jars at room temperature or used for gavage dosing.

Stability:

Dose Formulation: 4-MEI in NTP 2000 feed has been shown to be stable for up to 36 days and up to 3 days in water stored between 1 and 10°C.

2.2 Vehicle (Feed): NTP 2000

Source: Zeigler (Gardners, PA)

Lot/Batch No.: 01 July 2016

Storage: Room temperature

2.3 Vehicle (Oral Gavage): Sterile Water

Source: Patterson Veterinary Supply

Lot/Batch No.: A1605052

ILS Repository No.: 16-168

Description: Clear liquid

Storage: Room temperature and protected from light

2.4 Archival Samples

Approximately 10 mg of 4-MEI, 1 mL of oral gavage dose formulations, and 10 mg of feed dose formulations were stored between 0 and -30°C. After acceptance of the study report by the Sponsor, archival dose formulation samples will be discarded. Test and control articles will be maintained by ILS for five years following finalization of the study report. Transfer of archival samples may be requested by the Sponsor prior to the end of the five-year archival period. At the end of the five-year archival period, the Sponsor will be contacted for direction of appropriate disposition of archival samples remaining at ILS.

2.5 Dose Formulations Analysis

Test article diet dose formulations were prepared at Charles River and oral gavage doses were prepared at ILS. All samples were analyzed at Charles River. Test diet formulations were prepared in NTP 2000 meal and approximately 50 gram samples were collected from each dose level (including controls) at the time that the formulations were prepared. Oral (gavage) test material formulations were prepared in water and approximately 5 mL samples from each dose level (including controls) were collected at the time that the formulations were prepared. A portion of each diet sample was tested upon formulation. Another portion of each diet sample was held at room temperature for at least 10 days, and then tested to determine that stability of 4-MEI in the NTP 2000 diet. The remaining portion of each diet sample was refrigerated for at least 7 days starting on the day of formulation at CRL; after at least 7 days, it was removed from the refrigerator and held at room temperature for at least 9 days and then tested. Stability analysis was performed on feed samples at least 1, 7 and 16 days post formulation in various storage conditions and on oral gavage samples at 1 and 3 days post formulation. The oral gavage samples were kept at 0 and 10°C. The feed samples and oral gavage samples were stored by Charles River under the same conditions as the dose formulations used by ILS for the studies.

Prior to initial dosing/exposure, an additional three sets of samples (10 grams for each dose level of test diet and 5 mL for each dose level of oral test material formulations) were collected and sent to Charles River for analysis of concentration acceptability. The three sets of test diet samples include: (1) one set collected at the initiation of the 5-day study, (2) one set collected at the initiation of the 28-day study, and (3) one set collected from the second batch of diet at the start of the third week of the 28-day study.

Analysis of the aqueous and diet formulations for concentration acceptability was performed by Charles River within 24 hours of receipt of samples using a qualified laboratory method

(865.RDT.01, 865.WFI1.01). Draft results were transmitted to the Study Director and a summary report signed by the Principal Investigator was provided for inclusion in the final study report.

Principal Investigator- (Dose Formulation Analyses):

Archana Akalkotkar, PhD.

Charles River

1407 George Rd.

Ashland, OH 44805 USA

Records Retention

All data generated by Charles River was archived by Charles River following Charles River SOPs.

EXPERIMENTAL DESIGN

For the study, 120 male and 120 female B6C3F1 mice were allocated to one of 15 designated dose groups. The animals in groups 1-5 were administered one of five dose levels of 4-MEI or the vehicle control (water) for two consecutive days via oral gavage. The animals in groups 6-15 were exposed to one of five dose levels of 4-MEI or the vehicle control (NTP 2000 feed) for 5 or 28 consecutive days in feed.

3.1 Test System

Species: Mice, *Mus musculus*

Strain: B6C3F1

Source:	Charles River Laboratories International, Inc.
Number/Sex:	120 males and 120 females
Acclimation:	At least fourteen days
Age at administration:	9 weeks of age
Weight at administration:	16.3-27.6 grams
Identification:	Each animal was uniquely identified by ear punch prior to the start of the study. Until the animals were ear punched, they were identified by the temporary numbers located on the animals' enclosures.
Justification:	This study was conducted using the same animal model used in a 2-year bioassay to help elucidate the toxicity and genomic effects of the test substance and the relevance of this response to humans.

3.2 Animal Husbandry

All procedures were in compliance with the Animal Welfare Act Regulations, 9 CFR 1-4, and animals were handled and treated according to the *Guide for the Care and Use of Laboratory Animals* (ILAR, 2011).

Housing:	One per cage
Cage Type:	Polycarbonate with micro-isolator tops
Cage Size:	17 cm wide by 28 cm long (476 cm^2 area) and 13 cm high
Bedding:	Absorbent heat-treated hardwood bedding (Northeastern Products Corp., Warrensburg, NY)
Cage Changes:	Once per week
Diet:	NTP 2000 (Zeigler, Gardner, PA) was provided *ad libitum.*
Analysis:	The manufacturer's composition formula was included in the raw data and reviewed prior to animal arrival.
Water:	Reverse osmosis (RO) treated tap water (City of Durham, NC) *ad libitum*
Supplied:	Polycarbonate water bottles with stainless steel sipper tubes
Analysis:	The results of the current annual comprehensive chemical analyses of RO water from National Testing Laboratories, Inc. (Cleveland, OH) were reviewed prior to initiation of the study and was included in the raw data.
Water Bottle Changes:	Once per week

Temperature: 21.2-22.8°C

Humidity: 37.8-66.4%

Lighting: 12/12 hour light/dark cycle

Enrichment: Nestlets (Ancare, Bellmont, NY)

Analysis: The manufacturer's analytical results of the nestlets was included in the raw data and reviewed before study start.

3.3 Allocation

The animals were assigned to a dose group using a procedure that stratifies animals across groups by body weight such that mean body weight of each group was not statistically different from any other group using analysis of variance (ANOVA) (Statistical Analysis System version 9.2, SAS Institute, Cary, NC).

3.4 Group Designation

Table 1. Group Designation, Animal Identification, and Dose Levels

Group Number	Sex (M/F)	Animal Identification (M/F)		Dose	Test Article Dose Level	Dose Route	Day of Termination
1	8/8	001-008	009-016	Vehicle Control	0 mg/kg/day	Gavage	2

Group Number	Sex (M/F)	Animal Identification (M/F)		Dose	Test Article Dose Level	Dose Route	Day of Termination
2	8/8	017-024	025-032	4-MEI	50 mg/kg/day	Gavage	2
3	8/8	033-040	041-048	4-MEI	100 mg/kg/day	Gavage	2
4	8/8	049-056	057-064	4-MEI	200 mg/kg/day	Gavage	2
5	8/8	065-072	073-080	4-MEI	300 mg/kg/day	Gavage	2
6	8/8	081-088	089-096	Vehicle Control	0 ppm	Feed	5
7	8/8	97-104	105-112	4-MEI	150 ppm	Feed	5
8	8/8	113-120	121-128	4-MEI	300 ppm	Feed	5
9	8/8	129-136	137-144	4-MEI	1250 ppm	Feed	5
10	8/8	145-152	153-160	4-MEI	2500 ppm	Feed	5
11	8/8	161-168	169-176	Vehicle Control	0 ppm	Feed	28
12	8/8	177-184	185-192	4-MEI	150 ppm	Feed	28
13	8/8	193-200	201-208	4-MEI	300 ppm	Feed	28
14	8/8	209-216	217-224	4-MEI	1250 ppm	Feed	28
15	8/8	225-232	233-240	4-MEI	2500 ppm	Feed	28

3.5 Dose Administration

For groups 1-5, dose formulations were administered via oral gavage at a dose volume of 10 mL/kg body weight for two consecutive days. Dose volume was based on individual animal daily body weight. Dose formulations were placed on a stir plate at least 30 minutes prior to dosing and continuously stirred. The second dose occurred 24 hours (± 30 minutes) from the previous dose. The dosing sequence was stratified across dose groups; one animal from each group and then repeated until all animals were dosed.

For groups 6-10, dose formulations were administered via feed *ad libitum* for 5 days. For groups 11-15, dose formulations were administered via feed *ad libitum* for 28 days.

3.5.1 Justification of Route of Administration

An oral route of administration is consistent with the primary route of human exposure.

3.5.2 Justification of Dose Levels

The dose levels were selected on the basis of results of an NTP 2-year bioassay.

3.5.3 Disposal of Dose Formulations

Dose formulations were disposed of as hazardous material.

3.6 In-Life Animal Observations

Mortality/Moribundity: Twice daily on weekdays, once daily on weekends

Clinical Observations: Observed within two days of arrival, for allocation of animals to a dose group, weekly, and at termination. Animals in groups 1-5 had an observation collected prior to dosing each day.

Body Weights: Collected within two days of arrival, for allocation of animals to a dose group, at study start, weekly, and at termination. Animals in groups 1-5 had a body weight collected prior to dosing each day.

Food Consumption: For animals in groups 6-15, feed jars were weighed on the day of body weight collection each week. Feed consumption (g/kg body

weight/day) was calculated from the start of exposure to the necropsy date for the 5 (5 day consumption) and 28 (weekly consumption for each week) day time points.

3.7 Termination

Scheduled: For groups 1-5, approximately 6 hours (± 30 minutes) after the final dose administration, animals were euthanized by CO_2 asphyxiation and death confirmed by exsanguination.

For groups 6-10, on Day 5 animals were euthanized by CO_2 asphyxiation and death confirmed by exsanguination.

For groups 11-15, on Day 28 animals were euthanized by CO_2 asphyxiation and death confirmed by exsanguination.

Tissue Collection: Blood (maximum volume) was collected via cardiac puncture into EDTA tubes (500 µl tubes). Tubes were spun at 1500 g for 10 min at 4°C and the plasma removed and stored at or below -70°C for analysis.

For each animal, the right lung lobes were harvested and perfused with RNAlater (≥5 volumes) for subsequent gene expression analysis, cubed, and stored at 2-8°C for 1-30 days and then stored at -15°C to -25°C indefinitely. The left lung lobe was perfused with 10% neutral buffered formalin (NBF).

A section of the left lobe of the liver was cubed, fully immersed in RNAlater (≥5 volumes), stored at 2-8°C for 1-30 days and then stored at -15°C to -25°C indefinitely. The remaining lobe was fixed in 10% NBF for 18-24 hours and then transferred to 70% histology grade alcohol prior to paraffin embedding. The remaining lobes of the liver

were cubed, pieces frozen individually, all frozen pieces placed in a cryovial and flash frozen in liquid N_2, and placed on dry ice until stored at or below -70°C for possible subsequent analysis.

The kidneys (separately), testes (separately), ovaries (separately) and brain were cubed, fully immersed in RNAlater in cryovials and placed on wet ice until stored at 2-8°C for 1-30 days and then stored at -15°C to -25°C indefinitely.

Specific information describing the tissue genomic analysis was included in Study Specific Procedure No. 1 .

3.8 Statistical Analysis

Individual animal data and group mean and standard deviations were calculated and reported.

For final body weight, body weight gain and feed consumption measurements, data were analyzed using Statistical Analysis System version 9.2 (SAS Institute, Cary, NC). Homogeneity of variance was analyzed using Levene's test, and normality of the vehicle control data was assessed using a Shapiro-Wilk test. Homogenous data were analyzed using a one way analysis of variance (ANOVA) test and test article administered groups were compared to the vehicle control group using Dunnett's multiple comparison test. Dose-dependent changes were evaluated using a linear regression model.

Body weight gain data for control males exposed 5 days via feed were not normally distributed and therefore an inverse transformation was used to derive a homogenous set of data. All other data were homogenous and normally distributed.

All statistical analysis of the genomics data will be conducted by ScitoVation.

RESULTS

4.1 Dose Formulation Analysis

The results of the analysis of the dose formulations for gavage were acceptable for both the concentration and stability. The initial concentration analysis indicated that the dose formulations for all dose levels in the feed were out of specification. After discussion with the Sponsor and the technical staff at CRL, the issue is believed to have been a technical problem with the extraction of the test article from the feed for analysis. Subsequently, additional sonication of the samples and reanalysis was performed and the results were between 77.4 and 82.1% of nominal for the dose groups. During a conference call on 29 Sept 2016, it was decided to move forward with the feed portion of the study. Based on the concentration results generated from the test article stability evaluation in which 4-MEI in some dosed-diet samples (150 and 300 ppm) was measured at higher concentrations than originally measured, it is believed that the animals received doses within a reasonable range of the nominal dose levels.

5.1 In-Life Animal Observations

Mortality/Moribundity

All animals survived to the scheduled termination except for animals 070 and 071 (300 mg/kg/day) which were found dead on Day 0 post dose. No sign of dosing error was noted during the necropsy.

Clinical Observations

There were no abnormal clinical observations noted during the course of the study that are associated with toxicity. Individual animal clinical observation data are listed in Appendix I.

Body Weights

Group mean initial and final body weights, and body weight gain for animals euthanized following 2, 5 and 28 consecutive days of 4-MEI exposure are presented in Table 2 (males) and Table 3 (females). There were no statistical changes in final body weight or body weight gain of groups exposed to 4-MEI compared to the concurrent control groups for the 2- and 5-day time points; however, the male 150 and 300 ppm dose groups exhibited a statistically significant lower body weight gain at the 28-day time point. There was also a significant decreasing trend for body weight gain in the male mice fed 4-MEI in their diet for 28 days. Individual animal body weight data are listed in Appendix II.

Table 2. Male Mice: Body Weight Change

Group Number	Dose Level	Dose Route	Day of Termination	Initial Group Mean Body Weight (g) ± SD	Final Group Mean Body Weight (g) ± SD	Mean Body Weight Gain# (g) ± SD
1	0 mg/kg/day	Gavage	2	23.5±1.4	24.5±1.5	1.0±0.7
2	50 mg/kg/day	Gavage	2	24.3±2.2	25.1±1.4	0.9±1.6
3	100 mg/kg/day	Gavage	2	24.2±1.7	24.5±1.7	0.4±0.7
4	200 mg/kg/day	Gavage	2	23.4±1.5	23.4±1.3	0.0±0.9
5	300 mg/kg/day	Gavage	2	23.8±2.1	23.6±2.6	-0.2±1.9
6	0 ppm	Feed	5	24.0±1.7	24.7±1.6	0.7±0.6
7	150 ppm	Feed	5	23.6±2.0	24.3±1.9	0.8±1.1
8	300 ppm	Feed	5	24.0±2.0	24.7±1.4	0.7±1.0
9	1250 ppm	Feed	5	23.3±1.8	24.9±1.6	1.6±0.6
10	2500 ppm	Feed	5	23.2±1.6	24.4±1.1	1.2±1.2
11	0 ppm	Feed	28	23.4±1.9	26.3±1.8	2.8±1.0
12	150 ppm	Feed	28	24.1±1.9	25.5±2.0	1.4±1.1*
13	300 ppm	Feed	28	24.0±1.8	25.2±1.9	1.1±1.2*
14	1250 ppm	Feed	28	23.3±1.7	25.3±1.9	1.9±0.9
15	2500 ppm	Feed	28	22.7±1.2	25.6±0.9	2.9±0.8^

Abbreviation: SD- standard deviation

*Statistically significant decrease compared to concurrent control (Dunnett's test $p<0.05$)

#Body weight gain determined from individual animal data

^statistically significant linear trend test ($p<0.05$)

Table 3. Female Mice: Body Weight Change

Group Number	Dose Level	Dose Route	Day of Termination	Initial Group Mean Body Weight (g) ± SD	Final Group Mean Body Weight (g) ± SD	Mean Body Weight Gain# (g) ± SD
1	0 mg/kg/day	Gavage	2	19.0±0.8	18.9±0.9	-0.1±0.5
2	50 mg/kg/day	Gavage	2	19.1±1.1	18.6±1.1	-0.6±0.7
3	100 mg/kg/day	Gavage	2	18.3±1.1	18.3±0.9	0.0±0.8
4	200 mg/kg/day	Gavage	2	18.5±1.0	17.5±1.5	-1.0±0.9
5	300 mg/kg/day	Gavage	2	18.7±1.1	17.8±1.3	-1.0±0.7
6	0 ppm	Feed	5	18.1±1.0	19.4±1.1	1.3±0.7
7	150 ppm	Feed	5	18.3±0.9	19.7±0.7	1.4±0.7
8	300 ppm	Feed	5	18.2±0.9	19.4±0.9	1.2±0.6
9	1250 ppm	Feed	5	18.5±0.8	19.2±0.9	0.8±0.5
10	2500 ppm	Feed	5	18.5±1.1	19.2±1.4	0.7±0.8
11	0 ppm	Feed	28	18.3±0.8	20.9±1.0	2.6±0.7
12	150 ppm	Feed	28	18.5±1.0	21.4±1.1	2.8±0.7
13	300 ppm	Feed	28	18.5±0.8	20.7±1.1	2.2±0.6
14	1250 ppm	Feed	28	18.3±1.2	20.5±1.3	2.1±0.8
15	2500 ppm	Feed	28	17.8±0.7	20.3±1.1	2.5±0.9

Abbreviation: SD- standard deviation

#Body weight gain determined from individual animal data

Food Consumption

Group mean food consumption (g/kg body weight/day) and 4-MEI consumed (mg of test article/kg body weight/day) for animals euthanized after 5 and 28 consecutive days of exposure are presented in Table 4 (males) and Table 5 (females). There was a statistically significant decrease in measured feed consumed by female mice provided 2500 ppm of 4-MEI in their diet for 28 days compared to

the control group. No statistical differences in measured food consumption were found for male mice. The values for mean 4-MEI consumed were calculated on the basis of nominal dose level values. Individual animal data are listed in Appendix III.

Table 4. Male Dose Group Mean Food Consumption

Group Number	Dose Level	Dose Route	Day of Termination	Mean Food Consumption (g/kg body weight/day) ± SD	Mean 4-Methylimidazole Consumed (mg/kg body weight/day) ± SD
6	0 ppm	Feed	5	224.1±40.0	NA
7	150 ppm	Feed	5	222.2±60.7	33.3±9.1
8	300 ppm	Feed	5	216.6±43.1	65.0±12.9
9	1250 ppm	Feed	5	235.7±31.8	294.6±39.7
10	2500 ppm	Feed	5	242.4±41.2	605.9±103.0
11	0 ppm	Feed	28	267.5±66.4	NA
12	150 ppm	Feed	28	260.3±40.3	39.0±6.0
13	300 ppm	Feed	28	277.2±47.4	83.2±14.2
14	1250 ppm	Feed	28	244.9±29.9	306.1±37.4
15	2500 ppm	Feed	28	258.5±36.4	646.2±90.9

Abbreviation: SD- standard deviation, NA – Not Applicable

Table 5. Female Dose Group Mean Food Consumption

Group Number	Dose Level	Dose Route	Day of Termination	Mean Food Consumption (g/kg body weight/day) ± SD	Mean 4-Methylimidazole Consumed (mg/kg body weight/day) ± SD
6	0 ppm	Feed	5	285.0±102.3	NA
7	150 ppm	Feed	5	334.9±47.4	50.2±7.1
8	300 ppm	Feed	5	295.5±91.4	88.6±27.4
9	1250 ppm	Feed	5	228.9±40.2	286.1±50.2

Group Number	Dose Level	Dose Route	Day of Termination	Mean Food Consumption (g/kg body weight/day) ± SD	Mean 4-Methylimidazole Consumed (mg/kg body weight/day) ± SD
10	2500 ppm	Feed	5	308.3±61.3	770.7±153.2
11	0 ppm	Feed	28	362.6±53.6	NA
12	150 ppm	Feed	28	330.9±34.5	49.6±5.2
13	300 ppm	Feed	28	367.6±45.9	110.3±13.8
14	1250 ppm	Feed	28	309.8±52.8	387.3±66.0
15	2500 ppm	Feed	28	299.7±39.8*	749.3±99.4

Abbreviation: SD- standard deviation, NA – Not Applicable

*****Statistically significant decrease compared to concurrent control (Dunnett's test $p<0.05$)

6.1 Necropsy Gross Observations

There were no abnormal gross observations noted in control animals or animals exposed to 4-MEI except for a cystic kidney (#044; 100 mg/kg 4-MEI), a mass on the liver (#075; 300 mg/kg 4-MEI), and a discoloured liver (#147; 2500 ppm 4-MEI for 28 days). Summary observations by group are listed in Appendix IV.

SUMMARY

There were no clinical observations noted during the study that would be associated with toxicity. There were no statistical changes in final body weight or body weight gain of animals exposed to 4-MEI compared to the control groups for 2 and 5 days; however, a statistically significant lower body weight gain in the male 150 and 300 ppm dose groups with a corresponding positive (decreasing) linear trend test was observed after 28 days of 4-MEI administration. There was a statistically significant decrease in measured feed consumed by female mice exposed to 2500 ppm of 4-MEI for 28 days compared to the control group. No other statistical differences in measured food consumption were found for male or female mice. There were minimal necropsy observations noted at any of the time points for either male or female mice administered 4-MEI at any tested dose level.

RNA was extracted from liver and lung from each animal, cDNA libraries were prepared and transcriptomes sequenced using next generation sequencing, and FASTQ files were delivered to ScitoVation for analysis.

REFERENCES

Institute of Laboratory Animal Resources. (2011). *Guide for the Care and Use of Laboratory Animals.* National Academy Press, Washington, DC.

NTP Technical Report on the Toxicology and Carcinogenesis studies of 4-Methylimidazole in F344/N Rats and $B6C3F_1$.

KEY PERSONNEL

Study Director:	Michael Streicker, B.S., LATG
Vice President, Research and Development:	Leslie Recio, Ph.D., DABT
Director of Toxicology:	Cheryl Hobbs, Ph.D.
Investigative Toxicology Study Coordinator:	Eileen Phillips, B.S., LAT
Formulations Manager:	Carol Swartz, Ph.D., DVM
Necropsy Coordinator:	John Pope, B.S.
Histology Coordinator:	John Pope, B.S.
Attending Veterinarian:	Alyssa McIntyre, D.V.M., DACLAM
Health and Safety Officer:	Michael Streicker, B.S., LATG

Appendix I: Clinical Observation Data

Clinical Observations - Clinical Signs with Site by Animal

50074.0001 - Short Term Toxicity and Genomics Study of 4-Methylimidazole in B6C3
F1 Male and Female Mice

Day numbers relative to Start Date

Group	Sex	Animal	Clinical Sign	Site	0	1	1 6-Hour Post-Dose
1	m	001	No Abnormalities Detected		X	X	X
		002	No Abnormalities Detected		X	X	X
		003	No Abnormalities Detected		X	X	X
		004	No Abnormalities Detected		X	X	X
		005	No Abnormalities Detected		X	X	X
		006	No Abnormalities Detected		X	X	X
		007	No Abnormalities Detected		X	X	X
		008	No Abnormalities Detected		X	X	X

Severity Codes: X = Present

Group 1 - Control 0 mg/kg   Group 2 - 4-MEI 50 mg/kg   Group 3 - 4-MEI 100 mg/kg
Group 4 - 4-MEI 200 mg/kg   Group 5 - 4-MEI 300 mg/kg

Clinical Observations - Clinical Signs with Site by Animal

50074.0001 - Short Term Toxicity and Genomics Study of 4-Methylimidazole in B6C3
F1 Male and Female Mice

Day numbers relative to Start Date

Group	Sex	Animal	Clinical Sign	Site	0	1	1 6-Hour Post-Dose
2	m	017	No Abnormalities Detected		X	X	X
		018	No Abnormalities Detected		X	X	X
		019	No Abnormalities Detected		X	X	X
		020	No Abnormalities Detected		X	X	X
		021	No Abnormalities Detected		X	X	X
		022	No Abnormalities Detected		X	X	X
		023	No Abnormalities Detected		X	X	X
		024	No Abnormalities Detected		X	X	X

Severity Codes: X = Present

Group 1 - Control 0 mg/kg   Group 2 - 4-MEI 50 mg/kg   Group 3 - 4-MEI 100 mg/kg
Group 4 - 4-MEI 200 mg/kg   Group 5 - 4-MEI 300 mg/kg

Clinical Observations - Clinical Signs with Site by Animal

50074.0001 - Short Term Toxicity and Genomics Study of 4-Methylimidazole in B6C3
F1 Male and Female Mice

Day numbers relative to Start Date

Group	Sex	Animal	Clinical Sign	Site	0	1	1<br>6-Hour<br>Post-Dose
3	m	033	No Abnormalities Detected		X	X	X
		034	No Abnormalities Detected		X	X	X
		035	No Abnormalities Detected		X	X	X
		036	No Abnormalities Detected		X	X	X
		037	No Abnormalities Detected		X	X	X
		038	No Abnormalities Detected		X	X	X
		039	No Abnormalities Detected		X	X	X
		040	No Abnormalities Detected		X	X	X

Severity Codes: X = Present

Group 1 - Control 0 mg/kg   Group 2 - 4-MEI 50 mg/kg   Group 3 - 4-MEI 100 mg/kg
Group 4 - 4-MEI 200 mg/kg   Group 5 - 4-MEI 300 mg/kg

Clinical Observations - Clinical Signs with Site by Animal

50074.0001 - Short Term Toxicity and Genomics Study of 4-Methylimidazole in B6C3 F1 Male and Female Mice

Day numbers relative to Start Date

Group	Sex	Animal	Clinical Sign	Site	0	1	1 6-Hour Post-Dose
4	m	049	No Abnormalities Detected		X	X	X
		050	No Abnormalities Detected		X	X	X
		051	No Abnormalities Detected		X	X	X
		052	No Abnormalities Detected		X	X	X
		053	No Abnormalities Detected		X	X	X
		054	No Abnormalities Detected		X	X	X
		055	No Abnormalities Detected		X	X	X
		056	No Abnormalities Detected		X	X	X

Severity Codes: X = Present

Group 1 - Control 0 mg/kg   Group 2 - 4-MEI 50 mg/kg   Group 3 - 4-MEI 100 mg/kg
Group 4 - 4-MEI 200 mg/kg   Group 5 - 4-MEI 300 mg/kg

Clinical Observations - Clinical Signs with Site by Animal

50074.0001 - Short Term Toxicity and Genomics Study of 4-Methylimidazole in B6C3 F1 Male and Female Mice

Day numbers relative to Start Date

Group	Sex	Animal	Clinical Sign	Site	0	1	1 6-Hour Post-Dose
5	m	065	No Abnormalities Detected		X	X	X
		066	No Abnormalities Detected		X	X	X
		067	No Abnormalities Detected		X	X	X
		068	No Abnormalities Detected		X	X	X
		069	No Abnormalities Detected		X	X	X
		070	No Abnormalities Detected		X	.	.
		071	No Abnormalities Detected		X	.	.
		072	No Abnormalities Detected		X	X	X

Severity Codes: X = Present

Group 1 - Control 0 mg/kg   Group 2 - 4-MEI 50 mg/kg   Group 3 - 4-MEI 100 mg/kg
Group 4 - 4-MEI 200 mg/kg   Group 5 - 4-MEI 300 mg/kg

Clinical Observations - Clinical Signs with Site by Animal

50074.0001 - Short Term Toxicity and Genomics Study of 4-Methylimidazole in B6C3
F1 Male and Female Mice

Day numbers relative to Start Date

Group	Sex	Animal	Clinical Sign	Site	0	1	1<br>6-Hour<br>Post-Dose
1	f	009	No Abnormalities Detected		X	X	X
		010	No Abnormalities Detected		X	X	X
		011	No Abnormalities Detected		X	X	X
		012	No Abnormalities Detected		X	X	X
		013	No Abnormalities Detected		X	X	X
		014	No Abnormalities Detected		X	X	X
		015	No Abnormalities Detected		X	X	X
		016	No Abnormalities Detected		X	X	X

Severity Codes: X = Present

Group 1 - Control 0 mg/kg   Group 2 - 4-MEI 50 mg/kg   Group 3 - 4-MEI 100 mg/kg
Group 4 - 4-MEI 200 mg/kg   Group 5 - 4-MEI 300 mg/kg

Clinical Observations - Clinical Signs with Site by Animal

50074.0001 - Short Term Toxicity and Genomics Study of 4-Methylimidazole in B6C3 F1 Male and Female Mice

Day numbers relative to Start Date

Group	Sex	Animal	Clinical Sign	Site	0	1	1 6-Hour Post-Dose
2	f	025	No Abnormalities Detected		X	X	X
		026	No Abnormalities Detected		X	X	X
		027	No Abnormalities Detected		X	X	X
		028	No Abnormalities Detected		X	X	X
		029	No Abnormalities Detected		X	X	X
		030	No Abnormalities Detected		X	X	X
		031	No Abnormalities Detected		X	X	X
		032	No Abnormalities Detected		X	X	X

Severity Codes: X = Present

Group 1 - Control 0 mg/kg   Group 2 - 4-MEI 50 mg/kg   Group 3 - 4-MEI 100 mg/kg
Group 4 - 4-MEI 200 mg/kg   Group 5 - 4-MEI 300 mg/kg

Clinical Observations - Clinical Signs with Site by Animal

50074.0001 - Short Term Toxicity and Genomics Study of 4-Methylimidazole in B6C3
F1 Male and Female Mice

Day numbers relative to Start Date

Group	Sex	Animal	Clinical Sign	Site	0	1	1 6-Hour Post-Dose
3	f	041	No Abnormalities Detected		X	X	X
		042	No Abnormalities Detected		X	X	X
		043	No Abnormalities Detected		X	X	X
		044	No Abnormalities Detected		X	X	X
		045	No Abnormalities Detected		X	X	X
		046	No Abnormalities Detected		X	X	X
		047	No Abnormalities Detected		X	X	X
		048	No Abnormalities Detected		X	X	X

Severity Codes: X = Present

Group 1 - Control 0 mg/kg   Group 2 - 4-MEI 50 mg/kg   Group 3 - 4-MEI 100 mg/kg
Group 4 - 4-MEI 200 mg/kg   Group 5 - 4-MEI 300 mg/kg

Clinical Observations - Clinical Signs with Site by Animal

50074.0001 - Short Term Toxicity and Genomics Study of 4-Methylimidazole in B6C3 F1 Male and Female Mice

Day numbers relative to Start Date

Group	Sex	Animal	Clinical Sign	Site	0	1	1<br>6-Hour<br>Post-Dose
4	f	057	No Abnormalities Detected		X	X	X
		058	No Abnormalities Detected		X	X	X
		059	No Abnormalities Detected		X	X	X
		060	No Abnormalities Detected		X	X	X
		061	No Abnormalities Detected		X	X	X
		062	No Abnormalities Detected		X	X	X
		063	No Abnormalities Detected		X	X	X
		064	No Abnormalities Detected		X	X	X

Severity Codes: X = Present

Group 1 - Control 0 mg/kg   Group 2 - 4-MEI 50 mg/kg   Group 3 - 4-MEI 100 mg/kg
Group 4 - 4-MEI 200 mg/kg   Group 5 - 4-MEI 300 mg/kg

Clinical Observations - Clinical Signs with Site by Animal

50074.0001 - Short Term Toxicity and Genomics Study of 4-Methylimidazole in B6C3
F1 Male and Female Mice

Day numbers relative to Start Date

Group	Sex	Animal	Clinical Sign	Site	0	1	1<br>6-Hour<br>Post-Dose
5	f	073	No Abnormalities Detected		X	X	X
		074	No Abnormalities Detected		X	X	X
		075	No Abnormalities Detected		X	X	X
		076	No Abnormalities Detected		X	X	X
		077	No Abnormalities Detected		X	X	X
		078	No Abnormalities Detected		X	X	X
		079	No Abnormalities Detected		X	X	X
		080	No Abnormalities Detected		X	X	X

Severity Codes: X = Present

Group 1 - Control 0 mg/kg   Group 2 - 4-MEI 50 mg/kg   Group 3 - 4-MEI 100 mg/kg
Group 4 - 4-MEI 200 mg/kg   Group 5 - 4-MEI 300 mg/kg

Clinical Observations - Clinical Signs with Site by Animal

50074.0001 - Short Term Toxicity and Genomics Study of 4-Methylimidazole in B6C3 F1 Male and Female Mice

Key Page

Clinical Observations - Clinical Signs with Site by Animal

50074.0001 - Short Term Toxicity and Genomics Study of 4-Methylimidazole in B6C3 F1 Male and Female Mice

Day numbers relative to Start Date

Group	Sex	Animal	Clinical Sign	Site	0	5
6	m	081	No Abnormalities Detected		X	X
		082	No Abnormalities Detected		X	X
		083	No Abnormalities Detected		X	X
		084	No Abnormalities Detected		X	X
		085	No Abnormalities Detected		X	X
		086	No Abnormalities Detected		X	X
		087	No Abnormalities Detected		X	X
		088	No Abnormalities Detected		X	X

Severity Codes: X = Present

Group 6 - Vehicle 0 ppm   Group 7 - 4-MEI 150 ppm   Group 8 - 4-MEI 300 ppm
Group 9 - 4-MEI 1250 ppm   Group 10 - 4-MEI 2500 ppm

Clinical Observations - Clinical Signs with Site by Animal

50074.0001 - Short Term Toxicity and Genomics Study of 4-Methylimidazole in B6C3
F1 Male and Female Mice

Day numbers relative to Start Date

Group	Sex	Animal	Clinical Sign	Site	0	5
7	m	097	No Abnormalities Detected		X	X
		098	No Abnormalities Detected		X	X
		099	No Abnormalities Detected		X	X
		100	No Abnormalities Detected		X	X
		101	No Abnormalities Detected		X	X
		102	No Abnormalities Detected		X	X
		103	No Abnormalities Detected		X	X
		104	No Abnormalities Detected		X	X

Severity Codes: X = Present

Group 6 - Vehicle 0 ppm   Group 7 - 4-MEI 150 ppm   Group 8 - 4-MEI 300 ppm
Group 9 - 4-MEI 1250 ppm   Group 10 - 4-MEI 2500 ppm

Clinical Observations - Clinical Signs with Site by Animal

50074.0001 - Short Term Toxicity and Genomics Study of 4-Methylimidazole in B6C3
F1 Male and Female Mice

Day numbers relative to Start Date

Group	Sex	Animal	Clinical Sign	Site	0	5
8	m	113	No Abnormalities Detected		X	X
		114	No Abnormalities Detected		X	X
		115	No Abnormalities Detected		X	X
		116	No Abnormalities Detected		X	X
		117	No Abnormalities Detected		X	X
		118	No Abnormalities Detected		X	X
		119	No Abnormalities Detected		X	X
		120	No Abnormalities Detected		X	X

Severity Codes: X = Present

Group 6 - Vehicle 0 ppm   Group 7 - 4-MEI 150 ppm   Group 8 - 4-MEI 300 ppm
Group 9 - 4-MEI 1250 ppm   Group 10 - 4-MEI 2500 ppm

Clinical Observations - Clinical Signs with Site by Animal

50074.0001 - Short Term Toxicity and Genomics Study of 4-Methylimidazole in B6C3
F1 Male and Female Mice

Day numbers relative to Start Date

Group	Sex	Animal	Clinical Sign	Site	0	5
9	m	129	No Abnormalities Detected		X	X
		130	No Abnormalities Detected		X	X
		131	No Abnormalities Detected		X	X
		132	No Abnormalities Detected		X	X
		133	No Abnormalities Detected		X	X
		134	No Abnormalities Detected		X	X
		135	No Abnormalities Detected		X	X
		136	No Abnormalities Detected		X	X

Severity Codes: X = Present

Group 6 - Vehicle 0 ppm   Group 7 - 4-MEI 150 ppm   Group 8 - 4-MEI 300 ppm
Group 9 - 4-MEI 1250 ppm   Group 10 - 4-MEI 2500 ppm

Clinical Observations - Clinical Signs with Site by Animal

50074.0001 - Short Term Toxicity and Genomics Study of 4-Methylimidazole in B6C3
F1 Male and Female Mice

Day numbers relative to Start Date

Group	Sex	Animal	Clinical Sign	Site	0	5
10	m	145	No Abnormalities Detected		X	X
		146	No Abnormalities Detected		X	X
		147	No Abnormalities Detected		X	X
		148	No Abnormalities Detected		X	X
		149	No Abnormalities Detected		X	X
		150	No Abnormalities Detected		X	X
		151	No Abnormalities Detected		X	X
		152	No Abnormalities Detected		X	X

Severity Codes: X = Present

Group 6 - Vehicle 0 ppm   Group 7 - 4-MEI 150 ppm   Group 8 - 4-MEI 300 ppm
Group 9 - 4-MEI 1250 ppm   Group 10 - 4-MEI 2500 ppm

Clinical Observations - Clinical Signs with Site by Animal

50074.0001 - Short Term Toxicity and Genomics Study of 4-Methylimidazole in B6C3 F1 Male and Female Mice

Day numbers relative to Start Date

Group	Sex	Animal	Clinical Sign	Site	0	5
6	f	089	No Abnormalities Detected		X	X
		090	No Abnormalities Detected		X	X
		091	No Abnormalities Detected		X	X
		092	No Abnormalities Detected		X	X
		093	No Abnormalities Detected		X	X
		094	No Abnormalities Detected		X	X
		095	No Abnormalities Detected		X	X
		096	No Abnormalities Detected		X	X

Severity Codes: X = Present

Group 6 - Vehicle 0 ppm Group 7 - 4-MEI 150 ppm Group 8 - 4-MEI 300 ppm
Group 9 - 4-MEI 1250 ppm Group 10 - 4-MEI 2500 ppm

Clinical Observations - Clinical Signs with Site by Animal

50074.0001 - Short Term Toxicity and Genomics Study of 4-Methylimidazole in B6C3
F1 Male and Female Mice

Day numbers relative to Start Date

Group	Sex	Animal	Clinical Sign	Site	0	5
7	f	105	No Abnormalities Detected		X	X
		106	No Abnormalities Detected		X	X
		107	No Abnormalities Detected		X	X
		108	No Abnormalities Detected		X	X
		109	No Abnormalities Detected		X	X
		110	No Abnormalities Detected		X	X
		111	No Abnormalities Detected		X	X
		112	No Abnormalities Detected		X	X

Severity Codes: X = Present

Group 6 - Vehicle 0 ppm   Group 7 - 4-MEI 150 ppm   Group 8 - 4-MEI 300 ppm
Group 9 - 4-MEI 1250 ppm   Group 10 - 4-MEI 2500 ppm

Clinical Observations - Clinical Signs with Site by Animal

50074.0001 - Short Term Toxicity and Genomics Study of 4-Methylimidazole in B6C3 F1 Male and Female Mice

Day numbers relative to Start Date

Group	Sex	Animal	Clinical Sign	Site	0	5
8	f	121	No Abnormalities Detected		X	X
		122	No Abnormalities Detected		X	X
		123	No Abnormalities Detected		X	X
		124	No Abnormalities Detected		X	X
		125	No Abnormalities Detected		X	X
		126	No Abnormalities Detected		X	X
		127	No Abnormalities Detected		X	X
		128	No Abnormalities Detected		X	X

Severity Codes: X = Present

Group 6 - Vehicle 0 ppm   Group 7 - 4-MEI 150 ppm   Group 8 - 4-MEI 300 ppm
Group 9 - 4-MEI 1250 ppm   Group 10 - 4-MEI 2500 ppm

Clinical Observations - Clinical Signs with Site by Animal

50074.0001 - Short Term Toxicity and Genomics Study of 4-Methylimidazole in B6C3
F1 Male and Female Mice

Day numbers relative to Start Date

Group	Sex	Animal	Clinical Sign	Site	0	5
9	f	137	No Abnormalities Detected		X	X
		138	No Abnormalities Detected		X	X
		139	No Abnormalities Detected		X	X
		140	No Abnormalities Detected		X	X
		141	No Abnormalities Detected		X	X
		142	No Abnormalities Detected		X	X
		143	No Abnormalities Detected		X	X
		144	No Abnormalities Detected		X	X

Severity Codes: X = Present

Group 6 - Vehicle 0 ppm   Group 7 - 4-MEI 150 ppm   Group 8 - 4-MEI 300 ppm
Group 9 - 4-MEI 1250 ppm   Group 10 - 4-MEI 2500 ppm

Clinical Observations - Clinical Signs with Site by Animal

50074.0001 - Short Term Toxicity and Genomics Study of 4-Methylimidazole in B6C3
F1 Male and Female Mice

Day numbers relative to Start Date

Group	Sex	Animal	Clinical Sign	Site	0	5
10	f	153	No Abnormalities Detected		X	X
		154	No Abnormalities Detected		X	X
		155	No Abnormalities Detected		X	X
		156	No Abnormalities Detected		X	X
		157	No Abnormalities Detected		X	X
		158	No Abnormalities Detected		X	X
		159	No Abnormalities Detected		X	X
		160	No Abnormalities Detected		X	X

Severity Codes: X = Present

Group 6 - Vehicle 0 ppm   Group 7 - 4-MEI 150 ppm   Group 8 - 4-MEI 300 ppm
Group 9 - 4-MEI 1250 ppm   Group 10 - 4-MEI 2500 ppm

Clinical Observations - Clinical Signs with Site by Animal

50074.0001 - Short Term Toxicity and Genomics Study of 4-Methylimidazole in B6C3
F1 Male and Female Mice

Key Page

Clinical Observations - Clinical Signs with Site by Animal

50074.0001 - Short Term Toxicity and Genomics Study of 4-Methylimidazole in B6C3 F1 Male and Female Mice

Day numbers relative to Start Date

Group	Sex	Animal	Clinical Sign	Site	0	7	14	21	28
11	m	161	No Abnormalities Detected		X	.	.	.	.
			Alopecia	Abdomen	.	1	1	1	1
		162	No Abnormalities Detected		X	X	X	X	X
		163	No Abnormalities Detected		X	X	X	X	X
		164	No Abnormalities Detected		X	X	X	X	X
		165	No Abnormalities Detected		X	X	X	X	X
		166	No Abnormalities Detected		X	X	X	X	X
		167	No Abnormalities Detected		X	X	X	X	X
		168	No Abnormalities Detected		X	X	X	X	X

Severity Codes: X = Present; 1 = Mild

Group 11 - Vehicle 0 ppm   Group 12 - 4-MEI 150 ppm   Group 13 - 4-MEI 300 ppm
Group 14 - 4-MEI 1250 ppm   Group 15 - 4-MEI 2500 ppm

Clinical Observations - Clinical Signs with Site by Animal

50074.0001 - Short Term Toxicity and Genomics Study of 4-Methylimidazole in B6C3 F1 Male and Female Mice

Day numbers relative to Start Date

Group	Sex	Animal	Clinical Sign	Site	0	7	14	21	28
12	m	177	No Abnormalities Detected		X	X	X	X	X
		178	No Abnormalities Detected		X	X	X	X	X
		179	No Abnormalities Detected		X	X	X	X	X
		180	No Abnormalities Detected		X	X	X	X	X
		181	No Abnormalities Detected		X	X	X	X	X
		182	No Abnormalities Detected		X	X	X	X	X
		183	No Abnormalities Detected		X	X	X	X	X
		184	No Abnormalities Detected		X	X	X	X	X

Severity Codes: X = Present; 1 = Mild

Group 11 - Vehicle 0 ppm   Group 12 - 4-MEI 150 ppm   Group 13 - 4-MEI 300 ppm
Group 14 - 4-MEI 1250 ppm   Group 15 - 4-MEI 2500 ppm

Clinical Observations - Clinical Signs with Site by Animal

50074.0001 - Short Term Toxicity and Genomics Study of 4-Methylimidazole in B6C3 F1 Male and Female Mice

Day numbers relative to Start Date

Group	Sex	Animal	Clinical Sign	Site	0	7	14	21	28
13	m	193	No Abnormalities Detected		X	X	X	X	X
		194	No Abnormalities Detected		X	X	X	X	X
		195	No Abnormalities Detected		X	X	X	X	X
		196	No Abnormalities Detected		X	X	X	X	X
		197	No Abnormalities Detected		X	X	X	X	X
		198	No Abnormalities Detected		X	X	X	X	X
		199	No Abnormalities Detected		X	X	X	X	X
		200	No Abnormalities Detected		X	X	X	X	X

Severity Codes: X = Present; 1 = Mild

Group 11 - Vehicle 0 ppm Group 12 - 4-MEI 150 ppm Group 13 - 4-MEI 300 ppm
Group 14 - 4-MEI 1250 ppm Group 15 - 4-MEI 2500 ppm

Clinical Observations - Clinical Signs with Site by Animal

50074.0001 - Short Term Toxicity and Genomics Study of 4-Methylimidazole in B6C3
F1 Male and Female Mice

Day numbers relative to Start Date

Group	Sex	Animal	Clinical Sign	Site	0	7	14	21	28
14	m	209	No Abnormalities Detected		X	X	X	X	X
		210	No Abnormalities Detected		X	X	X	X	X
		211	No Abnormalities Detected		X	X	X	X	X
		212	No Abnormalities Detected		X	X	X	X	X
		213	No Abnormalities Detected		X	X	X	X	X
		214	No Abnormalities Detected		X	X	X	X	X
		215	No Abnormalities Detected		X	X	X	X	X
		216	No Abnormalities Detected		X	X	X	X	X

Severity Codes: X = Present; 1 = Mild

Group 11 - Vehicle 0 ppm   Group 12 - 4-MEI 150 ppm   Group 13 - 4-MEI 300 ppm
Group 14 - 4-MEI 1250 ppm   Group 15 - 4-MEI 2500 ppm

Clinical Observations - Clinical Signs with Site by Animal

50074.0001 - Short Term Toxicity and Genomics Study of 4-Methylimidazole in B6C3
F1 Male and Female Mice

Day numbers relative to Start Date

Group	Sex	Animal	Clinical Sign	Site	0	7	14	21	28
15	m	225	No Abnormalities Detected		X	X	X	X	X
		226	No Abnormalities Detected		X	X	X	X	X
		227	No Abnormalities Detected		X	X	X	X	X
		228	No Abnormalities Detected		X	X	X	X	X
		229	No Abnormalities Detected		X	X	X	X	X
		230	No Abnormalities Detected		X	X	X	X	X
		231	No Abnormalities Detected		X	X	X	X	X
		232	No Abnormalities Detected		X	X	X	X	X

Severity Codes: X = Present; 1 = Mild

Group 11 - Vehicle 0 ppm   Group 12 - 4-MEI 150 ppm   Group 13 - 4-MEI 300 ppm
Group 14 - 4-MEI 1250 ppm   Group 15 - 4-MEI 2500 ppm

Clinical Observations - Clinical Signs with Site by Animal

50074.0001 - Short Term Toxicity and Genomics Study of 4-Methylimidazole in B6C3 F1 Male and Female Mice

Day numbers relative to Start Date

Group	Sex	Animal	Clinical Sign	Site	0	7	14	21	28
11	f	169	No Abnormalities Detected		X	X	X	X	X
		170	No Abnormalities Detected		X	X	X	X	X
		171	No Abnormalities Detected		X	X	X	X	X
		172	No Abnormalities Detected		X	X	X	X	X
		173	No Abnormalities Detected		X	X	X	X	X
		174	No Abnormalities Detected		X	X	X	X	X
		175	No Abnormalities Detected		X	X	X	X	X
		176	No Abnormalities Detected		X	X	X	X	X

Severity Codes: X = Present; 1 = Mild

Group 11 - Vehicle 0 ppm Group 12 - 4-MEI 150 ppm Group 13 - 4-MEI 300 ppm
Group 14 - 4-MEI 1250 ppm Group 15 - 4-MEI 2500 ppm

Clinical Observations - Clinical Signs with Site by Animal

50074.0001 - Short Term Toxicity and Genomics Study of 4-Methylimidazole in B6C3 F1 Male and Female Mice

Day numbers relative to Start Date

Group	Sex	Animal	Clinical Sign	Site	0	7	14	21	28
12	f	185	No Abnormalities Detected		X	X	X	X	X
		186	No Abnormalities Detected		X	X	X	X	X
		187	No Abnormalities Detected		X	X	X	X	X
		188	No Abnormalities Detected		X	X	X	X	X
		189	No Abnormalities Detected		X	X	X	X	X
		190	No Abnormalities Detected		X	X	X	X	X
		191	No Abnormalities Detected		X	X	X	X	X
		192	No Abnormalities Detected		X	X	X	X	X

Severity Codes: X = Present; 1 = Mild

Group 11 - Vehicle 0 ppm   Group 12 - 4-MEI 150 ppm   Group 13 - 4-MEI 300 ppm
Group 14 - 4-MEI 1250 ppm   Group 15 - 4-MEI 2500 ppm

Clinical Observations - Clinical Signs with Site by Animal

50074.0001 - Short Term Toxicity and Genomics Study of 4-Methylimidazole in B6C3 F1 Male and Female Mice

Day numbers relative to Start Date

Group	Sex	Animal	Clinical Sign	Site	0	7	14	21	28
13	f	201	No Abnormalities Detected		X	X	X	X	X
		202	No Abnormalities Detected		X	X	X	X	X
		203	No Abnormalities Detected		X	X	X	X	X
		204	No Abnormalities Detected		X	X	X	X	X
		205	No Abnormalities Detected		X	X	X	X	X
		206	No Abnormalities Detected		X	X	X	X	X
		207	No Abnormalities Detected		X	X	X	X	X
		208	No Abnormalities Detected		X	X	X	X	X

Severity Codes: X = Present; 1 = Mild

Group 11 - Vehicle 0 ppm   Group 12 - 4-MEI 150 ppm   Group 13 - 4-MEI 300 ppm
Group 14 - 4-MEI 1250 ppm   Group 15 - 4-MEI 2500 ppm

Clinical Observations - Clinical Signs with Site by Animal

50074.0001 - Short Term Toxicity and Genomics Study of 4-Methylimidazole in B6C3 F1 Male and Female Mice

Day numbers relative to Start Date

Group	Sex	Animal	Clinical Sign	Site	0	7	14	21	28
14	f	217	No Abnormalities Detected		X	X	X	X	X
		218	No Abnormalities Detected		X	X	X	X	X
		219	No Abnormalities Detected		X	X	X	X	X
		220	No Abnormalities Detected		X	X	X	X	X
		221	No Abnormalities Detected		X	X	X	X	X
		222	No Abnormalities Detected		X	X	X	X	X
		223	No Abnormalities Detected		X	X	X	X	X
		224	No Abnormalities Detected		X	X	X	X	X

Severity Codes: X = Present; 1 = Mild

Group 11 - Vehicle 0 ppm Group 12 - 4-MEI 150 ppm Group 13 - 4-MEI 300 ppm
Group 14 - 4-MEI 1250 ppm Group 15 - 4-MEI 2500 ppm

Clinical Observations - Clinical Signs with Site by Animal

50074.0001 - Short Term Toxicity and Genomics Study of 4-Methylimidazole in B6C3 F1 Male and Female Mice

Day numbers relative to Start Date

Group	Sex	Animal	Clinical Sign	Site	0	7	14	21	28
15	f	233	No Abnormalities Detected		X	X	X	X	X
		234	No Abnormalities Detected		X	X	X	X	X
		235	No Abnormalities Detected		X	X	X	X	X
		236	No Abnormalities Detected		X	X	X	X	X
		237	No Abnormalities Detected		X	X	X	X	X
		238	No Abnormalities Detected		X	X	X	X	X
		239	No Abnormalities Detected		X	X	X	X	X
		240	No Abnormalities Detected		X	X	X	X	X

Severity Codes: X = Present; 1 = Mild

Group 11 - Vehicle 0 ppm   Group 12 - 4-MEI 150 ppm   Group 13 - 4-MEI 300 ppm
Group 14 - 4-MEI 1250 ppm   Group 15 - 4-MEI 2500 ppm

Clinical Observations - Clinical Signs with Site by Animal

50074.0001 - Short Term Toxicity and Genomics Study of 4-Methylimidazole in B6C3 F1 Male and Female Mice

Key Page

Appendix II: Body Weight Data

Generalised Results - Animals by Mixed Parameter / Time

Sex: Male Day(s) Relative to Start Date

Control

0 mg/kg

	Body Weight (g)	Body Weight (g)	Terminal Body Wt. (g)
	0	1	1
001	22.4	22.9	22.9
002	23.2	23.7	23.7
003	24.0	24.1	24.1
004	21.0	23.0	23.0
005	23.4	24.5	24.5
006	25.6	26.3	26.3
007	23.6	24.5	24.5
008	24.9	27.1	27.1
Mean	23.51	24.51	24.51
SD	1.42	1.49	1.49
N	8	8	8

Generalised Results - Animals by Mixed Parameter / Time

Sex: Male Day(s) Relative to Start Date

4-MEI

50 mg/kg

	Body Weight (g)	Body Weight (g)	Terminal Body Wt. (g)
	0	1	1
017	23.6	24.0	24.0
018	26.6	26.0	26.0
019	23.4	22.6	22.6
020	23.5	25.3	25.3
021	23.8	25.5	25.5
022	20.5	24.4	24.4
023	27.6	27.1	27.1
024	25.0	26.0	26.0
Mean	24.25	25.11	25.11
SD	2.18	1.40	1.40
N	8	8	8

Generalised Results - Animals by Mixed Parameter / Time

Sex: Male Day(s) Relative to Start Date

4-MEI 100 mg/kg	Body Weight (g) 0	Body Weight (g) 1	Terminal Body Wt. (g) 1
033	23.9	22.9	22.9
034	26.1	26.0	26.0
035	23.7	23.7	23.7
036	23.6	24.4	24.4
037	25.8	26.9	26.9
038	22.0	22.9	22.9
039	21.9	22.9	22.9
040	26.2	26.5	26.5
Mean	24.15	24.53	24.53
SD	1.73	1.70	1.70
N	8	8	8

Generalised Results - Animals by Mixed Parameter / Time

Sex: Male Day(s) Relative to Start Date

4-MEI

200 mg/kg

	Body Weight (g) 0	Body Weight (g) 1	Terminal Body Wt. (g) 1
049	23.3	23.6	23.6
050	22.3	22.4	22.4
051	24.2	22.8	22.8
052	22.9	22.5	22.5
053	25.6	24.7	24.7
054	20.7	22.0	22.0
055	23.0	23.1	23.1
056	24.8	26.0	26.0
Mean	23.35	23.39	23.39
SD	1.53	1.35	1.35
N	8	8	8

Generalised Results - Animals by Mixed Parameter / Time

Sex: Male Day(s) Relative to Start Date

4-MEI 300 mg/kg	Body Weight (g)	Body Weight (g)	Terminal Body Wt. (g)
	0	1	1
065	23.7	22.8	22.8
066	21.1	19.1	19.1
067	27.3	26.0	26.0
068	24.8	23.8	23.8
069	22.6	23.9	23.9
072	23.1	26.1	26.1
Mean	23.77	23.62	23.62
SD	2.12	2.57	2.57
N	6	6	6

Generalised Results - Animals by Mixed Parameter / Time

Sex: Female Day(s) Relative to Start Date

Control

0 mg/kg

	Body Weight (g)	Body Weight (g)	Terminal Body Wt. (g)
	0	1	1
009	19.4	19.7	19.7
010	19.8	20.5	20.5
011	18.5	18.1	18.1
012	19.8	19.3	19.3
013	18.3	17.7	17.7
014	17.9	18.2	18.2
015	18.6	18.8	18.8
016	19.6	19.1	19.1
Mean	18.99	18.93	18.93
SD	0.75	0.92	0.92
N	8	8	8

Generalised Results - Animals by Mixed Parameter / Time

Sex: Female Day(s) Relative to Start Date

4-MEI 50 mg/kg	Body Weight (g)	Body Weight (g)	Terminal Body Wt. (g)
	0	1	1
025	19.7	19.0	19.0
026	20.1	18.8	18.8
027	18.5	18.1	18.1
028	17.0	16.3	16.3
029	19.4	19.0	19.0
030	19.9	18.3	18.3
031	20.3	20.1	20.1
032	18.2	18.8	18.8
Mean	19.14	18.55	18.55
SD	1.14	1.09	1.09
N	8	8	8

Generalised Results - Animals by Mixed Parameter / Time

Sex: Female Day(s) Relative to Start Date

4-MEI

100 mg/kg

	Body Weight (g)	Body Weight (g)	Terminal Body Wt. (g)
	0	1	1
041	17.8	17.7	17.7
042	18.7	19.6	19.6
043	17.3	17.9	17.9
044	17.1	16.6	16.6
045	19.0	19.1	19.1
046	19.1	19.0	19.0
047	20.1	18.5	18.5
048	17.5	18.1	18.1
Mean	18.33	18.31	18.31
SD	1.06	0.95	0.95
N	8	8	8

Generalised Results - Animals by Mixed Parameter / Time

Sex: Female Day(s) Relative to Start Date

4-MEI 200 mg/kg	Body Weight (g) 0	Body Weight (g) 1	Terminal Body Wt. (g) 1
057	20.1	20.2	20.2
058	17.8	17.1	17.1
059	17.3	17.1	17.1
060	19.4	18.1	18.1
061	19.2	18.0	18.0
062	17.9	17.0	17.0
063	18.5	17.9	17.9
064	17.5	14.7	14.7
Mean	18.46	17.51	17.51
SD	1.01	1.53	1.53
N	8	8	8

Generalised Results - Animals by Mixed Parameter / Time

Sex: Female Day(s) Relative to Start Date

4-MEI 300 mg/kg	Body Weight (g)	Body Weight (g)	Terminal Body Wt. (g)
	0	1	1
073	19.0	17.5	17.5
074	17.7	16.2	16.2
075	19.0	17.7	17.7
076	18.6	18.0	18.0
077	17.8	16.2	16.2
078	17.6	17.9	17.9
079	21.1	20.2	20.2
080	18.9	18.3	18.3
Mean	18.71	17.75	17.75
SD	1.13	1.27	1.27
N	8	8	8

Generalised Results - Animals by Mixed Parameter / Time

Key Page

Measurement Descriptions

Headings Used	Description
Body Weight	Bodyweight
Terminal Body Wt.	Terminal Bodyweight

Unit Descriptions

Headings Used	Description
g	g

Time-Points/Ranges

Measurement	From	To	Report As
Terminal Body Wt.	-9,999	9,999	1

Measurement/Statistics

Measurement	Descriptive
Body Weight	Mean
	Standard Deviation
	Count (N)
Terminal Body Wt.	Mean
	Standard Deviation
	Count (N)

Group Information

Short Name	Long Name	Report Headings 1-4	
1	Group 1	Control	0 mg/kg

Generalised Results - Animals by Mixed Parameter / Time

Key Page

Group Information (Continued)

Short Name	Long Name	Report Headings 1-4	
2	Group 2	4-MEI	50 mg/kg
3	Group 3	4-MEI	100 mg/kg
4	Group 4	4-MEI	200 mg/kg
5	Group 5	4-MEI	300 mg/kg

Generalised Results - Animals by Mixed Parameter / Time

Sex: Male Day(s) Relative to Start Date

4-MEI

150 ppm

	Body Weight (g)	Body Weight (g)
	0	5
097	23.6	26.6
098	23.2	23.8
099	27.1	26.4
100	20.2	21.2
101	25.1	25.7
102	23.1	24.3
103	23.6	24.1
104	22.5	22.6
Mean	23.55	24.34
SD	1.99	1.87
N	8	8

Generalised Results - Animals by Mixed Parameter / Time

Sex: Male Day(s) Relative to Start Date

4-MEI

300 ppm

	Body Weight (g)	Body Weight (g)
	0	5
113	24.7	25.7
114	23.0	24.7
115	21.8	24.1
116	26.3	25.5
117	22.0	23.3
118	25.8	26.0
119	22.2	22.3
120	26.1	26.1
Mean	23.99	24.71
SD	1.95	1.38
N	8	8

Generalised Results - Animals by Mixed Parameter / Time

Sex: Male Day(s) Relative to Start Date

4-MEI 1250 ppm	Body Weight (g) 0	Body Weight (g) 5
129	22.5	23.8
130	25.1	26.4
131	24.1	24.7
132	21.9	24.3
133	23.8	25.9
134	26.2	27.6
135	20.9	22.7
136	22.2	23.9
Mean	23.34	24.91
SD	1.78	1.60
N	8	8

Generalised Results - Animals by Mixed Parameter / Time

Sex: Male Day(s) Relative to Start Date

4-MEI 2500 ppm	Body Weight (g) 0	Body Weight (g) 5
145	23.8	24.0
146	24.7	25.5
147	20.0	23.9
148	24.6	25.1
149	24.6	26.2
150	22.3	23.2
151	23.3	24.4
152	22.3	23.0
Mean	23.20	24.41
SD	1.62	1.12
N	8	8

Generalised Results - Animals by Mixed Parameter / Time

Sex: Female Day(s) Relative to Start Date

Vehicle 0 ppm	Body Weight (g) 0	Body Weight (g) 5
089	17.8	19.9
090	18.8	20.5
091	18.6	18.9
092	19.3	21.1
093	18.6	19.0
094	18.5	19.1
095	17.0	18.3
096	16.3	18.1
Mean	18.11	19.36
SD	1.01	1.05
N	8	8

Generalised Results - Animals by Mixed Parameter / Time

Sex: Female Day(s) Relative to Start Date

4-MEI

150 ppm

	Body Weight (g)	Body Weight (g)
	0	5
105	17.3	18.7
106	19.1	20.4
107	19.0	20.4
108	17.3	19.9
109	18.9	20.2
110	17.0	19.0
111	18.4	19.3
112	19.0	19.4
Mean	18.25	19.66
SD	0.90	0.65
N	8	8

Generalised Results - Animals by Mixed Parameter / Time

Sex: Female Day(s) Relative to Start Date

4-MEI

300 ppm

	Body Weight (g)	Body Weight (g)
	0	5
121	18.4	19.0
122	17.4	19.2
123	17.2	17.8
124	17.6	19.2
125	17.3	19.4
126	19.2	20.1
127	19.4	20.8
128	18.8	19.7
Mean	18.16	19.40
SD	0.90	0.87
N	8	8

Generalised Results - Animals by Mixed Parameter / Time

Sex: Female Day(s) Relative to Start Date

4-MEI 1250 ppm	Body Weight (g) 0	Body Weight (g) 5
137	18.3	19.8
138	18.0	19.3
139	17.8	18.5
140	20.1	21.1
141	18.6	19.4
142	17.9	18.2
143	18.1	18.8
144	18.9	18.7
Mean	18.46	19.23
SD	0.76	0.92
N	8	8

Generalised Results - Animals by Mixed Parameter / Time

Sex: Female Day(s) Relative to Start Date

4-MEI 2500 ppm	Body Weight (g) 0	Body Weight (g) 5
153	18.6	20.8
154	17.6	18.5
155	18.7	19.8
156	20.3	20.8
157	19.7	19.9
158	17.3	16.9
159	18.5	18.5
160	17.5	18.3
Mean	18.53	19.19
SD	1.07	1.37
N	8	8

Generalised Results - Animals by Mixed Parameter / Time

Key Page

Measurement Descriptions

Headings Used	Description
Body Weight	Bodyweight

Unit Descriptions

Headings Used	Description
g	g

Measurement/Statistics

Measurement	Descriptive
Body Weight	Mean
	Standard Deviation
	Count (N)

Group Information

Short Name	Long Name	Report Headings 1-4	
6	Group 6	Vehicle	0 ppm
7	Group 7	4-MEI	150 ppm
8	Group 8	4-MEI	300 ppm
9	Group 9	4-MEI	1250 ppm
10	Group 10	4-MEI	2500 ppm

Generalised Results - Animals by Mixed Parameter / Time

Sex: Male Day(s) Relative to Start Date

4-MEI 150 ppm	Body Weight (g) 0	Body Weight (g) 7	Body Weight (g) 14	Body Weight (g) 21	Body Weight (g) 28
177	25.6	28.4	28.2	27.8	28.2
178	21.4	22.1	21.8	22.0	22.0
179	23.9	25.0	25.1	25.1	26.1
180	26.4	26.6	26.7	26.0	26.9
181	23.8	24.9	24.4	24.2	24.0
182	22.8	24.8	25.1	25.1	25.5
183	26.4	25.9	27.5	25.8	26.8
184	22.3	24.0	23.7	23.9	24.1
Mean	24.08	25.21	25.31	24.99	25.45
SD	1.90	1.85	2.10	1.70	1.99
N	8	8	8	8	8

Generalised Results - Animals by Mixed Parameter / Time

Sex: Male Day(s) Relative to Start Date

4-MEI 300 ppm	Body Weight (g) 0	Body Weight (g) 7	Body Weight (g) 14	Body Weight (g) 21	Body Weight (g) 28
193	23.2	23.7	23.6	23.9	23.5
194	26.8	27.6	27.8	22.0	29.1
195	24.8	25.2	25.7	25.3	25.9
196	22.5	23.1	22.6	22.7	23.1
197	25.2	25.7	25.5	25.1	24.9
198	25.1	25.2	25.4	25.3	25.2
199	21.2	23.1	23.1	23.9	24.1
200	23.4	24.2	24.2	24.1	25.5
Mean	24.03	24.73	24.74	24.04	25.16
SD	1.78	1.52	1.70	1.21	1.87
N	8	8	8	8	8

Generalised Results - Animals by Mixed Parameter / Time

Sex: Male Day(s) Relative to Start Date

4-MEI 1250 ppm	Body Weight (g) 0	Body Weight (g) 7	Body Weight (g) 14	Body Weight (g) 21	Body Weight (g) 28
209	22.8	23.6	23.3	23.3	23.9
210	24.2	26.8	26.9	26.9	27.4
211	23.7	25.0	24.5	24.2	24.3
212	23.5	25.5	25.1	24.4	24.9
213	26.4	28.9	28.6	28.1	28.8
214	20.8	23.1	23.6	23.1	23.5
215	23.4	25.8	25.7	24.8	25.5
216	21.9	23.1	22.9	22.9	23.8
Mean	23.34	25.23	25.08	24.71	25.26
SD	1.65	2.00	1.95	1.87	1.90
N	8	8	8	8	8

Generalised Results - Animals by Mixed Parameter / Time

Sex: Male Day(s) Relative to Start Date

4-MEI 2500 ppm	Body Weight (g) 0	Body Weight (g) 7	Body Weight (g) 14	Body Weight (g) 21	Body Weight (g) 28
225	22.6	25.2	25.5	25.1	25.7
226	22.4	23.6	23.9	24.2	25.1
227	23.3	26.1	26.0	25.6	26.3
228	24.4	26.0	26.6	25.8	27.2
229	21.5	24.3	24.5	24.8	24.4
230	23.4	25.4	24.6	24.8	25.5
231	23.1	25.0	24.9	25.3	25.1
232	20.7	25.0	25.3	24.9	25.4
Mean	22.68	25.08	25.16	25.06	25.59
SD	1.16	0.83	0.87	0.51	0.85
N	8	8	8	8	8

Generalised Results - Animals by Mixed Parameter / Time

Sex: Female Day(s) Relative to Start Date

Vehicle

0 ppm

	Body Weight (g)	Body Weight (g)	Body Weight (g)	Body Weight (g)	Body Weight (g)
	0	7	14	21	28
169	17.6	18.8	19.1	20.3	20.4
170	17.8	19.9	20.5	20.3	20.9
171	18.6	21.1	21.0	20.7	21.1
172	18.4	21.2	21.3	20.5	22.6
173	17.3	18.1	18.8	19.2	19.6
174	18.7	20.2	20.0	20.5	20.9
175	18.2	19.5	20.0	20.6	20.1
176	19.7	21.0	21.7	21.3	21.8
Mean	18.29	19.98	20.30	20.43	20.93
SD	0.75	1.13	1.03	0.59	0.95
N	8	8	8	8	8

Generalised Results - Animals by Mixed Parameter / Time

Sex: Female Day(s) Relative to Start Date

4-MEI 150 ppm	Body Weight (g) 0	Body Weight (g) 7	Body Weight (g) 14	Body Weight (g) 21	Body Weight (g) 28
185	19.5	21.0	22.4	22.7	22.8
186	17.4	18.4	20.2	19.9	20.5
187	19.1	20.1	20.8	20.0	20.7
188	18.7	20.5	21.4	20.7	22.7
189	17.8	19.7	19.4	20.1	20.6
190	20.2	20.2	21.3	22.6	22.6
191	17.7	19.4	19.7	19.2	20.7
192	17.9	19.0	20.0	20.1	20.2
Mean	18.54	19.79	20.65	20.66	21.35
SD	1.00	0.84	1.01	1.29	1.13
N	8	8	8	8	8

Generalised Results - Animals by Mixed Parameter / Time

Sex: Female Day(s) Relative to Start Date

4-MEI

300 ppm

	Body Weight (g) 0	Body Weight (g) 7	Body Weight (g) 14	Body Weight (g) 21	Body Weight (g) 28
201	17.6	19.7	20.5	19.6	19.8
202	20.0	20.7	20.7	21.2	22.6
203	18.5	21.4	21.4	21.0	21.2
204	18.5	20.4	21.1	20.8	21.4
205	19.0	21.0	22.0	21.1	21.0
206	18.2	19.6	20.5	19.5	19.5
207	17.5	19.3	19.7	20.5	19.6
208	18.5	20.2	20.5	20.4	20.1
Mean	18.48	20.29	20.80	20.51	20.65
SD	0.79	0.73	0.69	0.66	1.08
N	8	8	8	8	8

Generalised Results - Animals by Mixed Parameter / Time

Sex: Female Day(s) Relative to Start Date

4-MEI

1250 ppm

	Body Weight (g)	Body Weight (g)	Body Weight (g)	Body Weight (g)	Body Weight (g)
	0	7	14	21	28
217	18.0	20.2	19.7	19.4	20.5
218	19.6	20.5	21.2	20.1	20.6
219	17.5	18.6	18.5	18.6	18.7
220	16.4	18.1	18.6	18.8	18.9
221	18.3	19.4	20.1	19.6	20.2
222	19.9	21.8	22.1	22.1	22.7
223	19.0	20.5	20.7	20.5	20.7
224	17.9	21.1	21.1	21.8	21.3
Mean	18.33	20.03	20.25	20.11	20.45
SD	1.15	1.25	1.27	1.29	1.28
N	8	8	8	8	8

Generalised Results - Animals by Mixed Parameter / Time

Sex: Female Day(s) Relative to Start Date

4-MEI 2500 ppm	Body Weight (g) 0	Body Weight (g) 7	Body Weight (g) 14	Body Weight (g) 21	Body Weight (g) 28
233	17.7	18.5	18.6	18.3	18.9
234	18.4	21.1	18.4	20.9	21.7
235	18.6	19.3	21.2	19.8	20.6
236	18.0	19.4	19.9	19.8	20.9
237	16.9	18.0	19.4	18.5	18.5
238	17.0	19.2	20.0	19.4	20.3
239	17.6	19.9	20.6	20.3	21.0
240	18.5	19.9	19.7	20.2	20.5
Mean	17.84	19.41	19.73	19.65	20.30
SD	0.66	0.94	0.94	0.89	1.08
N	8	8	8	8	8

Generalised Results - Animals by Mixed Parameter / Time

Key Page

Measurement Descriptions

Headings Used	Description
Body Weight	Bodyweight

Unit Descriptions

Headings Used	Description
g	g

Measurement/Statistics

Measurement	Descriptive
Body Weight	Mean<br>Standard Deviation<br>Count (N)

Group Information

Short Name	Long Name	Report Headings 1-4	
11	Group 11	Vehicle	0 ppm
12	Group 12	4-MEI	150 ppm
13	Group 13	4-MEI	300 ppm
14	Group 14	4-MEI	1250 ppm
15	Group 15	4-MEI	2500 ppm

Appendix III: Food Consumption Data

Day 5

Group: 6 (0 ppm) Males		
Animal No.	g/kg body weight/ Day	mg test article/kg body weight/Day
81	259.8	0.0
82	240.3	0.0
83	291.9	0.0
84	192.9	0.0
85	177.8	0.0
86	239.3	0.0
87	199.6	0.0
88	191.0	0.0
**Mean**	**224.1**	**0.0**
**SD**	**40.0**	**0.0**
**Count**	**8**	**8**

Group: 7 (150 ppm) Males		
Animal No.	g/kg body weight/ Day	mg test article/kg body weight/Day
97	303.6	45.5
98	174.5	26.2
99	211.6	31.7
100	115.9	17.4
101	216.5	32.5
102	240.5	36.1
103	221.4	33.2
104	293.6	44.0
**Mean**	**222.2**	**33.3**
**SD**	**60.7**	**9.1**
**Count**	**8**	**8**

Group: 8 (300 ppm) Males		
Animal No.	g/kg body weight/ Day	mg test article/kg body weight/Day
113	237.3	71.2
114	209.6	62.9
115	307.6	92.3
116	225.5	67.7
117	211.9	63.6
118	181.5	54.5
119	169.9	51.0
120	189.3	56.8
**Mean**	**216.6**	**65.0**
**SD**	**43.1**	**12.9**
**Count**	**8**	**8**

Group: 9 (1250 ppm) Males		
Animal No.	g/kg body weight/ Day	mg test article/kg body weight/Day
129	236.7	295.9
130	208.9	261.1
131	185.3	231.6
132	286.6	358.3
133	264.8	331.0
134	249.8	312.3
135	228.4	285.5
136	224.7	280.9
**Mean**	**235.7**	**294.6**
**SD**	**31.8**	**39.7**
**Count**	**8**	**8**

Group: 10 (2500 ppm) Males		
Animal No.	g/kg body weight/ Day	mg test article/kg body weight/Day
145	190.8	477.0
146	220.7	551.8
147	229.8	574.5
148	215.7	539.3
149	280.3	700.8
150	244.4	611.0
151	234.8	587.0
152	322.3	805.8
**Mean**	**242.4**	**605.9**
**SD**	**41.2**	**103.0**
**Count**	**8**	**8**

Group: 11 (0 ppm) Males		
Animal No.	g/kg body weight/ Day	mg test article/kg body weight/Day
161	205.1	0.0
162	194.5	0.0
163	265.2	0.0
164	317.4	0.0
165	220.0	0.0
166	265.6	0.0
167	275.0	0.0
168	397.1	0.0
**Mean**	**267.5**	**0.0**
**SD**	**66.4**	**0.0**
**Count**	**8**	**8**

Group: 12 (150 ppm) Males		
Animal No.	g/kg body weight/ Day	mg test article/kg body weight/Day
177	200.3	30.0
178	258.0	38.7
179	237.8	35.7
180	279.3	41.9
181	292.6	43.9
182	224.9	33.7
183	261.5	39.2
184	328.1	49.2
**Mean**	**260.3**	**39.0**
**SD**	**40.3**	**6.0**
**Count**	**8**	**8**

Group: 13 (300 ppm) Males		
Animal No.	g/kg body weight/ Day	mg test article/kg body weight/Day
193	283.5	85.1
194	289.4	86.8
195	219.7	65.9
196	216.6	65.0
197	284.4	85.3
198	248.8	74.6
199	324.0	97.2
200	351.3	105.4
**Mean**	**277.2**	**83.2**
**SD**	**47.4**	**14.2**
**Count**	**8**	**8**

Group: 14 (1250 ppm) Males		
Animal No.	g/kg body weight/ Day	mg test article/kg body weight/Day
209	248.5	310.6
210	187.6	234.5
211	245.3	306.6
212	249.9	312.4
213	227.1	283.9
214	243.7	304.6
215	291.8	364.8
216	265.4	331.8
**Mean**	**244.9**	**306.1**
**SD**	**29.9**	**37.4**
**Count**	**8**	**8**

Group: 15 (2500 ppm) Males		
Animal No.	g/kg body weight/ Day	mg test article/kg body weight/Day
225	195.8	489.5
226	274.0	685.0
227	281.1	702.8
228	225.6	564.0
229	245.3	613.3
230	313.7	784.3
231	257.3	643.3
232	274.9	687.3
**Mean**	**258.5**	**646.2**
**SD**	**36.4**	**90.9**
**Count**	**8**	**8**

Day 28

Group: 6 (0 ppm) Females		
Animal No.	g/kg body weight/ Day	mg test article/kg body weight/Day
89	258.9	0.0
90	204.6	0.0
91	304.0	0.0
92	319.8	0.0
93	144.7	0.0
94	280.9	0.0
95	496.3	0.0
96	270.9	0.0
**Mean**	**285.0**	**0.0**
**SD**	**102.3**	**0.0**
**Count**	**8**	**8**

Group: 7 (150 ppm) Females		
Animal No.	g/kg body weight/ Day	mg test article/kg body weight/Day
105	302.2	45.3
106	276.5	41.5
107	344.2	51.6
108	393.6	59.0
109	301.8	45.3
110	396.7	59.5
111	369.2	55.4
112	294.8	44.2
**Mean**	**334.9**	**50.2**
**SD**	**47.4**	**7.1**
**Count**	**8**	**8**

Group: 8 (300 ppm) Females		
Animal No.	g/kg body weight/ Day	mg test article/kg body weight/Day
121	361.5	108.5
122	296.2	88.9
123	202.3	60.7
124	444.6	133.4
125	380.4	114.1
126	259.3	77.8
127	213.9	64.2
128	205.7	61.7
**Mean**	**295.5**	**88.6**
**SD**	**91.4**	**27.4**
**Count**	**8**	**8**

Group: 9 (1250 ppm) Females		
Animal No.	g/kg body weight/ Day	mg test article/kg body weight/Day
137	258.3	322.9
138	213.4	266.8
139	179.6	224.5
140	241.8	302.3
141	306.3	382.9
142	220.5	275.6
143	190.8	238.5
144	220.2	275.3
**Mean**	**228.9**	**286.1**
**SD**	**40.2**	**50.2**
**Count**	**8**	**8**

Group: 10 (2500 ppm) Females		
Animal No.	g/kg body weight/ Day	mg test article/kg body weight/Day
153	353.3	883.3
154	304.7	761.8
155	355.3	888.3
156	293.9	734.8
157	244.4	611.1
158	265.5	663.8
159	236.8	592.0
160	412.3	1030.8
**Mean**	**308.3**	**770.7**
**SD**	**61.3**	**153.2**
**Count**	**8**	**8**

Group: 11 (0 ppm) Females		
Animal No.	g/kg body weight/ Day	mg test article/kg body weight/Day
169	412.9	0.0
170	419.4	0.0
171	307.1	0.0
172	416.9	0.0
173	378.6	0.0
174	363.2	0.0
175	284.0	0.0
176	318.7	0.0
**Mean**	**362.6**	**0.0**
**SD**	**53.6**	**0.0**
**Count**	**8**	**8**

Group: 12 (150 ppm) Females		
Animal No.	g/kg body weight/ Day	mg test article/kg body weight/Day
185	313.6	47.0
186	311.1	46.7
187	345.3	51.8
188	391.6	58.7
189	281.8	42.3
190	361.4	54.2
191	331.3	49.7
192	310.7	46.6
**Mean**	**330.9**	**49.6**
**SD**	**34.5**	**5.2**
**Count**	**8**	**8**

Group: 13 (300 ppm) Females		
Animal No.	g/kg body weight/ Day	mg test article/kg body weight/Day
201	308.4	92.5
202	354.6	106.4
203	421.5	126.5
204	448.1	134.4
205	339.1	101.7
206	342.2	102.7
207	353.2	106.0
208	374.0	112.2
**Mean**	**367.6**	**110.3**
**SD**	**45.9**	**13.8**
**Count**	**8**	**8**

Group: 14 (1250 ppm) Females		
Animal No.	g/kg body weight/ Day	mg test article/kg body weight/Day
217	336.9	421.1
218	401.6	502.0
219	306.2	382.8
220	225.8	282.3
221	349.2	436.5
222	279.6	349.5
223	292.9	366.1
224	286.3	357.9
**Mean**	**309.8**	**387.3**
**SD**	**52.8**	**66.0**
**Count**	**8**	**8**

Group: 15 (2500 ppm) Females		
Animal No.	g/kg body weight/ Day	mg test article/kg body weight/Day
233	248.5	621.3
234	322.2	805.5
235	317.2	793.0
236	295.9	739.8
237	370.8	927.0
238	312.1	780.3
239	271.7	679.3
240	259.3	648.3
**Mean**	**299.7**	**749.3**
**SD**	**39.8**	**99.4**
**Count**	**8**	**8**

Appendix IV: Necropsy Data

Pathology - Intergroup Comparison of Pathology Observations

Removal Reason: ALL

	Male				Female			
	Control	4-MEI	4-MEI	4-MEI	Control	4-MEI	4-MEI	4-MEI
Number of Animals:	8	8	8	8	8	8	8	8
Number of Completed Animals:	8	8	8	8	8	8	8	8
**BLOOD**								
Submitted	8	8	8	8	8	8	8	8
Normal	8	8	8	8	8	8	8	8
**BRAIN**								
Submitted	8	8	8	8	8	8	8	8
Normal	8	8	8	8	8	8	8	8
**KIDNEYS**								
Submitted	8	8	8	8	8	8	8	8
Normal	8	8	8	8	8	8	8	8
**LIVER**								
Submitted	8	8	8	8	8	8	8	8
Normal	8	8	8	8	8	8	8	8
Discoloration; nutmeg pattern	0	0	0	0	0	0	0	0
.... minimal	0	0	0	0	0	0	0	0
Mass	0	0	0	0	0	0	0	0
**LUNGS**								
Submitted	8	8	8	8	8	8	8	8
Normal	8	8	8	8	8	8	8	8
**OVARIES**								
Submitted	.	.	.	.	8	8	8	8
Normal	.	.	.	.	8	8	8	8

Pathology - Intergroup Comparison of Pathology Observations

Removal Reason: ALL	Male				Female			
	Control	4-MEI	4-MEI	4-MEI	Control	4-MEI	4-MEI	4-MEI
Number of Animals:	8	8	8	8	8	8	8	8
Number of Completed Animals:	8	8	8	8	8	8	8	8
**TESTES**								
Submitted	8	8	8	8	.	.	.	.
Normal	8	8	8	8	.	.	.	.

Pathology - Intergroup Comparison of Pathology Observations

Removal Reason: ALL	Male				Female			
	4-MEI	Vehicle	4-MEI	4-MEI	4-MEI	Vehicle	4-MEI	4-MEI
Number of Animals:	8	8	8	8	8	8	8	8
Number of Completed Animals:	6	8	8	8	8	8	8	8
**BLOOD**								
Submitted	6	8	8	8	8	8	8	8
Normal	6	8	8	8	8	8	8	8
**BRAIN**								
Submitted	6	8	8	8	8	8	8	8
Normal	6	8	8	8	8	8	8	8
**KIDNEYS**								
Submitted	6	8	8	8	8	8	8	8
Normal	6	8	8	8	8	8	8	8
**LIVER**								
Submitted	6	8	8	8	8	8	8	8
Normal	6	8	8	8	7	8	8	8
Discoloration; nutmeg pattern	0	0	0	0	0	0	0	0
.... minimal	0	0	0	0	0	0	0	0
Mass	0	0	0	0	1	0	0	0
**LUNGS**								
Submitted	6	8	8	8	8	8	8	8
Normal	6	8	8	8	8	8	8	8
**OVARIES**								
Submitted	.	.	.	.	8	8	8	8
Normal	.	.	.	.	8	8	8	8

Pathology - Intergroup Comparison of Pathology Observations

Removal Reason: ALL	Male				Female			
	4-MEI	Vehicle	4-MEI	4-MEI	4-MEI	Vehicle	4-MEI	4-MEI
Number of Animals:	8	8	8	8	8	8	8	8
Number of Completed Animals:	6	8	8	8	8	8	8	8
**TESTES**								
Submitted	6	8	8	8	.	.	.	.
Normal	6	8	8	8	.	.	.	.

Pathology - Intergroup Comparison of Pathology Observations

Removal Reason: ALL

	Male				Female			
	4-MEI	4-MEI	Vehicle	4-MEI	4-MEI	4-MEI	Vehicle	4-MEI
Number of Animals:	8	8	8	8	8	8	8	8
Number of Completed Animals:	8	8	8	8	8	8	8	8
**BLOOD**								
Submitted	8	8	8	8	8	8	8	8
Normal	8	8	8	8	8	8	8	8
**BRAIN**								
Submitted	8	8	8	8	8	8	8	8
Normal	8	8	8	8	8	8	8	8
**KIDNEYS**								
Submitted	8	8	8	8	8	8	8	8
Normal	8	8	8	8	8	8	8	8
**LIVER**								
Submitted	8	8	8	8	8	8	8	8
Normal	8	7	8	8	8	8	8	8
Discoloration; nutmeg pattern	0	1	0	0	0	0	0	0
.... minimal	0	1	0	0	0	0	0	0
Mass	0	0	0	0	0	0	0	0
**LUNGS**								
Submitted	8	8	8	8	8	8	8	8
Normal	8	8	8	8	8	8	8	8
**OVARIES**								
Submitted	.	.	.	.	8	8	8	8
Normal	.	.	.	.	8	8	8	8

Pathology - Intergroup Comparison of Pathology Observations

Removal Reason: ALL	Male				Female			
	4-MEI	4-MEI	Vehicle	4-MEI	4-MEI	4-MEI	Vehicle	4-MEI
Number of Animals:	8	8	8	8	8	8	8	8
Number of Completed Animals:	8	8	8	8	8	8	8	8
**TESTES**								
Submitted	8	8	8	8	.	.	.	.
Normal	8	8	8	8	.	.	.	.

Pathology - Intergroup Comparison of Pathology Observations

Removal Reason: ALL

	Male			Female		
	4-MEI	4-MEI	4-MEI	4-MEI	4-MEI	4-MEI
Number of Animals:	8	8	8	8	8	8
Number of Completed Animals:	8	8	8	8	8	8
**BLOOD**						
Submitted	8	8	8	8	8	8
Normal	8	8	8	8	8	8
**BRAIN**						
Submitted	8	8	8	8	8	8
Normal	8	8	8	8	8	8
**KIDNEYS**						
Submitted	8	8	8	8	8	8
Normal	8	8	8	8	8	8
**LIVER**						
Submitted	8	8	8	8	8	8
Normal	8	8	8	8	8	8
Discoloration; nutmeg pattern	0	0	0	0	0	0
.... minimal	0	0	0	0	0	0
Mass	0	0	0	0	0	0
**LUNGS**						
Submitted	8	8	8	8	8	8
Normal	8	8	8	8	8	8
**OVARIES**						
Submitted	.	.	.	8	8	8
Normal	.	.	.	8	8	8

Pathology - Intergroup Comparison of Pathology Observations

Removal Reason: ALL	Male			Female		
	4-MEI	4-MEI	4-MEI	4-MEI	4-MEI	4-MEI
Number of Animals:	8	8	8	8	8	8
Number of Completed Animals:	8	8	8	8	8	8
**TESTES**						
Submitted	8	8	8	.	.	.
Normal	8	8	8	.	.	.

Pathology - Intergroup Comparison of Pathology Observations

Key Page

Measurement/Statistics

Measurement	Descriptive	Comparative	Arithmetic/Adjusted	Transformation
Pathology Observation	Count Positives			

Group Information

Short Name	Long Name	Report Headings	
1	Group 1	Control	0 mg/kg
2	Group 2	4-MEI	50 mg/kg
3	Group 3	4-MEI	100 mg/kg
4	Group 4	4-MEI	200 mg/kg
5	Group 5	4-MEI	300 mg/kg
6	Group 6	Vehicle	0 ppm
7	Group 7	4-MEI	150 ppm
8	Group 8	4-MEI	300 ppm
9	Group 9	4-MEI	1250 ppm
10	Group 10	4-MEI	2500 ppm
11	Group 11	Vehicle	0 ppm
12	Group 12	4-MEI	150 ppm
13	Group 13	4-MEI	300 ppm
14	Group 14	4-MEI	1250 ppm
15	Group 15	4-MEI	2500 ppm

Pathology - Intergroup Comparison of Pathology Observations

Key Page

Removal Reason Grouping

Grouping Name	Abbreviation	Removal Reasons
Killed Terminal	Term	Killed Terminal
Killed Clinical	Clin	Killed Clinical
Killed Interim	Int	Killed Interim
Killed Moribund	Mori	Killed Moribund
Found Dead	FD	Found Dead
Mechanically Killed	Mech	Mechanically Killed
Other, See Text Comments	Oth	Other, See Text Comments

Appendix V: Certificate of Analysis and CRL Formulations Report

SIGMA-ALDRICH®

sigma-aldrich.com

3050 Spruce Street, Saint Louis, MO 63103, USA
Website: www.sigmaaldrich.com
Email USA: techserv@sial.com
Outside USA: eurtechserv@sial.com

Certificate of Analysis

Product Name:
4(5)-Methylimidazole - 98%

**Product Number:**	**199885**
**Batch Number:**	**MKBV5083V**
Brand:	ALDRICH
CAS Number:	822-36-6
MDL Number:	MFCD00005201
Formula:	C4H6N2
Formula Weight:	82.10 g/mol
Quality Release Date:	30 APR 2015

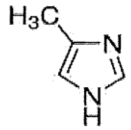

Test	Specification	Result
Appearance (Color)	White to Yellow	White
Appearance (Form)<br>Powder, Crystals, Crystalline Powder, Solid and/or Chunks	Conforms to Requirements	Solid
Infrared Spectrum	Conforms to Structure	Conforms
Titration by HCL	97.5 - 102.5 %	99.5 %
Purity (GC)	$\geq$ 97.5 %	99.9 %
Solubility (Turbidity)<br>5% in H2O	Clear	Clear
Solubility (Color)	Colorless to Yellow	Colorless

Ali Ataei, Manager
Quality Control
Milwaukee, WI US

Sigma-Aldrich warrants, that at the time of the quality release or subsequent retest date this product conformed to the information contained in this publication. The current Specification sheet may be available at Sigma-Aldrich.com. For further inquiries, please contact Technical Service. Purchaser must determine the suitability of the product for its particular use. See reverse side of invoice or packing slip for additional terms and conditions of sale.

Version Number: 1

Page 1 of 1

NOVA-TECH
PURE PERFORMANCE

CERTIFICATE OF ANALYSIS

**PRODUCT:**	Sterile Water for Injection, USP
**CAT. #:**	13255140
**LOT #:**	A1605052
**MANUFACTURE DATE:**	May 9, 2016
**EXPIRATION DATE:**	MAY 2018

TEST	SPECIFICATION	RESULT
Sterility	Sterile	Sterile
Volume	≥ 1000 mL	> 1000 mL
Appearance	Clear, colorless	Pass
Water Conductivity	≤ 5.0 µS/cm	1.6 µS/cm
Oxidizable Substances	Pass	Pass
Bacterial Endotoxin	< 0.25 EU/mL	< 0.1 EU/mL
Particulates ≥ 10 µm	≤ 25 counts/mL	Pass
Particulates ≥ 25 µm	≤ 3 counts/mL	Pass

This product meets specifications and is eligible for release.

[signature] 24MAY16

Laboratory Control
24-May-16

charles river

FINAL REPORT

Study Phase: Analytical

Test Site Phase Reference No. WIL-865004

Test Facility Study No. 50074.0001

Short Term Toxicity and Genomics Study of 4-Methylimidazole in B6C3F1 Male and Female Mice

TEST FACILITY:
Integrated Laboratory Systems, Inc.
601 Keystone Park Drive, Suite 200
Morrisville, NC 27560
United States

TEST SITE:
Charles River Laboratories Ashland, LLC
1407 George Road
Ashland, OH 44805
United States

Page 1 of 55

COMPLIANCE STATEMENT

The portions of this study conducted at Charles River, designated WIL-865004, were conducted in compliance with the Charles River SOPs and the protocol as approved by the Sponsor.

Archana Akalkotkar ________ Date: 22 Dec 2016

Archana M. Akalkotkar, PhD
Research Chemist, Analytical Chemistry
Charles River Principal Investigator

LIST OF ABBREVIATIONS

The following abbreviations may apply to this report:

4-MEI	-	4-Methylimidazole
%RE	-	percent relative error
ACN	-	acetonitrile
btm	-	bottom
CMC	-	carboxymethylcellulose
conc.	-	concentration
DI	-	deionized
DMSO	-	dimethylsulfoxide
EtOH	-	ethanol
FID	-	flame ionization detection
GC	-	gas chromatography
HCl	-	hydrochloric acid
HPLC	-	high performance liquid chromatography
hr	-	hour(s)
IS	-	internal standard
LLOQ	-	lower limit of quantitation
LM	-	laboratory method
MC	-	methylcellulose
MeOH	-	methanol
mid	-	middle
min	-	minute
MS	-	mass spectrometry
N	-	normal
ppm	-	parts per million
QC	-	quality control
r^2	-	coefficient of determination
ref	-	reference
RSD	-	relative standard deviation
S/N	-	signal-to-noise ratio
SD	-	standard deviation
sec	-	second(s)
SOP	-	standard operating procedure
theo.	-	theoretical
UHPLC	-	ultra-high performance liquid chromatography
ULOQ	-	upper limit of quantitation
UV	-	ultraviolet
v	-	volume
w	-	weight

LIST OF ABBREVIATIONS

The following abbreviations may apply to this report:

4-MEI	-	4-Methylimidazole
%RE	-	percent relative error
ACN	-	acetonitrile
btm	-	bottom
CMC	-	carboxymethylcellulose
conc.	-	concentration
DI	-	deionized
DMSO	-	dimethylsulfoxide
EtOH	-	ethanol
FID	-	flame ionization detection
GC	-	gas chromatography
HCl	-	hydrochloric acid
HPLC	-	high performance liquid chromatography
hr	-	hour(s)
IS	-	internal standard
LLOQ	-	lower limit of quantitation
LM	-	laboratory method
MC	-	methylcellulose
MeOH	-	methanol
mid	-	middle
min	-	minute
MS	-	mass spectrometry
N	-	normal
ppm	-	parts per million
QC	-	quality control
r^2	-	coefficient of determination
ref	-	reference
RSD	-	relative standard deviation
S/N	-	signal-to-noise ratio
SD	-	standard deviation
sec	-	second(s)
SOP	-	standard operating procedure
theo.	-	theoretical
UHPLC	-	ultra-high performance liquid chromatography
ULOQ	-	upper limit of quantitation
UV	-	ultraviolet
v	-	volume
w	-	weight

TABLE OF CONTENTS

COMPLIANCE STATEMENT....2
REPORT APPROVAL....3
LIST OF ABBREVIATIONS....4
LIST OF FIGURES....6
LIST OF TABLES....6
LIST OF APPENDICES....7
1. SUMMARY....8
2. INTRODUCTION....10
2.1. Study Design....10
2.2. Study Conduct and Guidelines....10
2.3. Key Study Dates....10
2.4. Sample Receipt....10
2.5. Key Study Personnel....11
2.6. Data Acquisition and Reporting....11
3. EXPERIMENTAL PROCEDURES – MATERIALS AND METHODS....12
3.1. Identification of Test Article....12
3.2. Identification of Vehicle....13
3.3. Formulation Preparation....13
4. RESULTS AND DISCUSSION....13
4.1. Specificity/Selectivity....13
4.2. Assay Qualification: Calibration Reproducibility....14
4.3. Assay Validation: Precision and Accuracy....14
4.4. Assay Acceptability....15
4.5. Test Article Stability in Calibration Standards....16
4.6. Test Article Stability in Processed Samples....16
4.7. Test Article Concentration in Formulations....16
4.8. Test Article Stability in Formulations....17
5. CONCLUSIONS....18
6. DEVIATIONS FROM THE PROTOCOL....18
7. DATA RETENTION....18

LIST OF FIGURES

Figure 1 Representative Chromatogram of a 62.5 ppm 4-MEI/mL Calibration Standard (Diet)....20

Figure 2 Representative Chromatogram of a Processed 2500 ppm 4-MEI QC Sample (Diet)....20

Figure 3 Representative Chromatogram of a Processed 2500 ppm 4-MEI Formulation Sample (Diet)....21

Figure 4 Representative Chromatogram of a Processed Control Group Formulation Sample (Diet)....21

Figure 5 Representative Chromatogram of a 10.0 µg 4-MEI/mL Calibration Standard (Gavage)....22

Figure 6 Representative Chromatogram of a Processed 30.0 mg 4-MEI/mL QC Sample (Gavage)....22

Figure 7 Representative Chromatogram of a Processed 30.0 mg 4-MEI/mL Formulation Sample (Gavage)....23

Figure 8 Representative Chromatogram of a Processed Vehicle Blank Sample (Gavage)....23

LIST OF TABLES

Table 1 Back-Calculated Concentrations of the Qualification Calibration Standards (Diet)....25

Table 2 Back-Calculated Concentrations of the Qualification Calibration Standards (Gavage)....26

Table 3 Back-Calculated Concentrations of the Qualification Quality Control Samples (Diet)....27

Table 4 Back-Calculated Concentrations of the Qualification Quality Control Samples (Gavage)....28

Table 5 Minimum 15-Hour Room Temperature Stability Assessment of Calibration Standard and Processed Quality Control Samples....29

Table 6 Concentration/Stability Assessment of the 26 Sep 2016 Diet Formulations....30

Table 7 Concentration Assessment of the 10 Oct 2016 Diet Formulations....31

Table 8 Concentration Assessment of the 27 Sep 2016 Gavage Formulations....32

Table 9 At Least 10-Day Room Temperature Stability Assessment of the 26 Sep 2016 Diet Formulations 33

Table 10 16-Day (7-Day Refrigerated and 9-Day Room Temperature) Stability Assessment of 26 Sep 2016 Diet Formulations 34

Table 11 Concentration Assessment of the 06 Sep 2016 Gavage Formulations 35

Table 12 At Least 3-Day Refrigerated Storage Stability Assessment of the 06 Sep 2016 Gavage Formulations 36

LIST OF APPENDICES

Appendix 1 Test Material Information 37

Appendix 2 Laboratory Methods 39

1. SUMMARY

An HPLC method using UV absorbance detection at a wavelength of 220 nm for the determination of 4-MEI (4-Methylimidazole) concentration in NTP 2000 feed formulations and test article ranging in concentration from 125 to 2500 ppm was qualified in this study. Also in this study, test article stability was assessed in diet formulations prepared at target concentrations of 150, 300, 1250, and 2500 ppm stored at room temperature for at least 1 or 7 days or stored refrigerated for at least 7 days followed by room temperature storage for at least 9 days (16 days). In this study, an HPLC method using UV absorbance detection at a wavelength of 220 nm for the determination of 4-MEI concentration in gavage formulations containing sterile water for injection and test article ranging in concentration from 5.00 to 30.0 mg 4-MEI/mL was also qualified. Test article stability was also assessed in gavage formulations at target test article concentration of 5, 10, 20, and 30 mg 4-MEI/mL following 1 or 3 days of refrigerated storage. Finally, test article concentration was assessed in gavage formulations prepared at target concentrations of 5, 10, 20, and 30 mg 4-MEI/mL and in diet formulations prepared at target 4-MEI concentration of 150, 300, 1250, and 2500 ppm. No test article was detected in the analyzed vehicle or diet administered to the control group.

The 4-MEI assay procedure was qualified in this study with single qualification session in diet formulations and subsequently cross-qualified with a single qualification session in gavage formulations. Quantitation was performed using calibration standards ranging from 62.5 and 2500 ppm for the diet formulations and 10.0 and 100 µg/mL for the gavage formulations. The intra-session variability (RSD) and %RE of the mean back-calculated standard concentrations of the calibration standards prepared for the qualification and cross-qualification, respectively, are summarized in Text Table 1 and Text Table 2.

Text Table 1
Back-Calculated Concentrations of the Qualification Calibration Standards – Diet Formulations

Qualification	RSD Range of Values (%)	%RE Range of Values (%)
Single Session	1.6 to 8.7	-1.0 to 0.66

Text Table 2
Back-Calculated Concentrations of the Qualification Calibration Standards – Gavage Formulations

Qualification	RSD Range of Values (%)	%RE Range of Values (%)
Single Session	0.28 to 1.9	-1.8 to 3.0

The results met the SOP-specified acceptance criteria for calibration standards, i.e., RSD ≤ 10% and %RE within ± 10% (except at the lowest calibration level where RSD ≤ 10% and %RE within ± 15% were acceptable).

Assay precision and accuracy were verified by the analysis of QC samples. The intra-session variability (precision) and %RE (accuracy) of the mean calculated QC concentrations of the

2. INTRODUCTION

Due to the acquisition of WIL Research by Charles River, the name of the WIL Research facility in Ashland, OH has been changed to Charles River Laboratories Ashland, LLC, 1407 George Road, Ashland, OH 44805, USA. Study documents may contain both names and both names are considered equivalent and may be used as the name as WIL Research transitions to Charles River.

2.1. Study Design

An HPLC method using UV absorbance detection at a wavelength of 220 nm for the determination of 4-MEI concentration in NTP 2000 feed formulations and test article ranging in concentration from 125 to 2500 ppm was qualified in this study. Also in this study, test article stability was assessed in diet formulations prepared at target concentrations of 150, 300, 1250, and 2500 ppm stored at room temperature for at least 1 or 7 days or stored refrigerated for at least 7 days followed by room temperature storage for at least 9 days (16 days) was assessed. In this study, an HPLC method using UV absorbance detection at a wavelength of 220 nm for the determination of 4-MEI concentration in gavage formulations containing sterile water for injection and test article ranging in concentration from 5.00 to 30.0 mg 4-MEI/mL was also qualified. Finally, test article concentration was assessed in gavage formulations prepared at target concentrations of 5, 10, 20, and 30 mg 4-MEI/mL and in diet formulations prepared at target 4-MEI concentration of 150, 300, 1250, and 2500 ppm.

2.2. Study Conduct and Guidelines

The portions of this study conducted at Charles River were conducted according to the Charles River SOPs and the study protocol as approved by the Sponsor.

2.3. Key Study Dates

**Date(s)**	**Event(s)**
07 Sep 2016	First date of analysis
12 Oct 2016	Last date of analysis

2.4. Sample Receipt

Samples were collected at Integrated Laboratory Systems, LLC (Morrisville, NC) on 06 Sep 2016 and 27 Sep 2016 and shipped to Charles River on ice packs on 06 Sep 2016 and 27 Sep 2016, respectively. Samples were received the following day in good condition and were processed upon receipt.

2. INTRODUCTION

Due to the acquisition of WIL Research by Charles River, the name of the WIL Research facility in Ashland, OH has been changed to Charles River Laboratories Ashland, LLC, 1407 George Road, Ashland, OH 44805, USA. Study documents may contain both names and both names are considered equivalent and may be used as the name as WIL Research transitions to Charles River.

2.1. Study Design

An HPLC method using UV absorbance detection at a wavelength of 220 nm for the determination of 4-MEI concentration in NTP 2000 feed formulations and test article ranging in concentration from 125 to 2500 ppm was qualified in this study. Also in this study, test article stability was assessed in diet formulations prepared at target concentrations of 150, 300, 1250, and 2500 ppm stored at room temperature for at least 1 or 7 days or stored refrigerated for at least 7 days followed by room temperature storage for at least 9 days (16 days) was assessed. In this study, an HPLC method using UV absorbance detection at a wavelength of 220 nm for the determination of 4-MEI concentration in gavage formulations containing sterile water for injection and test article ranging in concentration from 5.00 to 30.0 mg 4-MEI/mL was also qualified. Finally, test article concentration was assessed in gavage formulations prepared at target concentrations of 5, 10, 20, and 30 mg 4-MEI/mL and in diet formulations prepared at target 4-MEI concentration of 150, 300, 1250, and 2500 ppm.

2.2. Study Conduct and Guidelines

The portions of this study conducted at Charles River were conducted according to the Charles River SOPs and the study protocol as approved by the Sponsor.

2.3. Key Study Dates

Date(s)	Event(s)
07 Sep 2016	First date of analysis
12 Oct 2016	Last date of analysis

2.4. Sample Receipt

Samples were collected at Integrated Laboratory Systems, LLC (Morrisville, NC) on 06 Sep 2016 and 27 Sep 2016 and shipped to Charles River on ice packs on 06 Sep 2016 and 27 Sep 2016, respectively. Samples were received the following day in good condition and were processed upon receipt.

2.5. Key Study Personnel

Principal Investigator:

Archana M. Akalkotkar, PhD	Research Chemist, Analytical Chemistry

Study Personnel:

Tara E. Sansom, BS	Chemist II, Analytical Chemistry
Sara M. Stanish, BS	Chemist III, Analytical Chemistry
Christopher S. Farabaugh, PhD	Director, Analytical Services and Site Director, Skokie
Heather L. Johnson, BS, RQAP-GLP	Senior Manager, Regulatory Compliance
Jennifer L. Starkey, AS, LAT	Manager and Archivist, Archives
Andrew M. Vick, PhD	Corporate Vice President, Safety Assessment Ohio
Sarah E. Brockway, BS	Data Coordinator, Reporting & Technical Support Services
Jennifer R. Hicks, AS	Publishing Specialist, Reporting & Technical Support Services
Robert A. Wally, BS	Operations Manager, Reporting & Technical Support Services

Report Reviewed by:

Kallol Biswas, PhD	Director, Analytical Chemistry and Formulations
Gregory A. Hawks, AS	Group Supervisor, Reporting & Technical Support Services

2.6. Data Acquisition and Reporting

The major computer systems used on this study include, but are not limited to, the following systems. All computerized systems used for data collection during the conduct of this study have been validated (with the exception of Microsoft® Office® and GraphPad Prism® 2008); when a particular system has not satisfied all requirements, appropriate administration and procedural controls were implemented to assure the quality and integrity of the data.

Text Table 5
Critical Computerized Systems

Program/System	Description
Adobe® Acrobat®	Used in conjunction with Microsoft® Office® and the publishing software to generate study reports.
Archive Management System (AMS)	In-house developed application for storage, maintenance, and retrieval of information for archived materials (e.g., lab books, study data, wet tissues, slides, etc.).
Dionex Chromeleon® software, ver. 6.8	Used for chromatographic data acquisition and quantitation.
InSight® Publisher	Electronic publishing system (output is Adobe® Acrobat®, PDF).
Master Schedule	Maintains the master schedule for the company.
Metasys DDC Electronic Environmental Control System	In-house developed system used to record and report refrigerator/freezer conditions.
Microsoft® Office® 2007 or higher	Used in conjunction with the publishing software to generate study reports. Used in conjunction with data acquisition software for statistical calculations.
Provantis Dispense™	Comprehensive system (Instem LSS Limited) to manage test materials, including receipt, formulation instructions, and accountability.

3. EXPERIMENTAL PROCEDURES – MATERIALS AND METHODS

The materials and methods used during the course of these analyses are documented in LM nos. 865.RDT.01 and 865.WFI1.01 (presented in Appendix 2).

3.1. Identification of Test Article

Identification: 4(5)-Methylimidazole

- Batch (Lot) No.: MKBV5083V
- Log No.: ARS-011541A and 160324
- Receipt Date: 14 Sep 2016
- Expiration Date: 27 Jul 2017
- Physical Description: Pale, yellow crystal
- Storage Conditions: Kept at room temperature, protected from light
- Supplier: Integrated Laboratories System, LLC

Documentation regarding the purity and stability of the test article is on file with the Sponsor and Charles River. A Certificate of Analysis for the test article was provided by the Sponsor and is presented in Appendix 1.

3.2. Identification of Vehicle

The vehicle used in preparation of the test article formulations was sterile water for injection and NTP-2000 diet, prepared using:

- Sterile water for injection (lot no.WFI177, exp. date: 31 Oct 2016)
- Sterile water for injection (lot no.WFI188, exp. date: 01 Sep 2017)
- Rodent NTP-2000 Assay (lot no.07-01-16, exp. date: 01 Jan 2017)

3.3. Formulation Preparation

Formulations were prepared at the test article concentrations indicated in Text Table 6.

Text Table 6
Test Article Formulations - Diet

Group	Test Article	Concentration (mg/mL)
6/11	4-MEI	0
7/12	4-MEI	150
8/13	4-MEI	300
9/14	4-MEI	1250
10/15	4-MEI	2500

The required amount of test article was ground and added to acetone in a glass container. The mixture was stirred and added to a portion of feed. The remaining amount of feed was added to achieve the final weight. The formulation was pre-mixed for 10 minutes followed by mixing in V-blender for 15 minutes. The first and last 5 minutes in the V-blender was mixed with intensifier bar.

4. RESULTS AND DISCUSSION

Under the described chromatographic conditions, the retention time of the test article was approximately 4.0 minutes in diet formulations and approximately 4.6 minutes in gavage samples. Figure 1 through Figure 4 are typical chromatograms of a calibration standard, a processed QC sample, a processed formulation sample, and a processed control group formulation sample, respectively, for diet formulations and Figure 5 through Figure 8 are typical chromatograms of a calibration standard, a processed QC sample, a processed formulation sample, and a processed vehicle blank sample, respectively, for gavage formulations. The total analysis time required for each run was 10.0 minutes.

4.1. Specificity/Selectivity

As shown in Figure 4 and Figure 8 (and in contrast to the chromatograms shown in Figure 1, Figure 2, Figure 3, and Figure 5, Figure 6, and Figure 7), assay specificity/selectivity was confirmed when HPLC/UV analysis of processed vehicle samples revealed no significant peaks

(with S/N >10) at or near the retention time for the test article (approximately 4.0 minutes for diet samples and approximately 4.6 minutes for gavage samples).

4.2. Assay Qualification: Calibration Reproducibility

During the single qualification session and the subsequent single cross-qualification session, triplicate calibration standards at 5 concentrations were prepared and analyzed as described in Appendix 2 (Laboratory Methods). Single injections were made of each calibration standard. The resulting 4-MEI peak area versus theoretical 4-MEI concentration data were fit to the linear function using least-squares regression analysis. The results of the regression analyses were used to back-calculate the corresponding concentrations from the peak area data. As per SOP, the reproducibility of the calibration curve data was considered valid when the mean back-calculated concentrations at each calibration level were within ± 10% of the theoretical values (%RE within ± 10%), except at the lowest calibration level where %RE within ± 15% was acceptable.

The back-calculated concentrations and the associated intra-session statistics for the 4-MEI assay qualification and cross-qualification calibration standards are summarized in Table 1 and Table 2, respectively, with the inter- or intra-session variability (RSD) of the back-calculated concentrations and the %RE of the inter- or intra-session mean concentrations summarized in Text Table 7 and Text Table 8.

Text Table 7
Calibration Reproducibility – Diet Formulations

Qualification	RSD Range of Values (%)	%RE Range of Values (%)
Single session	1.6 to 8.7	-1.0 to 0.66

Text Table 8
Calibration Reproducibility – Gavage Formulations

Qualification	RSD Range of Values (%)	%RE Range of Values (%)
Single session	0.28 to 1.9	-1.8 to 3.0

Based on the stated criteria, the reproducibility of the 4-MEI calibration data was acceptable.

4.3. Assay Validation: Precision and Accuracy

During the single method qualification session and the subsequent single cross-qualification session, triplicate QC samples at 3 concentrations for gavage formulations and at 4 concentrations for diet formulations were prepared and analyzed as described in Appendix 2 (Laboratory Methods). Single injections were made of each processed QC sample. The results of the regression analyses were used to calculate the corresponding concentrations from the QC peak area data. The variability (RSD) of the calculated QC concentration data was used as a measure of assay precision, and the difference between theoretical and the calculated mean QC concentrations (%RE) was used as a measure of assay accuracy. According to SOP, the

precision of the method was considered acceptable when the inter-session RSD of the calculated concentrations at each QC level was ≤ 10% for gavage samples or ≤ 15% for diet samples, and the accuracy of the method was considered acceptable when the intra-session calculated mean concentration at each QC level had a %RE value within ± 10% for gavage samples or ± 15% for diet samples.

The calculated concentrations and the associated intra-session statistics for the 4-MEI assay qualification and cross-qualification QC samples are summarized in Table 3 and Table 4, respectively, with the intra-session variability (RSD) of the calculated concentrations of each QC sample (precision) and the %RE values (accuracy) of the inter- or intra-session mean concentrations of the QC samples summarized in Text Table 9 and Text Table 10.

Text Table 9
Precision and Accuracy- Diet Formulations

Qualification	Concentration Range (ppm)	RSD Range of Values (%)	%RE Range of Values (%)
Single Session	125 to 2500	0.60 to 1.0	-9.3 to -4.5

Text Table 10
Precision and Accuracy- Diet Formulations

Qualification	Concentration Range (mg/mL)	RSD Range of Values (%)	%RE Range of Values (%)
Single Session	5.00 to 30.0	0.16 to 3.0	-5.4 to 1.0

Based on the previously stated criteria, the precision and accuracy of the 4-MEI assay was acceptable

4.4. Assay Acceptability

In addition to the experimental samples, each analytical session consisted of (but was not limited to) calibration standards at 5 concentrations and triplicate QC samples prepared at each of 3 concentrations for gavage samples and 4 concentrations for diet samples. In this study, the formulations were prepared at target concentrations of 0, 150, 300, 1250, and 2500 ppm 4-MEI, and the QC samples were prepared at nominal concentrations of 125, 625, 1250, and 2500 ppm 4-MEI for diet samples. For the gavage samples, the formulations were prepared at target concentrations of 0, 5, 10, 20, and 30 mg 4-MEI/mL, and the QC samples were prepared at nominal concentrations of 5.00, 25.0, and 30.0 mg 4-MEI/mL. According to SOP, for an analytical session to be considered valid, at least two-thirds of the calculated QC concentrations with at least 1 sample at each concentration had to be 90% to 110% for gavage samples or 85% to 115% for diet samples of the nominal QC concentration. All reported results were from analytical sessions that met the acceptance criteria.

4.5. Test Article Stability in Calibration Standards

Calibration standards prepared at 62.5 and 2500 ppm 4-MEI and analyzed were stored at room temperature for 15 hours before re-analysis to assess test article stability. The mean post-storage concentrations ranged from 101% to 152% of the pre-storage values (Table 5), which met the SOP-specified requirement for stability (i.e., the mean post-storage concentration was not < 90% of the pre-storage value). The broadening of the peak at the lower concentrations resulted in an increase in the peak area and eventually the concentration.

4.6. Test Article Stability in Processed Samples

Quality control samples prepared at nominal test article concentrations of 125 and 2500 ppm 4-MEI were processed and analyzed. The processed samples were stored at room temperature for 15 hours before re-analysis to assess test article stability. The mean post-storage concentrations were 104% and 110% of the pre-storage values (Table 5), which met the previously stated SOP-specified requirement for stability.

4.7. Test Article Concentration in Formulations

Formulations used for dose administration were analyzed to assess test article concentration acceptability. The results of the concentration acceptability assessments are presented in Table 6, Table 7, and Table 8, with the mean concentration and percent of target values summarized in Text Table 11 and Text Table 12.

Text Table 11
Test Article Concentration in Formulations-Diet Formulations

Date of Preparation	Mean Concentration, ppm (% of Target)				
	**Group 6/11 0 ppm**	**Group 7/12 150 ppm**	**Group 8/13 300 ppm**	**Group 9/14 1250 ppm**	**Group 10/15 2500 ppm**
26 Sep 2016	ND (NA)	122.6 (81.7)	232 (77.4)	998 (79.9)	1981 (79.2)
10 Oct 2016	ND (NA)	150 (100)	264 (88.0)	1028 (82.3)	2122 (84.9)

ND = Not detectable; NA = Not applicable

Text Table 12
Test Article Concentration in Formulations-Gavage Formulations

Date of Preparation	Mean Concentration, mg/mL (% of Target)				
	**Group 1 (0 mg/mL)**	**Group 2 (5 mg/mL)**	**Group 3 (10 mg/mL)**	**Group 4 (20 mg/mL)**	**Group 5 (30 mg/mL)**
27 Sep 2016	ND (NA)	5.12 (102)	10.1 (101)	20.7 (103)	30.9 (103)

ND = Not detectable; NA = Not applicable

The analyzed aqueous formulations used for dose administration met the protocol-specified requirement for concentration acceptability for solution formulations, i.e., the analyzed

concentration was 85% to 115% of the target concentration. No test article was detected in the analyzed vehicle administered to the control group (Group 1). Also, the analyzed diet formulations used for dose administration did not meet the protocol-specified requirement for concentration acceptability for diet formulations, i.e., the analyzed concentration was 85% to 115% of the target concentration, with the exception of Group 12 and Group 13 prepared on 10 Oct 2016. No test article was detected in the analyzed vehicle administered to the control group (Group 6/11). It is believed that there was an extraction issue of the test article from the dietary formulations. Hence bath sonication as well as probe sonication was performed on dietary samples to maximize test article extraction.

4.8. Test Article Stability in Formulations

Formulations prepared at target 4-MEI concentrations of 150, 300, 1250, and 2500 ppm for diet samples and at 5, 10, 20, and 30 mg/mL for gavage samples. Portions of the formulations were stored at room temperature or refrigerated, before being analyzed to assess test article stability. The stability results are presented in Table 9 through Table 12, with the mean concentration and percent of time-zero values summarized in Text Table 13 and Text Table 14.

Text Table 13
Test Article Stability in Diet Formulations

Storage Condition	Storage Duration	Mean Concentration, ppm (% of Target)			
		**Group 7/12 150 ppm**	**Group 8/13 300 ppm**	**Group 9/14 1250 ppm**	**Group 10/15 2500 ppm**
Room Temperature	1 Day	122.6 (81.7)	232 (77.4)	998 (79.9)	1981 (79.2)
	11 Days	147 (98.1)	247 (82.5)	889 (71.1)	1702 (68.1)
Refrigerated/Room Temperature	7-Day Refrigerated + 9-Day Room Temperature	144 (95.9)	269 (89.6)	1007 (80.6)	1977 (79.1)

Text Table 14
Test Article Stability in Gavage Formulations

Storage Condition	Storage Duration	Mean Concentration, mg/mL (% of Target)			
		**Group 2 (5 mg/mL)**	**Group 3 (10 mg/mL)**	**Group 4 (20 mg/mL)**	**Group 5 (30 mg/mL)**
Refrigerated	1 Day	5.14 (103)	9.80 (98.0)	19.2 (95.8)	29.3 (97.6)
	3 Days	5.33 (107)	10.5 (105)	21.5 (107)	32.2 (107)

The post-storage test article concentrations ranged from 68.1% to 98.1% of the pre-storage values for the diet samples, which did not meet the previously stated protocol-specified acceptance criteria for stability. Also, the post-storage test article concentrations for gavage samples ranged from 95.8% to 107% of the pre-storage values, which met the previously stated protocol-specified acceptance criteria for stability. The results were accepted by the Study Director. The reasons for out-of-specification results for the post-storage dietary samples are

similar to as explained in Section 4.7. (Test Article Concentration in Formulations) due to insufficient extraction of test article from the diet.

5. CONCLUSIONS

An HPLC method using UV absorbance detection at a wavelength of 220 nm for the determination of 4-MEI concentration in NTP 2000 feed formulations and test article ranging in concentration from 125 to 2500 ppm was qualified in this study. Method specificity/selectivity, calibration reproducibility, precision and accuracy were assessed and qualified, satisfying SOP- and/or protocol-specified criteria. Also, in this study, an HPLC method using UV absorbance detection at a wavelength of 220 nm for the determination of 4-MEI concentration in gavage formulations containing sterile water for injection and test article ranging in concentration from 5.00 to 30.0 mg 4-MEI/mL was also qualified. Method specificity/selectivity, calibration reproducibility, precision and accuracy were assessed and qualified, satisfying SOP and/or protocol-specified criteria. Test article stability was assessed in diet formulations prepared at target concentrations of 150, 300, 1250, and 2500 ppm stored at room temperature for at least 1 or 7 days or stored refrigerated for at least 7 days followed by room temperature storage for at least 9 days (16 days) which did not meet the previously stated protocol-specified acceptance criteria for stability. Test article stability was also assessed in gavage formulations at target test article concentration of 5, 10, 20, and 30 mg 4-MEI/mL following 1 or 3 days of refrigerated storage which met the protocol-specified acceptance criteria for stability. Finally, test article concentration was assessed in gavage formulations prepared at target concentrations of 5, 10, 20, and 30 mg 4-MEI/mL and in diet formulations prepared at target 4-MEI concentration of 150, 300, 1250, and 2500 ppm that met the protocol-specified acceptance criteria for concentration with the following exception. The diet samples prepared for all groups on 26 Sep 2016 and the diet samples prepared for Group 14 and Group 15 prepared on 10 Oct 2016 failed to meet the protocol-specified acceptance criteria for concentration. No test article was detected in the analyzed vehicle or diet administered to the control group.

6. DEVIATIONS FROM THE PROTOCOL

There were no deviations from the study protocol.

7. DATA RETENTION

The Sponsor has title to all documentation records, raw data, specimens, or other work product generated during the performance of the study. Work product generated by Charles River, including raw data (as well as pertinent electronic storage media) and specimens, are retained in the Charles River Archives as specified in the study protocol. Reserve samples of the test and control articles (if appropriate) and the original final report are retained in the Charles River Archives in compliance with regulatory requirements.

FIGURES

Figure 1
Representative Chromatogram of a 62.5 ppm 4-MEI/mL Calibration Standard (Diet)

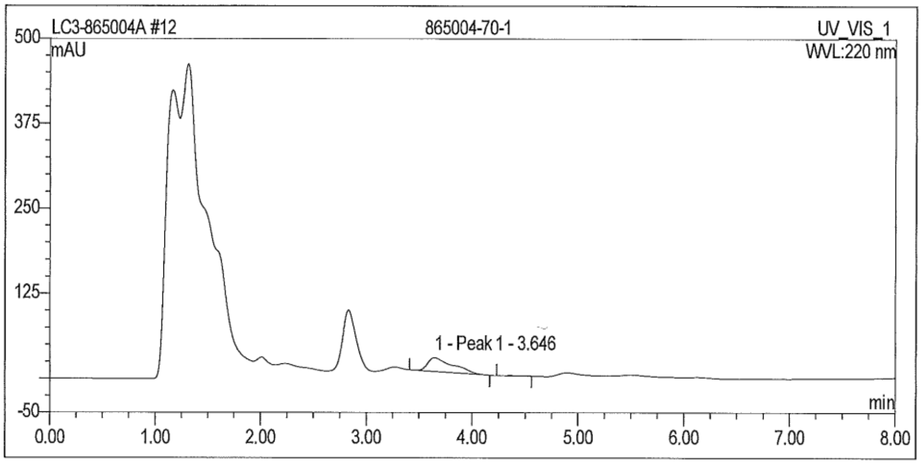

Figure 2
Representative Chromatogram of a Processed 2500 ppm 4-MEI QC Sample (Diet)

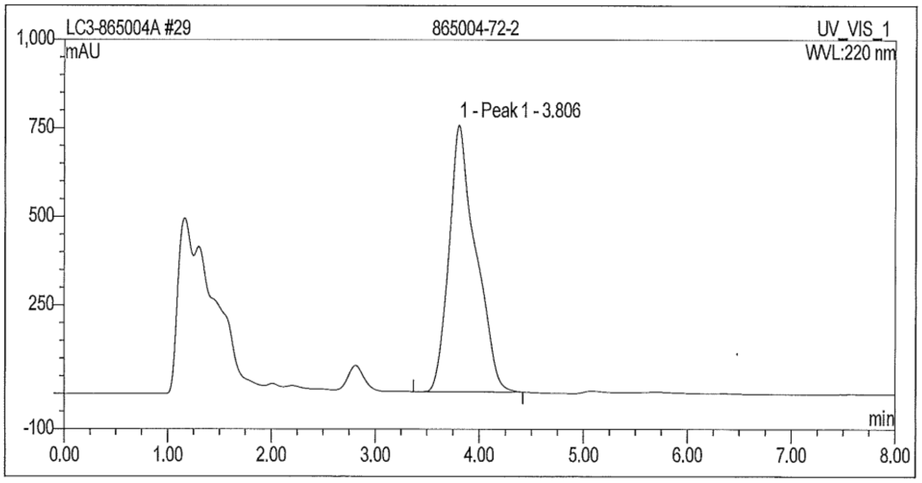

Test Facility Study No. 50074.0001

Test Site Reference No. WIL-865004
Page 20

Figure 3
Representative Chromatogram of a Processed 2500 ppm 4-MEI Formulation Sample (Diet)

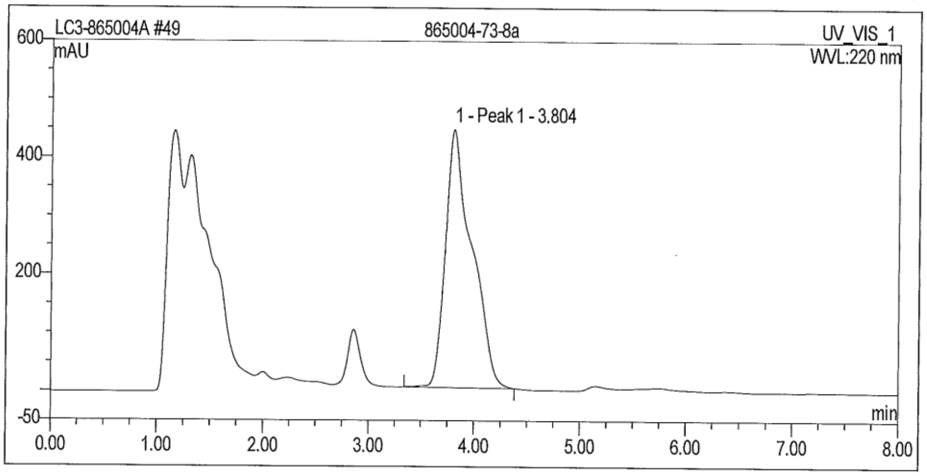

Figure 4
Representative Chromatogram of a Processed Control Group Formulation Sample (Diet)

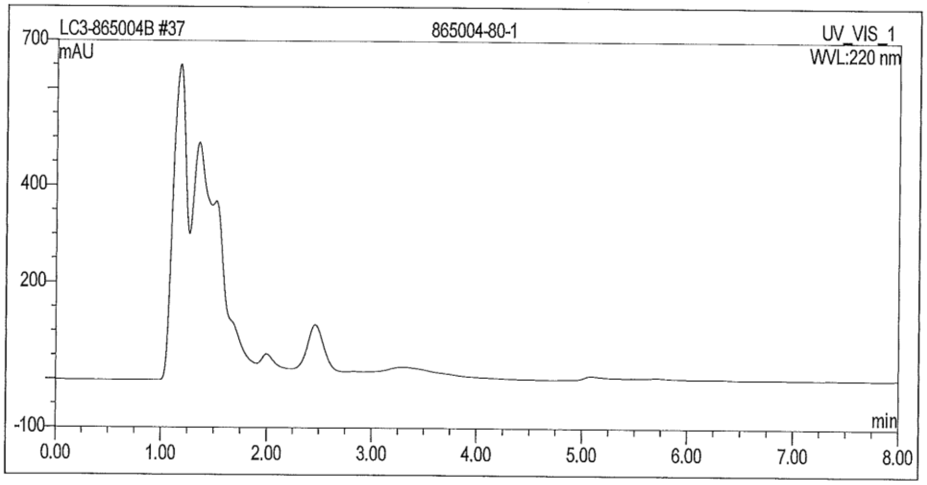

Test Facility Study No. 50074.0001

Test Site Reference No. WIL-865004
Page 21

Figure 5
Representative Chromatogram of a 10.0 µg 4-MEI/mL Calibration Standard (Gavage)

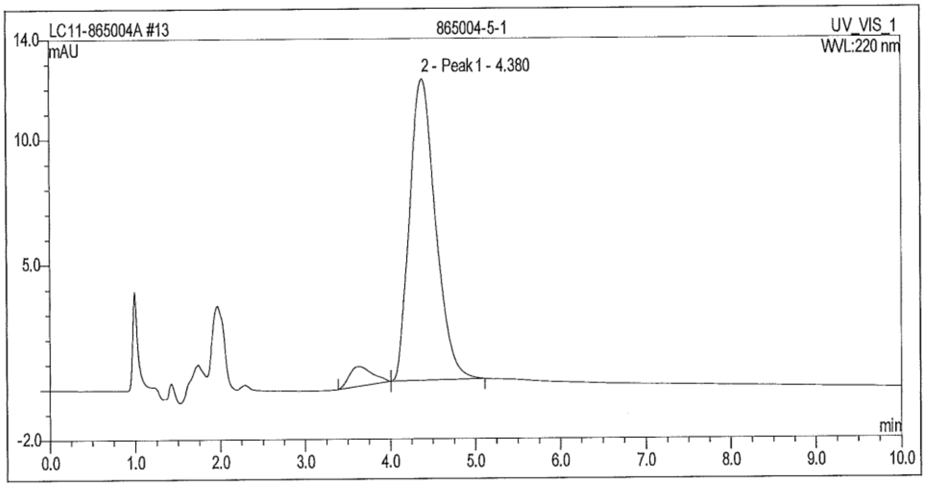

Figure 6
Representative Chromatogram of a Processed 30.0 mg 4-MEI/mL QC Sample (Gavage)

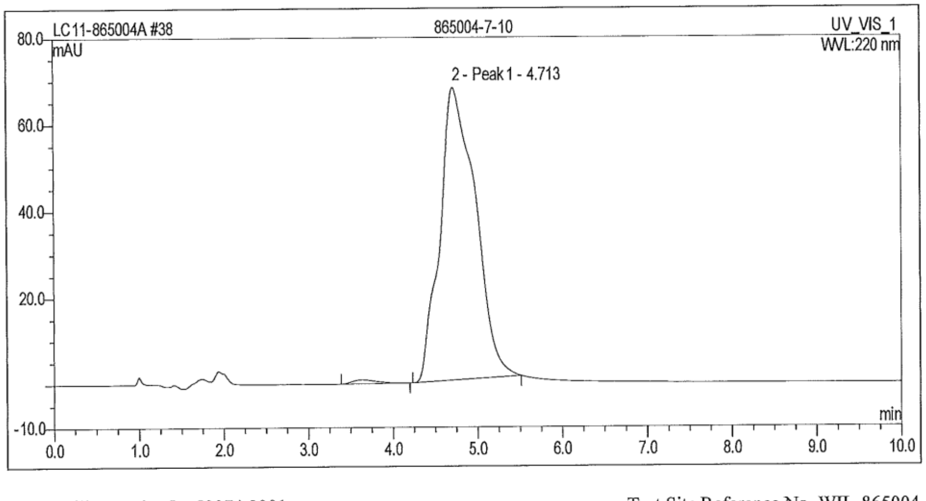

Test Facility Study No. 50074.0001

Test Site Reference No. WIL-865004
Page 22

Figure 7
Representative Chromatogram of a Processed 30.0 mg 4-MEI/mL Formulation Sample (Gavage)

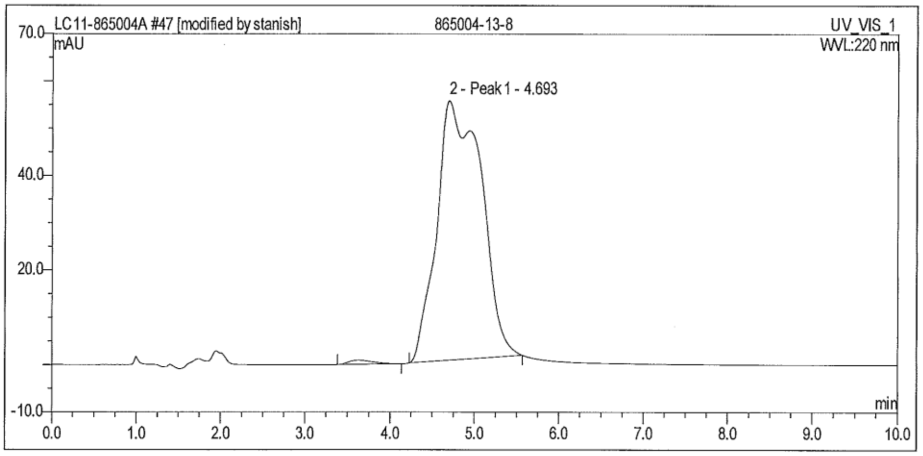

Figure 8
Representative Chromatogram of a Processed Vehicle Blank Sample (Gavage)

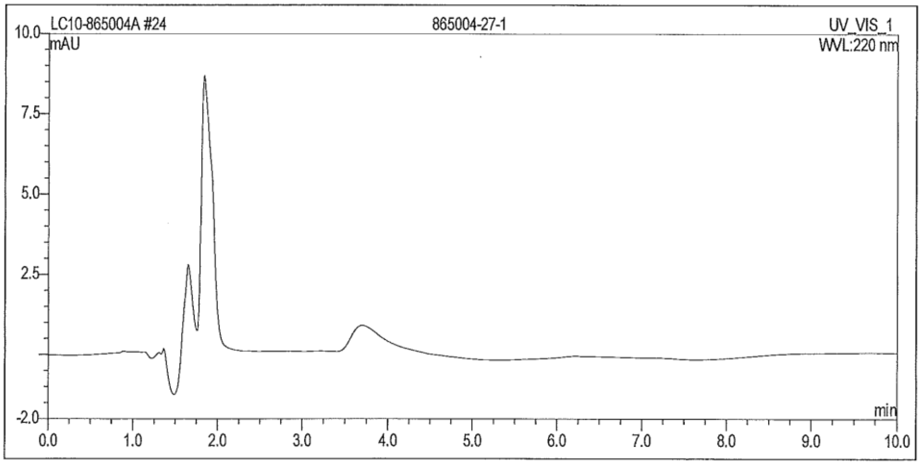

TABLES

Table 1
Back-Calculated Concentrations of the Qualification Calibration Standards
(Diet)

**Concentration (ppm)**	**62.5**	**125**	**625**	**1250**	**2500**
(28 Sep 2016)	67.7	129	640	1280	2576
	57.1	120	627	1244	2477
	60.8	123	620	1218	2448
Mean	61.9	124	629	1247	2500
SD	5.4	4.4	10	31	67
%RSD	8.7	3.6	1.6	2.5	2.7
%RE	-1.0	-0.91	0.66	-0.23	0.019

865004 results.xls I-Diet (2)
Printed: 06Nov2016 2:12 PM

Table 2
Back-Calculated Concentrations of the Qualification Calibration Standards
(Gavage)

Concentration (µg/mL)	10.0	25.0	50.0	75.0	100
(07 Sep 2016)	10.1	25.0	49.0	75.8	100
	10.5	24.7	49.3	76.8	99.0
	10.2	24.7	49.1	76.2	99.1
Mean	10.3	24.8	49.1	76.2	99.5
SD	0.20	0.16	0.14	0.51	0.82
%RSD	1.9	0.63	0.28	0.67	0.83
%RE	3.0	-0.76	-1.8	1.7	-0.47

865004 results.xls I -Gavage
Printed: 06Nov2016 2:12 PM

Table 3
Back-Calculated Concentrations of the Qualification Quality Control Samples
(Diet)

**Concentration (ppm)**	**125**	**625**	**1250**	**2500**
(07 Sep 2016)	112	595	1178	2371
	115	593	1163	2411
	113	588	1176	2381
Mean	113	592	1172	2388
SD	1.1	3.5	8	21
%RSD	1.0	0.60	0.68	0.87
%RE	-9.3	-5.3	-6.2	-4.5

865004 results.xls II-Diet (2)
Printed: 06Nov2016 2:12 PM

Table 4
Back-Calculated Concentrations of the Qualification Quality Control Samples
(Gavage)

Concentration (mg/mL)	5.00	25.0	30.0
(07 Sep 2016)	4.99	23.7	29.1
	5.22	23.1	29.2
	4.94	24.2	29.1
Mean	5.05	23.7	29.1
SD	0.15	0.51	0.045
%RSD	3.0	2.1	0.16
%RE	1.0	-5.4	-2.9

865004 results.xls II-Gavage
Printed: 06Nov2016 2:12 PM

Table 5
Minimum 15-Hour Room Temperature Stability Assessment of Calibration Standard and Processed Quality Control Samples

Times Analyzed (t= hours)	Theo. Conc (µg/mL)	Ref # (865004 -)	Run #	Back Calculated Conc. (µg/mL)	Percent of Time Zero (%)	Overall Percent of Time Zero (%)
Calibration Standards						
t= 0	62.5	77 - 2	75	68.5	N/A	152
t= 15		77 - 2	129	106	154	
t= 0	62.5	77 - 3	76	69.2	N/A	
t= 15		77 - 3	130	103	149	
t= 0	2500	77 - 14	87	2512	N/A	101
t= 15		77 - 14	124	2533	101	
t= 0	2500	77 - 15	88	2489	N/A	
t= 15		77 - 15	125	2515	101	

Times Analyzed	Theo. Conc (mg/mL)	Ref # (865004 -)	Run #	Calculated Conc. (mg/mL)	Percent of Time Zero (%)	Overall Percent of Time Zero (%)
QC Samples						
t= 0	125	79 - 5	94	138	N/A	110
t= 15		79 - 5	133	140	101	
t= 0	125	79 - 6	95	139	N/A	
t= 15		79 - 6	134	165	119	
t= 0	2500	79 - 2	91	2392	N/A	104
t= 15		79 - 2	135	2472	103	
t= 0	2500	79 - 3	92	2412	N/A	
t= 15		79 - 3	136	2537	105	

865004 results.xls pss
Printed: 11/06/16 2:30 PM

Table 6

Concentration/Stability Assessment of the 26 Sep 2016 Diet Formulations

(Analyzed 28 Sep 2016)

Dose Conc (ppm)	Ref # (865004 -)	Run #	Analyzed Conc (ppm)	Percent of Target (%)	Mean Conc (ppm)	SD	RSD (%)	Mean Conc % of Target (%)
0	44A - 9	304			---Not Detected---			
	44A - 10	305						
150	44A - 1	296	126	84.1	122.6	5.1	4.2	81.7
	44A - 2	297	119	79.3				
300	44A - 3	298	228	76.2	232	5.1	2.2	77.4
	44A - 4	299	236	78.6				
1250	44A - 5	300	997	79.8	998	1.2	0.12	79.9
	44A - 6	301	999	79.9				
2500	44A - 7	302	1956	78.3	1981	35	1.8	79.2
	44A - 8	303	2006	80.2				

865004 results.xls 2C-Diet
Printed: 06Nov2016 2:30 PM

Table 7
Concentration Assessment of the 10 Oct 2016 Diet Formulations
(Analyzed 11 Oct 2016)

Dose Conc (ppm)	Ref # (865004 -)	Run #	Analyzed Conc (ppm)	Percent of Target (%)	Mean Conc (ppm)	SD	RSD (%)	Mean Conc % of Target (%)
0	80 - 1	104			---Not Detected---			
	80 - 2	105						
150	80 - 3	106	149	99.6	150	1.2	0.8	100
	80 - 4	107	151	101				
300	80 - 5	108	263	87.7	264	1.2	0.5	88.0
	80 - 6	109	265	88.3				
1250	80 - 7	110	1011	80.9	1028	25	2.4	82.3
	80 - 8	111	1046	83.7				
2500	80 - 9	112	2098	83.9	2122	34	1.6	84.9
	80 - 10	113	2146	85.8				

865004 results.xls 3C-Diet
Printed: 06Nov2016 2:30 PM

Table 8
Concentration Assessment of the 27 Sep 2016 Gavage Formulations
(Analyzed 28 Sep 2016)

Dose Conc (mg/mL)	Ref # (865004 -)	Run #	Analyzed Conc (mg/mL)	Percent of Target (%)	Mean Conc (mg/mL)	SD	RSD (%)	Mean Conc % of Target (%)
0	51 - 1	247	-----Not Quantifiable-----					
	51 - 2	248	-----Not Quantifiable-----					
5	51 - 3	249	5.14	103	5.12	0.027	0.54	102
	51 - 4	250	5.10	102				
10	51 - 5	251	10.0	100	10.1	0.14	1.3	101
	51 - 6	252	10.2	102				
20	51 - 7	253	20.8	104	20.7	0.12	0.56	103
	51 - 8	254	20.6	103				
30	51 - 9	255	31.2	104	30.9	0.54	1.8	103
	51 - 10	256	30.5	102				

865004 results.xls 2C-Gavage
Printed: 06Nov2016 2:30 PM

Table 9

At Least 10-Day Room Temperature Stability Assessment of the 26 Sep 2016 Diet Formulations

(Analyzed 10 Oct 2016)

Dose Conc (ppm)	Ref # (865004 -)	Run #	Analyzed Conc (ppm)	Percent of Target (%)	Mean Conc (ppm)	SD	RSD (%)	Mean Conc % of Target (%)
150	74 - 1	58	148	98.4	147	0.78	0.53	98.1
	74 - 1a	63	147	97.7				
300	74 - 2	59	249	83.0	247	2.3	0.9	82.5
	74 - 2a	64	246	82.0				
1250	74 - 3	60	887	71.0	889	2.6	0.29	71.1
	74 - 3a	65	891	71.3				
2500	74 - 4	61	1692	67.7	1702	14	0.84	68.1
	74 - 4a	66	1712	68.5				

865004 results.xls STB-Diet (2)
Printed: 06Nov2016 3:01 PM

Table 10

16-Day (7-Day Refrigerated and 9-Day Room Temperature) Stability Assessment of 26 Sep 2016 Diet Formulations

(Analyzed 12 Oct 2016)

Dose Conc (ppm)	Ref # (865004 -)	Run #	Analyzed Conc (ppm)	Percent of Target (%)	Mean Conc (ppm)	SD	RSD (%)	Mean Conc % of Target (%)
150	83 - 1	139	129	86.1	144	21	14	95.9
	83 - 2	140	159	106				
300	83 - 3	141	270	90.1	269	1.9	0.71	89.6
	83 - 4	142	268	89.2				
1250	83 - 5	143	1011	80.8	1007	4.8	0.47	80.6
	83 - 6	144	1004	80.3				
2500	83 - 7	145	1982	79.3	1977	7.3	0.37	79.1
	83 - 8	146	1972	78.9				

865004 results.xls STB-Diet (3)
Printed: 06Nov2016 3:01 PM

Table 11
Concentration Assessment of the 06 Sep 2016 Gavage Formulations
(Analyzed 07 Sep 2016)

Dose Conc (mg/mL)	Ref # (865004 -)	Run #	Analyzed Conc (mg/mL)	Percent of Target (%)	Mean Conc (mg/mL)	SD	RSD (%)	Mean Conc % of Target (%)
5	13 - 1	40	5.15	103	5.14	0.022	0.43	103
	13 - 2	41	5.12	102				
10	13 - 3	42	9.80	98.0	9.80	0.0066	0.067	98.0
	13 - 4	43	9.79	97.9				
20	13 - 5	44	19.2	95.8	19.2	0.016	0.083	95.8
	13 - 6	45	19.2	95.9				
30	13 - 7	46	28.9	96.2	29.3	0.60	2.1	97.6
	13 - 8	47	29.7	99.0				

865004 results.xls 1C-Gavage
Printed: 06Nov2016 3:01 PM

Table 12

At Least 3-Day Refrigerated Storage Stability Assessment of the 06 Sep 2016 Gavage Formulations

(Analyzed 09 Sep 2016)

Conc. (mg/mL)	Ref # (865004 -)	Run #	Analyzed Conc. (mg/mL)	Percent of Target (%)	Mean Conc. (mg/mL)	SD	RSD (%)	Mean Conc % of Target (%)
5	29 - 1	35	5.33	107	5.33	0.0027	0.050	107
	29 - 2	36	5.33	107				
10	29 - 3	37	10.5	105	10.5	0.047	0.45	105
	29 - 4	38	10.4	104				
20	29 - 5	39	21.5	107	21.5	0.0065	0.030	107
	29 - 6	40	21.5	107				
30	29 - 7	41	32.1	107	32.2	0.15	0.47	107
	29 - 8	42	32.3	108				

865004 results.xls 3D R Stb-Gavage
Printed: 06Nov2016 3:01 PM

APPENDIX 1

Test Material Information

SIGMA-ALDRICH®

sigma-aldrich.com

3050 Spruce Street, Saint Louis, MO 63103, USA
Website: www.sigmaaldrich.com
Email USA: techserv@sial.com
Outside USA: eurtechserv@sial.com

Certificate of Analysis

Product Name:
4(5)-Methylimidazole – 98%

**Product Number:**	**199885**
**Batch Number:**	**MKBV5083V**
Brand:	ALDRICH
CAS Number:	822-36-6
MDL Number:	MFCD00005201
Formula:	C4H6N2
Formula Weight:	82.10 g/mol
Quality Release Date:	30 APR 2015

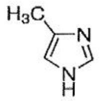

Test	Specification	Result
Appearance (Color)	White to Yellow	White
Appearance (Form)<br>Powder, Crystals, Crystalline Powder, Solid and/or Chunks	Conforms to Requirements	Solid
Infrared Spectrum	Conforms to Structure	Conforms
Titration by HCL	97.5 - 102.5 %	99.5 %
Purity (GC)	$\geq$ 97.5 %	99.9 %
Solubility (Turbidity)<br>5% in H2O	Clear	Clear
Solubility (Color)	Colorless to Yellow	Colorless

Ali Ataei, Manager
Quality Control
Milwaukee, WI US

Sigma-Aldrich warrants, that at the time of the quality release or subsequent retest date this product conformed to the information contained in this publication. The current Specification sheet may be available at Sigma-Aldrich.com. For further inquiries, please contact Technical Service. Purchaser must determine the suitability of the product for its particular use. See reverse side of invoice or packing slip for additional terms and conditions of sale.

APPENDIX 2

Laboratory Methods

Analytical Method for the Analysis of 4-methylimidazole in Rodent Diet by HPLC/UV

Approved by:

Date: 09 NOV 2016

Archana M. Akalkotkar, PhD
Research Chemist, Analytical Chemistry

Prepared by:

Date: 09Nov2016

Tara E. Sansom, BS
Chemist II, Analytical Chemistry

Charles River Analytical Chemistry Department

TABLE OF CONTENTS

1. PURPOSE....3
2. SCOPE....3
3. DEFINITIONS/ABBREVIATIONS....3
4. EQUIPMENT AND SUPPLIES....4
5. PROCEDURE....5
5.1. Preparation of Reagents....5
5.1.1. Preparation of Diluent: 4% Phosphoric Acid in MeOH....5
5.1.2. Mobile Phase: 0.005 M Sodium Dodecyl Sulfate 0.03 M Sodium Phosphate, Dibasic, Dihydrate in 32.5% MeOH....5
5.2. Preparation of Stock Solutions....5
5.2.1. Preparation of Calibration Stock Solution....6
5.2.2. Preparation of QC Stock Solution....6
5.3. Preparation of Calibration Standards....6
5.4. Preparation of QC Samples (Simulated Formulations)....6
5.5. Formulation Sample Collection and Processing....7
5.6. Instrumentation....7
5.6.1. HPLC Set-Up Parameters (Gradient)....7
5.7. Quantitation, Acceptance Criteria, and Data Reporting....8
6. VALIDATION HISTORY/STABILITY PARAMETERS/REFERENCES/SYNONYMS....8
7. REVISION HISTORY....8

1. PURPOSE

The purpose of this method is to describe procedures to be employed for the analysis of 4-methylimidazole (4-MEI) in rodent diet by HPLC/UV.

2. SCOPE

The procedures provided in this method are applicable for the quantitation of 4-MEI in rodent diet at concentrations ranging from 125 to 2500 ppm 4-MEI.

3. DEFINITIONS/ABBREVIATIONS

The following abbreviations may appear in this method:

4-MEI - 4-methylimidazole
μL - microliter
μg - microgram
ACN - acetonitrile
cm - centimeter
CAD - charged aerosol detector
CMC - carboxymethylcellulose
DAD - diode array detector
DI - deionized
DMSO - dimethylsulfoxide
ECD - electron capture detector
EtOH - ethanol
FA - formic acid
FID - flame ionization detector
GAA - glacial acetic acid
GC - gas chromatography
HPLC - high performance liquid chromatography
HPMC - hydroxypropyl methylcellulose
IC - ion chromatography
IS - internal standard
kg - kilogram
L - liter
M - molar

MC	-	methylcellulose
MeOH	-	methanol
mg	-	milligram
mL	-	milliliter
mm	-	millimeter
mM	-	millimolar
MS	-	mass spectrometry
NA	-	not applicable
ng	-	nanogram
nm	-	nanometer
ppm	-	parts per million
pg	-	picogram
QC	-	quality control
%RE	-	percent relative error
RI	-	refractive index detector
RSD	-	relative standard deviation
SD	-	standard deviation
TFA	-	trifluoroacetic acid
UHPLC	-	ultra-high performance liquid chromatography
UV	-	ultraviolet
v	-	volume
VIS	-	visible
VWD	-	variable wavelength detector
w	-	weight

4. EQUIPMENT AND SUPPLIES

The following equipment and/or supplies may be used while performing this method:

96-well analytical plates
Analytical balances and weighing vessels
Autosampler vials and caps with appropriate liners
Class A glass pipettes
Corning® Costar® 3635, acrylic 96-well UV plates

Disposable pipettes
Laboratory glassware (*e.g.*, volumetric flasks, graduated cylinders, beakers, *etc.*)
Laboratory refrigerators, freezers, incubators, *etc.*
Laboratory sample mixing equipment (shakers, vortexers, *etc.*)
Membrane filters of 0.45-µm (or finer) porosity
pH meters
Polypropylene labware (*e.g.*, volumetric flasks, graduated cylinders, beakers, *etc.*)
Polypropylene tubes and caps with appropriate liners
Repeater pipettes with appropriately sized tips
Sonicators
Syringe-end filters of 0.45-µm (or finer) porosity
Syringes with dosing cannula or needles

5. PROCEDURE

5.1. Preparation of Reagents

Volumes of these reagents can be adjusted as long as proportionality is maintained and their preparation is documented in the study records. Expiration dates and storage conditions of prepared reagents will be assigned according to SOPs.

5.1.1. Preparation of Diluent: 4% Phosphoric Acid in MeOH

Transfer 1900 mL of MeOH to a 2-L volumetric flask. Using a graduated cylinder, add 80 mL of phosphoric acid to the flask. Mix the contents of the flask by swirling it gently. Bring the contents in the flask to volume with MeOH and stir to mix.

5.1.2. Mobile Phase: 0.005 M Sodium Dodecyl Sulfate 0.03 M Sodium Phosphate, Dibasic, Dihydrate in 32.5% MeOH

Dissolve approximately 1.4 g of sodium dodecyl sulfate and approximately 5.9 g of sodium phosphate, dibasic, dihydrate in a 1000-mL flask with approximately 500 mL of DI water. Add 325 mL of MeOH to the flask and stir to mix. Adjust to final volume with DI water and mix. Filter the solution as needed.

5.2. Preparation of Stock Solutions

The following preparation schemes are suggested approaches. Appropriate modifications to reach the targeted nominal calibration and QC concentrations are acceptable. For example, if the concentration of a primary stock solution is not practical for use in the preparation of calibration

standards or QC samples, a secondary stock solution may be prepared. The preparation of any secondary or working stock solutions will be documented in the study records. Volumes of these stock solutions can be adjusted as long as proportionality is maintained and the preparation is documented in the study records. Expiration dates and storage conditions of stock solutions are assigned based on available stability data.

Stock solutions are corrected for purity, water content, and salt content, if applicable.

5.2.1. Preparation of Calibration Stock Solution

A 4-MEI calibration stock solution is prepared at a concentration of 5.00 mg 4-MEI/mL as follows. Weigh approximately 50.0 mg of 4-MEI (no correction for purity) in a tared glass weigh boat and transfer to a 10.0-mL volumetric flask with rinses of diluent. Mix and/or sonicate the preparation as necessary to achieve complete dissolution. Add additional diluent to yield the desired concentration, and thoroughly mix the solution.

5.2.2. Preparation of QC Stock Solution

A 4-MEI QC stock solution is prepared at a concentration of 5.00 mg 4-MEI/mL as follows. Weigh approximately 50.0 g of 4-MEI (no correction for purity) in a tared glass weigh boat and transfer to a 10-mL volumetric flask with rinses of diluent. Mix and/or sonicate the preparation as necessary to achieve complete dissolution. Add additional diluent to yield the desired concentration, and thoroughly mix the solution.

5.3. Preparation of Calibration Standards

Prepare calibration standards at concentrations ranging from 62.5 and 2500 ppm 4-MEI/mL by combining aliquots of the calibration stock solution, matrix (diet), and diluent. The samples were mixed with vortex action and shaken for at least 30 minutes at a high speed on a flatbed shaker. Optionally, the samples may be sonicated and probe sonicated as needed. The samples will be centrifuged at 1200 rpm for at least 2 minutes. Prepare at least triplicate calibration standards at each concentration for any validation sessions; prepare at least single calibration standards at each concentration for routine analyses.

5.4. Preparation of QC Samples (Simulated Formulations)

Prepare QC samples to simulate the processing of formulations at concentrations of 125 to 2500 ppm 4-MEI/mL in diet (nominal QC concentrations) by combining aliquots of the appropriate QC stock solution, matrix (diet), and diluent in polypropylene tubes. The samples were shaken on a horizontal flat-bed shaker for at least 30 minutes. Optionally, the samples may be sonicated and probe sonicated as needed. The samples will be centrifuged at 1200 rpm for at least 2 minutes. Further dilute the QC samples as needed with diluent to achieve a final diluted concentration within the calibration range. Prepare the QC samples in triplicate at each

concentration; prepare a single vehicle blank sample. Alternate QC concentrations may be prepared within the validated range and using the same general dilution schemes.

5.5. Formulation Sample Collection and Processing

At least duplicate dietary formulation samples if possible are processed by adding diluent and shaking at a high speed on a horizontal flatbed shaker for at least 30 minutes. Optionally, the samples may be sonicated and probe sonicated as needed. Centrifuge the samples at 1200 rpm for approximately 2 minutes. Portions of the processed samples will be transferred directly into amber autosampler vials. The remaining samples (back-up samples) are stored room temperature and, if not needed for analysis, will be discarded as indicated in the protocol or according to SOP if not designated in the protocol

5.6. Instrumentation

Instrumentation can be substituted provided that the design parameters of the substituted instrumentation are at least comparable to those of the unit initially used.

5.6.1. HPLC Set-Up Parameters (Gradient)

Instrument:	Agilent 1100 liquid chromatograph equipped with a variable wavelength detector, autosampler, and Dionex Chromeleon® software version 6.8, or equivalent system
Column:	Zorbax RX-C8, 5-µm particle-size, 150 × 4.6 mm
Mobile Phase:	0.005 M sodium dodecyl sulfate, 0.03M sodium phosphate, dibasic, dihydrate in 32.5% MeOH
Flow Rate:	1.00 mL/minute
Column Temperature:	40°C
Autosampler Temperature:	Ambient
Detector:	UV at 220 nm
Injection Volume:	10 µL
Retention Time:	Approximately 3.6 to 3.9 minutes for 4-MEI
Run Time:	10 minutes

5.7. Quantitation, Acceptance Criteria, and Data Reporting

Single injections are made of each calibration standard and processed QC and formulation sample. A calibration curve is constructed for each set of analyses. The 4-MEI peak areas (y) and the theoretical concentrations (x) of the calibration standards are fit with least-squares regression analysis to the quadratic function without weighting:

$$y = ax^2 + bx + c$$

Concentrations are calculated from the results of the regression analysis using Dionex Chromeleon® software (version 6.80). The concentration data are transferred to a Microsoft Excel® spreadsheet, where appropriate summary statistics, *i.e.*, mean, SD, RSD, %RE, and concentration as a percent of target concentration, are calculated and presented in tabular form. The concentrations of QC and/or formulation samples are calculated by applying any necessary factors to correct for sample dilution or unit conversion.

The analytical results are evaluated against acceptance criteria detailed in the protocol and/or SOPs AC-047 or AC-048. Deviations from expected results are investigated according to the appropriate departmental SOP.

Data is reported to the Principal Chemist, the Study Director, and other appropriate individuals.

6. VALIDATION HISTORY/STABILITY PARAMETERS/REFERENCES/SYNONYMS

The method was validated in WIL-865001 and extension validation was carried out in WIL-865003

7. REVISION HISTORY

Version 1 (Issued 12 Nov 2015) to Version 2
- Section 2, 5.3, and 5.4: Updated concentration range
- Section 5.5: Updated storage of back-up samples
- Section 5.6.1: Updated column and retention time

Analytical Method for the
Analysis of 4-methylimidazole in Sterile Water by HPLC/UV

Approved by:

Archana Akalkotkar Date: 09 NOV 2016

Archana M. Akalkotkar, PhD
Research Chemist, Analytical Chemistry

Prepared by:

Tara Sansom Date: 09Nov2016

Tara E. Sansom, BS
Chemist II, Analytical Chemistry

Charles River Analytical Chemistry Department

TABLE OF CONTENTS

1. PURPOSE....3
2. SCOPE....3
3. DEFINITIONS/ABBREVIATIONS....3
4. EQUIPMENT AND SUPPLIES....4
5. PROCEDURE....5
5.1. Preparation of Reagents....5
5.1.1. Preparation of Diluent: 4% Phosphoric Acid in MeOH....5
5.1.2. Preparation of Mobile Phase: 0.005 M Sodium Dodecyl Sulfate, 0.03M Sodium Phosphate, Dibasic, Dihydrate in 32.5% MeOH....5
5.2. Preparation of Stock Solutions....5
5.2.1. Preparation of Calibration Stock Solution....6
5.2.2. Preparation of QC Stock Solution....6
5.3. Preparation of Calibration Standards....6
5.4. Preparation of QC Samples in Vehicle (Spiked Dilution)....6
5.5. Formulation Sample Processing....6
5.6. Instrumentation....7
5.6.1. HPLC Set-Up Parameters (Gradient)....7
5.7. Quantitation, Acceptance Criteria, and Data Reporting....8
6. VALIDATION HISTORY/STABILITY PARAMETERS/REFERENCES/SYNONYMS....8
7. REVISION HISTORY....8

1. PURPOSE

The purpose of this method is to describe procedures to be employed for the analysis of 4-Methylimidazole (4-MEI) in sterile water by HPLC/UV.

2. SCOPE

The procedures provided in this method are applicable for the quantitation of 4-MEI in sterile water at concentrations ranging from 5.00 to 30.0 mg 4-MEI/mL.

3. DEFINITIONS/ABBREVIATIONS

The following abbreviations may appear in this method:

4-MEI - 4-methylimidazole
µL - microliter
µg - microgram
ACN - acetonitrile
cm - centimeter
CAD - charged aerosol detector
CMC - carboxymethylcellulose
DAD - diode array detector
DI - deionized
DMSO - dimethylsulfoxide
ECD - electron capture detector
EtOH - ethanol
FA - formic acid
FID - flame ionization detector
GAA - glacial acetic acid
GC - gas chromatography
HPLC - high performance liquid chromatography
HPMC - hydroxypropyl methylcellulose
IC - ion chromatography
IS - internal standard
kg - kilogram
L - liter
M - molar

MC - methylcellulose
MeOH - methanol
mg - milligram
mL - milliliter
mm - millimeter
mM - millimolar
MS - mass spectrometry
NA - not applicable
ng - nanogram
nm - nanometer
ppm - parts per million
pg - picogram
QC - quality control
%RE - percent relative error
RI - refractive index detector
RSD - relative standard deviation
SD - standard deviation
TFA - trifluoroacetic acid
UHPLC - ultra-high performance liquid chromatography
UV - ultraviolet
v - volume
VIS - visible
VWD - variable wavelength detector
w - weight

4. EQUIPMENT AND SUPPLIES

The following equipment and/or supplies may be used while performing this method:

96-well analytical plates
Analytical balances and weighing vessels
Autosampler vials and caps with appropriate liners
Class A glass pipettes
Corning® Costar® 3635, acrylic 96-well UV plates

Disposable pipettes
Laboratory glassware (*e.g.*, volumetric flasks, graduated cylinders, beakers, *etc.*)
Laboratory refrigerators, freezers, incubators, *etc.*
Laboratory sample mixing equipment (shakers, vortexers, *etc.*)
Membrane filters of 0.45-µm (or finer) porosity
pH meters
Polypropylene labware (*e.g.*, volumetric flasks, graduated cylinders, beakers, *etc.*)
Polypropylene tubes and caps with appropriate liners
Repeater pipettes with appropriately sized tips
Sonicators
Syringe-end filters of 0.45-µm (or finer) porosity
Syringes with dosing cannula or needles

5. PROCEDURE

5.1. Preparation of Reagents

Volumes of these reagents can be adjusted as long as proportionality is maintained and their preparation is documented in the study records. Expiration dates and storage conditions of prepared reagents will be assigned according to SOPs.

5.1.1. Preparation of Diluent: 4% Phosphoric Acid in MeOH

Transfer 1900 mL of MeOH to a 2-L volumetric flask. Using a graduated cylinder, add 80 mL of phosphoric acid to the flask. Mix the contents in the flask by swirling it gently. Bring the contents in the flask to volume with MeOH and stir to mix.

5.1.2. Preparation of Mobile Phase: 0.005 M Sodium Dodecyl Sulfate, 0.03M Sodium Phosphate, Dibasic, Dihydrate in 32.5% MeOH

Dissolve approximately 1.4 g of sodium dodecyl sulfate and approximately 5.9 g of sodium phosphate, dibasic, dihydrate in a 1000-mL flask with approximately 500 mL of DI water. Add 325 mL of MeOH to the flask and stir to mix. Bring up to the volume with DI water.

5.2. Preparation of Stock Solutions

The following preparation schemes are suggested approaches. Appropriate modifications to reach the targeted nominal calibration and QC concentrations are acceptable. For example, if the concentration of a primary stock solution is not practical for use in the preparation of calibration standards or QC samples, a secondary stock solution may be prepared. The preparation of any

secondary or working stock solutions will be documented in the study records. Volumes of these stock solutions can be adjusted as long as proportionality is maintained and the preparation is documented in the study records. Expiration dates and storage conditions of stock solutions are assigned based on available stability data.

Stock solutions are corrected for purity, water content, and salt content, if applicable.

5.2.1. Preparation of Calibration Stock Solution

A 4-MEI stock solution is prepared at a concentration of 0.100 mg 4-MEI/mL as follows. Weigh approximately 10.0 mg of 4-MEI in a tared glass weigh funnel and transfer to a 100-mL volumetric flask with rinses of ACN. Mix the preparation as necessary to achieve complete dissolution. Add additional ACN to yield the desired concentration, and thoroughly mix the solution.

5.2.2. Preparation of QC Stock Solution

A 4-MEI QC stock solution is prepared at a concentration of 5.00 mg 4-MEI/mL as follows. Weigh approximately 50.0 mg of 4-MEI in a tared glass weigh funnel and transfer to a 10-mL volumetric flask with rinses of ACN. Mix and/or sonicate the preparation as necessary to achieve complete dissolution. Add additional ACN to yield the desired concentration, and thoroughly mix the solution.

5.3. Preparation of Calibration Standards

Prepare calibration standards at concentrations ranging between 10.0 to 100 μg 4-MEI/mL by combining aliquots of the Calibration stock solution and diluent in polypropylene tubes. Mix the samples with vortex action. Prepare at least triplicate calibration standards at each concentration for any validation sessions; prepare at least single calibration standards at each concentration for routine analyses. Store the calibration stock solution and standards under appropriate conditions.

5.4. Preparation of QC Samples in Vehicle (Spiked Dilution)

Prepare Quality control samples to simulate the processing of formulations at concentrations of 5.00, 25.0 and 30.0 mg 4-MEI/mL by combining aliquots of the QC stock solution, sterile water, and diluent in polypropylene tubes. Mix the samples with vortex action. Further dilute the QC samples as needed with diluent to achieve a final diluted concentration within the calibration range. The QC samples will be prepared in triplicate at each concentration; a single matrix blank sample was prepared.

5.5. Formulation Sample Processing

Receive samples from the outside laboratory. Process at least 2 samples from each formulation for analysis; the remaining samples (back-up samples) are stored refrigerated (2°C to 8°C) and, if

not needed for analysis, will be discarded as indicated in the protocol or according to SOP if not designated in the protocol. Process samples by adding (diluent) as needed and mix with vortex action. Any remaining samples are stored at room temperature and, if not needed for analysis, will be discarded as indicated in the protocol or according to SOP if not designated in the protocol. Further dilute portions of the processed samples as needed with (diluent) to achieve a final diluted sample concentration within the calibration range. Store processed formulation samples under appropriate conditions.

5.6. Instrumentation

Instrumentation can be substituted provided that the design parameters of the substituted instrumentation are at least comparable to those of the unit initially used.

5.6.1. HPLC Set-Up Parameters (Gradient)

Instrument:	Agilent 1100 liquid chromatograph equipped with a variable wavelength detector, autosampler, and Dionex Chromeleon® software version 6.8, or equivalent system
Column:	Nucleosil C8, 5-µm particle size, 150 × 4.6 mm, or Zorbax RX-C8, 5-µm particle size, 150 × 4.6 mm
Mobile Phase:	0.005 M Sodium dodecyl sulfate, 0.03M sodium phosphate, dibasic, dihydrate in 32.5% MeOH
Flow Rate:	1.00 mL/minute
Column Temperature:	40°C
Autosampler Temperature:	Ambient
Detector:	UV at 220 nm
Injection Volume:	10 µL
Retention Time:	Approximately 4.1 to 4.4 minutes for 4-MEI
Run Time:	10 minutes

5.7. Quantitation, Acceptance Criteria, and Data Reporting

Single injections are made of each calibration standard and processed QC and formulation sample. A calibration curve is constructed for each set of analyses. The 4-MEI peak areas (y) and the theoretical concentrations (x) of the calibration standards are fit with least-squares regression analysis to the quadratic function without weighting.

$$y = ax^2 + bx + c$$

Concentrations are calculated from the results of the regression analysis using Dionex Chromeleon® software (version 6.80). The concentration data are transferred to a Microsoft Excel® spreadsheet, where appropriate summary statistics, *i.e.*, mean, SD, RSD, %RE, and concentration as a percent of target concentration, are calculated and presented in tabular form. The concentrations of QC and/or formulation samples are calculated by applying any necessary factors to correct for sample dilution or unit conversion.

The analytical results are evaluated against acceptance criteria detailed in the protocol and/or SOPs AC-047 or AC-048. Deviations from expected results are investigated according to the appropriate departmental SOP.

Data is reported to the Principal Chemist, the Study Director, and other appropriate individuals.

6. VALIDATION HISTORY/STABILITY PARAMETERS/REFERENCES/SYNONYMS

The method was validated in WIL-861001 and extension validation was carried out in WIL-861003.

7. REVISION HISTORY

NA

Appendix VI: *p* Value Table

Day 2

4-MEI Exposure Level (mg/kg/day)	Sex	Final Body Weight	Body Weight Gain	Sex	Final Body Weight	Body Weight Gain
0	M	-	-	F	-	-
50		0.8917	0.9982		0.9184	0.3956
100		1.0000	0.7045		0.6837	0.9997
200		0.4978	0.3476		0.0703	0.0573
300		0.7349	0.2646		0.1615	0.0529
Trend		0.9884	0.3149		0.3041	0.8889

Day 5

4-MEI Exposure Level (ppm)	Sex	Final Body Weight	Body Weight Gain	Food Consumption (g/kg bw/day)	Sex	Final Body Weight	Body Weight Gain	Food Consumption (g/kg bw/day)
0	M	-	-	-	F	-	-	-
150		0.9797	0.8510	1.0000		0.9334	0.9663	0.4644
300		1.0000	0.6871	0.9918		1.0000	1.0000	0.9221
1250		0.9923	0.9956	0.9606		0.9960	0.3997	0.3609
2500		0.9923	0.9817	0.5775		0.9900	0.2456	0.9188
Trend		0.9482	0.3064	0.7420		0.9407	0.9701	0.5332

Day 28

4-MEI Exposure Level (ppm)	Sex	Final Body Weight	Body Weight Gain	Food Consumption (g/kg bw/day)	Sex	Final Body Weight	Body Weight Gain	Food Consumption (g/kg bw/day)
0	M	-	-	-	F	-	-	-
150		0.7669	0.0192	0.9934		0.8578	0.9713	0.4445
300		0.5412	0.0055	0.9796		0.9649	0.5496	0.9983
1250		0.6203	0.2156	0.7223		0.8060	0.4612	0.0586
2500		0.8607	0.9996	0.9845		0.6253	0.9713	0.0306
Trend		0.2198	0.0015	0.6733		0.6230	0.2244	0.8259

Appendix VII: Study Protocol and Amendments

ILS

Study Title
Short Term Toxicity and Genomics Study of 4-Methylimidazole in B6C3F1 Male and Female Mice

ILS Project-Study Number
50074.0001

Performing Laboratory
Integrated Laboratory Systems, Inc.
601 Keystone Park Drive, Suite 200
Morrisville, NC 27560 USA

Sponsor
Scitovation, LLC
6 Davis Drive
RTP, NC 27709

Study Protocol Approval

Chris Bartlett
Sponsor Representative
ScitoVation, LLC

9/1/16
Date

Michael A. Streicker, B.S., LATG
Study Director
Integrated Laboratory Systems, Inc.

02 Sept 2016
Date

Reviewed by:

Cheryl Hobbs, Ph.D.
Director of Toxicology
Integrated Laboratory Systems, Inc.

01 Sept 2016
Date

Leslie Recio, Ph.D., DABT
Vice President, Research and Development
Integrated Laboratory Systems, Inc.

02 Sept 2016
Date

TABLE OF CONTENTS

INTRODUCTION 4
1.1 Background 4
1.2 Purpose 4
1.3 Regulatory Compliance 5
1.4 Sponsor 5
1.5 Testing Facility 5
1.6 Proposed Study Dates 5
TEST ARTICLE, CONTROL ARTICLE, AND VEHICLE(S) 6
2.1 Test Article: 4-Methylimidazole 6
2.2 Vehicle (Feed): NTP 2000 6
2.3 Vehicle (Oral Gavage): Sterile Water 7
2.4 Archival Samples 7
2.5 Dose Formulation Analysis 7
EXPERIMENTAL DESIGN 8
3.1 Test System 8
3.2 Animal Husbandry 9
3.3 Allocation 10
3.4 Group Designation 10
Table 1. Group Designation, Animal Identification, and Dose Levels 10
3.5 Dose Administration 11
3.5.1 Justification of Route of Administration 11
3.5.2 Justification of Dose Levels 11
3.5.3 Disposal of Dose Formulations 11
3.6 In-Life Animal Observations 11
3.7 Termination 12
3.8 Statistical Analysis 13
REPORT 13
RECORD RETENTION 13
REFERENCES 13
KEY PERSONNEL 14

INTRODUCTION

1.1 Background

4-Methylimidazole (4-MEI) is present in caramel colorings manufactured with ammonia catalysts and has potential human exposure in a wide variety of food and beverage products. 4-MEI was evaluated for short-term toxicity and long term carcinogenicity in F344 rats and B6C3F1 mice by the U.S. Department of Health and Human Services' National Toxicology Program. In these studies, groups of male and female rats and mice were fed diets containing 4-MEI for durations of 15 days, 14 weeks, or 2 years. Results of the 15-day study indicated a no-observed-adverse-effect-level (NOAEL) of 2500 ppm in rats and mice (highest dose tested). In the 14-week study, NOAELs of 1250 ppm (male rats), 5000 ppm (female rats), and 10,000 ppm (male and female mice; highest dose tested) were determined based primarily on the results of tissue histopathology. Alterations related to 4-MEI exposure included anemia, hepatocytic vacuolation, testicular degeneration (rats), prostatic atrophy (rats), and renal degeneration and dilation (mice).

For the 2-year study, 4-MEI concentrations in feed were 0, 625, 1250, or 2500 ppm (male rats), 0, 1250, 2500 or 5000 ppm (female rats), and 0, 312, 625, or 1250 ppm (male and female mice). Results indicated no evidence of carcinogenic activity in male rats, equivocal evidence of carcinogenicity (mononuclear cell leukemia) in female rats, and clear evidence of carcinogenicity (alveolar/bronchiolar adenoma or carcinoma) in male and female mice. Nonneoplastic effects in the 2-year study were observed in the liver of male and female rats (histiocytosis, chronic inflammation, focal fatty hepatoctye changes, and eosinophilic focus) and in the lungs of female mice (alveolar epithelium hyperplasia). 4-MEI was negative in genetic toxicology studies conducted by the NTP (Ames *Salmonella* test and rat or mouse micronucleus tests).

In this study, a short-term in life toxicity study with 4-MEI and concurrent measurement of genomic responses in target tissues will be conducted. The animal model will be the B6C3F1 mouse, since this species was susceptible to the formation of lung tumors following lifetime exposure of 4-MEI. The target tissues will be the lung and liver. The study will assess dose-response and temporal relationships. Doses of 4-MEI will be selected based on effects observed in previous toxicity studies with 4-MEI. Altered gene expression will be compared to tissue cell proliferation and histopathology. The pattern and dose response of observed alterations will provide signatures for 4-MEI effects. These signatures should identify early events occurring in target tissues that would be of value for understanding the mode-of-action and dose response of 4-MEI. The information gained from genomic screening could then be used for designing more specific *in vivo* and/or *in vitro* 4-MEI studies targeted to the understanding of induction of mouse lung tumors and relevance of effects to humans.

1.2 Purpose

The purpose of this study is to assess dose-response and time-dependent tissue and cellular responses following 4-MEI exposure. The collection of data on both gene expression and

toxicological endpoints (histopathology and cell proliferation), will allow characterization of the relationship of gene expression signatures with pathway alterations in tissues associated with 4-MEI exposure.

1.3 Regulatory Compliance

This study is designed as a research study and therefore not intended for submittal to any national or international regulatory agency. This study will be conducted to the highest standards of research practice including ILS Quality Control but it will not be audited by a Quality Assurance Unit.

1.4 Sponsor

ScitoVation, LLC
6 Davis Drive
RTP, NC 27709

Sponsor Representative

Chris Bartlett
Telephone No.: (919) 558-1400
E-mail: cbartlett@scitovation.com

1.5 Testing Facility

Integrated Laboratory Systems, Inc. (ILS)

Shipping Address:	601 Keystone Park Drive, Suite 200<br>Morrisville, NC 27560 USA
Mailing Address:	P.O. Box 13501<br>Research Triangle Park, NC 27709 USA

Study Director

Michael A. Streicker, B.S., LATG
Telephone No.: (919) 281-1110 ext. 721
Facsimile No.: (919) 281-1118
E-mail: mstreicker@ils-inc.com

1.6 Proposed Study Dates

Animal Arrival Date:	13 September 2016
Experimental Start Date:	29 September 2016
Experimental In-Life Termination Date:	27 October 2016

TEST ARTICLE, CONTROL ARTICLE, AND VEHICLE(S)

**2.1**	**Test Article:**	**4-Methylimidazole**
	CAS No.:	822-36-6
	Source:	Sigma Aldrich
	Lot/Batch No.:	MKBV5083V
	ILS Repository No.:	16-135
	Description:	Pale Yellow Crystal
	Purity:	98%
	Expiration Date:	27 July 2017
	Dose Formulation:	Diets containing 4-MEI will be prepared at ILS using a Patterson-Kelley twin-shell blender according to ILS SOP's in NTP 2000 feed at dose concentrations of 0, 150, 300, 1250, and 2500 ppm. The feed for the 28-day dose groups will be prepared every two weeks.<br><br>4-MEI will be prepared once at ILS in sterile USP water at dose concentrations of 0, 5, 10, 20, and 30 mg/mL and aliquoted into vials to be used daily during the study.
	Storage:	
	Test Article:	Room temperature and protected from light
	Dose Formulation:	Between 1 and 10°C (~4°C) and protected from light until placed in feed jars at room temperature.
	Stability:	
	Dose Formulation:	4-MEI in NTP 2000 feed has been shown to be stable for up to 36 days stored between 1 and 10°C.
**2.2**	**Vehicle (Feed):**	**NTP 2000**
	Source:	Zeigler (Gardners, PA)
	Lot/Batch No.:	01 July 2016

	Storage:	Room temperature
**2.3**	**Vehicle (Oral Gavage):**	**Sterile Water**
	Source:	Patterson Veterinary Supply
	Lot/Batch No.:	TBD
	ILS Repository No.:	TBD
	Description:	Clear liquid
	Storage:	Room temperature and protected from light

2.4 Archival Samples

Approximately 10 mg of 4-MEI, 1 mL of oral gavage dose formulations, and 10 mg of feed dose formulations will be stored between 0 and -30°C. After acceptance of the study report by the Sponsor, archival dose formulation samples will be discarded. Test and control articles will be maintained by ILS for five years following finalization of the study report. Transfer of archival samples may be requested by the Sponsor prior to the end of the five-year archival period. At the end of the five-year archival period, the Sponsor will be contacted for direction of appropriate disposition of archival samples remaining at ILS.

2.5 Dose Formulation Analysis

Test article dose formulations will be prepared at ILS and analyzed at Charles River (Ashland, OH). Test diet formulations will be prepared in NTP 2000 meal and approximately 10 gram samples will be collected from each dose level (including controls) at the time that the formulations are prepared. Oral (gavage) test material formulations will be prepared in water and approximately 5 mL samples from each dose level (including controls) will be collected from each dose level at the time that the formulations are prepared. A set of samples will be shipped to Charles River for stability analysis on ice packs. A portion of each diet sample will be tested upon arrival. A portion of each diet sample will be held at room temperature for 7 days, and then tested. The remaining portion of each diet sample will be refrigerated for 7 days starting on the day of arrival at CRL; after one week, it will be removed from the refrigerator and held at room temperature for 9 days and then tested. Stability analysis will be performed on feed samples at 1, 7, and 16 days post formulation and on oral gavage samples at 1 and 3 days post formulation. The oral gavage samples will kept at 0 and 10°C. The stability testing should be conducted prior to the start of the study. The feed samples and oral gavage samples should be stored by Charles River under the same conditions as the dose formulations used by ILS for the studies.

Immediately prior to initial dosing/exposure, an additional three sets of samples (10 grams for each dose level of test diet and 5 mL for each dose level of oral test material formulations) will

be collected and sent to Charles River for analysis of concentration acceptability. The three sets of test diet samples include: (1) one set at the initiation of the 5-day study, (2) one set at the initiation of the 28-day study, and (3) one set for the second batch of diet at the start of the third week of the 28-day study.

Analysis of the aqueous and diet formulations for concentration acceptability will be performed by Charles River using a qualified laboratory method (865.RDT.01, 865.WFI1.01). For the results to be considered acceptable, the analyzed concentration must be within 85% to 115% of the target concentration. Draft results will be transmitted to the Study Director and a summary report signed by the Principal Investigator will be provided for inclusion in the final study report.

Principal Investigator- (Dose Formula Analyses): Archana Akalkotkar,PhD.

WIL Research
1407 George Rd.
Ashland, OH 44805 USA

Records Retention
All data generated by Charles River will be archived by Charles River following WIL SOPs.

EXPERIMENTAL DESIGN

One hundred and twenty male and one hundred and twenty female B6C3F1 mice will be allocated to one of 15 designated dose groups. The animals in groups 1-5 will be administered one of five dose levels of 4-MEI or the vehicle control (water) for two consecutive days. The animals in groups 6-15 will be administered one of five dose levels of 4-MEI or the vehicle control (NTP 2000 feed) for five or twenty-eight consecutive days.

3.1 Test System

Species:	Mice, *Mus musculus*
Strain:	B6C3F1
Source:	Charles River Laboratories International, Inc. (Raleigh, NC)
Number/Sex:	120 males and 120 females
Acclimation:	At least fourteen days
Age at administration:	9-10 weeks of age
Weight at administration:	15-35 grams
Identification:	Each animal will be uniquely identified by ear punch prior to the start of the study. Until the animals are ear punched, they will be

	identified by the temporary numbers located on the animals' enclosures.
Justification:	This study is being conducted in the same animal model used in a 2-year bioassay to help elucidate the toxicity and genomic effects of the test substance and the relevance of this response to humans.

3.2 Animal Husbandry

All procedures are in compliance with the Animal Welfare Act Regulations, 9 CFR 1-4 and animals will be handled and treated according to the *Guide for the Care and Use of Laboratory Animals* (ILAR, 2011).

Housing:	One per cage
Cage Type:	Polycarbonate with micro-isolator tops
Cage Size:	17 cm wide by 28 cm long (476 cm^2 area) and 13 cm high
Bedding:	Absorbent heat-treated hardwood bedding (Northeastern Products Corp., Warrensburg, NY)
Cage Changes:	Once per week
Diet:	NTP 2000 (Zeigler, Gardner, PA) will be provided *ad libitum.*
Analysis:	The manufacturer's composition formula will be included in the raw data and reviewed prior to animal arrival.
Water:	Reverse osmosis (RO) treated tap water (City of Durham, NC) *ad libitum*
Supplied:	Polycarbonate water bottles with stainless steel sipper tubes
Analysis:	The results of the current annual comprehensive chemical analyses of RO water from National Testing Laboratories, Inc. (Cleveland, OH) will be reviewed prior to initiation of the study and will be included in the raw data.
Water Bottle Changes:	Once per week

Temperature: 20-25°C

Humidity: 30-70%

Lighting: 12/12 hour light/dark cycle

Enrichment: Nestlets (Ancare, Bellmont, NY)

Analysis: The manufacturer's analytical results of the nestlets will be included in the raw data and reviewed before study start.

3.3 Allocation

The animals will be assigned to a dose group using a procedure that stratifies animals across groups by body weight such that the mean body weight of each group is not statistically different from any other group using analysis of variance (ANOVA) (Statistical Analysis System version 9.2, SAS Institute, Cary, NC). Only clinically healthy animals will be used for allocation.

3.4 Group Designation

Table 1. Group Designation, Animal Identification, and Dose Levels

Group Number	Sex (M/F)	Animal Identification (M/F)		Dose	Test Article Dose Level	Dose Route	Day of Termination
1	8/8	001-008	009-016	Vehicle Control	0.0 mg/kg/day	Gavage	2
2	8/8	017-024	025-032	4-MEI	50 mg/kg/day	Gavage	2
3	8/8	033-040	041-048	4-MEI	100 mg/kg/day	Gavage	2
4	8/8	049-056	057-064	4-MEI	200 mg/kg/day	Gavage	2
5	8/8	065-072	073-080	4-MEI	300 mg/kg/day	Gavage	2
6	8/8	081-088	089-096	Vehicle Control	0 ppm	Feed	5
7	8/8	97-104	105-112	4-MEI	150 ppm	Feed	5
8	8/8	113-120	121-128	4-MEI	300 ppm	Feed	5
9	8/8	129-136	137-144	4-MEI	1250 ppm	Feed	5
10	8/8	145-152	153-160	4-MEI	2500 ppm	Feed	5
11	8/8	161-168	169-176	Vehicle Control	0 ppm	Feed	28
12	8/8	177-184	185-192	4-MEI	150 ppm	Feed	28

Group Number	Sex (M/F)	Animal Identification (M/F)		Dose	Test Article Dose Level	Dose Route	Day of Termination
13	8/8	193-200	201-208	4-MEI	300 ppm	Feed	28
14	8/8	209-216	217-224	4-MEI	1250 ppm	Feed	28
15	8/8	225-232	233-240	4-MEI	2500 ppm	Feed	28

3.5 Dose Administration

For groups 1-5, dose formulations will be administered via oral gavage at a dose volume of 10 mL/kg body weight for two consecutive days. Dose volume will be based on individual animal daily body weight. Dose formulations will be placed on a stir plate at least 30 minutes prior to dosing and continuously stirred. The second dose will occur 24 hours (± 30 minutes) from the previous dose. The dosing sequence will be stratified across dose groups; one animal from each group and then repeated until all animals are dosed.

For groups 6-10, dose formulations will be administered via feed *ad libitum* for 5 days. For groups 11-15, dose formulations will be administered via feed *ad libitum* for 28 days.

3.5.1 Justification of Route of Administration

An oral route of administration is consistent with the main route of human exposure.

3.5.2 Justification of Dose Levels

The dose levels were selected following the conduct of an NTP 2-year bioassay.

3.5.3 Disposal of Dose Formulations

Dose formulations will be disposed of as hazardous material.

3.6 In-Life Animal Observations

Mortality/Moribundity: Twice daily on weekdays, once daily on weekends/holidays

Clinical Observations: Observed within two days of arrival, for allocation of animals to a dose group, weekly, and at termination. Additional clinical observations may be recorded after approval by the Study Director. Animals in groups 1-5 will have an observation collected prior to dosing each day.

Body Weights: Collected within two days of arrival, for allocation of animals to a dose group, at study start, weekly, and at termination. Additional body weight measurements may be recorded after approval by the

Study Director. Animals in groups 1-5 will have a body weight collected prior to dosing each day.

Feed Consumption: For animals in groups 6-15, feed jars will be weighed on the day of body weight collection each week. Feed consumption (g/kg body weight/day) will be calculated from the start of exposure to the necropsy date for the 5 (5 day consumption) and 28 (weekly consumption for each week) day time points. Additional feed jar weights may be collected as feed is added to cages due to spillage or high consumption.

3.7 Termination

Moribunds/Unscheduled: Tissue collection will not be performed on accidental deaths, moribund, or animals found dead during the acclimation period.

Beginning on the first day of dose administration, any animals found moribund or dead will be necropsied, and cause of death will be determined and recorded, if possible. Moribund animals will be euthanized by carbon dioxide (CO_2) inhalation and death confirmed by cervical dislocation.

Scheduled: For groups 1-5, approximately 6 hours (± 30 minutes) after the final dose administration, animals will be euthanized by CO_2 asphyxiation and death confirmed by exsanguination.

For groups 6-10, on Day 5 animals will be euthanized by CO_2 asphyxiation and death confirmed by exsanguination.

For groups 11-15, on Day 28 animals will be euthanized by CO_2 asphyxiation and death confirmed by exsanguination.

Tissue Collection: For each animal, the right lung lobes will be perfused with RNAlater and prepared for gene expression evaluation and stored at or below -70°C for analysis.

The left lung lobes will be perfused with 10% neutral buffered formalin (NBF).

A section of the left lobe of the liver will be placed in RNAlater stored at or below -70°C for analysis. The remaining lobe fixed in 10% NBF for 18-24 hours and then transferred to 70% histology grade alcohol prior to paraffin embedding. The remaining lobes of the liver will be cubed, pieces frozen individually, and all frozen

pieces placed in a cryovial and flash frozen in liquid N_2 and placed on dry ice until stored at or below -70°C for analysis.

The kidneys (separately), testes (separately), ovaries (separately) and brain will be cubed and placed in RNAlater in cryovials and placed on dry ice until stored at or below -70°C for analysis.

Specific information on the genomic tissue analysis will be included as a separate procedure.

3.8 Statistical Analysis

Individual animal data and group mean and standard deviations will be calculated and reported.

For final body weight, body weight gain and feed consumption measurements, data will be analyzed using Statistical Analysis System version 9.2 (SAS Institute, Cary, NC). Homogeneity of variance will be analyzed using Levene's test, and normality of the vehicle control data will be assessed using a Shapiro-Wilk test. Homogenous data will then be analyzed using a one way analysis of variance (ANOVA) test and test article administered groups will be compared to the vehicle control group using Dunnett's multiple comparison test. Dose-dependent changes will be evaluated using a linear regression model.

Data that are not homogeneous and normally distributed will be transformed and re-assessed. In the event data cannot be transformed to be homogenous and normally distributed, the data will be analyzed using the non-parametric Dunn's test. Dose-dependent changes will be evaluated using an appropriate non-parametric trend test.

All statistical analysis of the genomics data will be conducted by ScitoVation.

REPORT

The draft report will include all items in the study protocol as well as a comprehensive presentation of all data collected in the study. The final report will be submitted as one hard copy and one electronic copy.

RECORD RETENTION

All original data (including the original signed study protocol and all amendments [if any], test article information, animal receipt records, animal caretaker records, body weight records, clinical observations, etc.) and the original final report will be maintained by ILS for five years following finalization of the study report. Transfer of study records may be requested by the Sponsor prior to the end of the five-year archival period. At the end of the five-year archival period, the Sponsor will be notified for direction of appropriate disposition of study records remaining at ILS.

REFERENCES

Institute of Laboratory Animal Resources. (2011). *Guide for the Care and Use of Laboratory Animals.* National Academy Press, Washington, DC.

KEY PERSONNEL

Study Director:	Michael Streicker, B.S., LATG
Vice President, Research and Development:	Leslie Recio, Ph.D., DABT
Director of Toxicology:	Cheryl Hobbs, Ph.D.
Investigative Toxicology Study Coordinator:	Eileen Phillips, B.S., LAT
Formulations Manager:	Carol Swartz, Ph.D., DVM
Necropsy Coordinator:	John Pope, B.S.
Histology Coordinator:	John Pope, B.S.
Attending Veterinarian:	Alyssa McIntyre, D.V.M., DACLAM
Health and Safety Manager:	Michael Streicker, B.S., LATG

Integrated Laboratory Systems, Inc.
Protocol Amendment

ILS Project No.-Study No.:	50074.0001
Sponsor Study No.:	NA
Protocol Amendment No.:	1

Section Amended:	2.1 Dose Formulation
Amendment Made:	Diets containing 4-MEI will be prepared at Charles River (CRL) (Ashland, OH) using a twin-shell blender according to CRL SOPs in NTP 2000 feed at dose concentrations of 0, 150, 300, 1250, and 2500 ppm. The feed for the 28-day dose groups will be prepared approximately every two weeks. Prepared diets will be shipped under ambient conditions to ILS. One received at ILS, feed will either be stored at ambient conditions for dose administration or stored refrigerated for subsequent feedings.<br><br>4-MEI will be prepared once at ILS in sterile USP water at dose concentrations of 0, 5, 10, 20, and 30 mg/mL and aliquoted into vials to be used daily during the study.
Reason for Amendment:	This changed is being made for the diet preparation to be performed at Charles River Laboratories.
Section Amended:	2.5 Dose Formulation Analysis
Amendment Made:	Test article dose formulations will be prepared at Charles River and analyzed at Charles River. Test diet formulations will be prepared in NTP 2000 meal and approximately 50 gram samples will be collected from each dose level (including controls) at the time that the formulations are prepared. Oral (gavage) test material formulations will be prepared in water and approximately 5 mL samples from each dose level (including controls) will be collected from each dose level at the time that the formulations are prepared. A portion of each diet sample will be tested upon

formulation. A portion of each diet sample will be held at room temperature for at least 10 days, and then tested to determine that stability of 4-MEI in the NTP 2000 diet. The remaining portion of each diet sample will be refrigerated for at least 7 days starting on the day of formulation at CRL; after at least 7 days, it will be removed from the refrigerator and held at room temperature for at least 9 days and then tested. Stability analysis will be performed on feed samples at least 1, 7, and 16 days post formulation in various storage conditions and on oral gavage samples at 1 and 3 days post formulation. The oral gavage samples will be kept at 0 and 10°C. The feed samples and oral gavage samples should be stored by Charles River under the same conditions as the dose formulations used by ILS for the studies.

Prior to initial dosing/exposure, an additional three sets of samples (10 grams for each dose level of test diet and 5 mL for each dose level of oral test material formulations) will be collected and sent to Charles River for analysis of concentration acceptability. The three sets of test diet samples include: (1) one set at the initiation of the 5-day study, (2) one set at the initiation of the 28-day study, and (3) one set for the second batch of diet at the start of the third week of the 28-day study.

Analysis of the aqueous and diet formulations for concentration acceptability will be performed by Charles River within 24 hours of receipt of samples using a qualified laboratory method (865.RDT.01, 865.WFI1.01). For the results to be considered acceptable, the analyzed concentration must be within 85% to 115% of the target concentration. Draft results will be transmitted to the Study Director and a summary report signed by the Principal Investigator will be provided for inclusion in the final study report.

Principal Investigator- (Dose Formula Analyses):
Archana Akalkotkar, PhD.

Charles River
1407 George Rd.
Ashland, OH 44805 USA

<u>Records Retention</u>
All data generated by Charles River will be archived by Charles River following Charles River SOPs.

Reason for Amendment: This changed is being made for the diet preparation to be performed at Charles River Laboratories.

Chris Bartlett
Chris Bartlett
Sponsor Representative
ScitoVation, LLC

9/21/16
Date

Michael A. Streicker, B.S., LATG
Study Director
Integrated Laboratory Systems, Inc.

22 Sept 2016
Date

Reviewed by:

Cheryl Hobbs
Cheryl Hobbs, Ph.D.
Director of Toxicology
Integrated Laboratory Systems, Inc.

22 Sept 2016
Date

Leslie Recio, Ph.D., DABT
Vice President, Research and Development
Integrated Laboratory Systems, Inc.

09/22/2016
Date

Integrated Laboratory Systems, Inc.
Protocol Amendment

ILS Project No.-Study No.:	50074.0001
Sponsor Study No.:	NA
Protocol Amendment No.:	2

Section Amended: 3.7 Tissue Collection

Amendment Made:

Blood (maximum) will be collected via cardiac puncture and collected in EDTA tubes (500 µl tubes). Tubes will be spun at 1500 g for 10 min at 4°C and the plasma removed and stored at or below -70°C for analysis.

For each animal, the right lung lobes harvested for subsequent gene expression analysis will be perfused with RNAlater (≥5 volumes), cubed, and stored at 2-8°C for 1-30 days and then stored at -15°C to -25°C indefinitely.

The left lung lobes will be perfused with 10% neutral buffered formalin (NBF).

A section of the left lobe of the liver will be cubed, fully immersed in RNAlater (≥5 volumes), stored at 2-8°C for 1-30 days and then stored at -15°C to -25°C indefinitely. The remaining lobe will be fixed in 10% NBF for 18-24 hours and then transferred to 70% histology grade alcohol prior to paraffin embedding. The remaining lobes of the liver will be cubed, pieces frozen individually, all frozen pieces placed in a cryovial and flash frozen in liquid N_2, and placed on dry ice until stored at or below -70°C for possible subsequent analysis.

The kidneys (separately), testes (separately), ovaries (separately) and brain will be cubed, fully immersed in RNAlater in cryovials and placed on wet ice until stored at 2-8°C for 1-30 days and then stored at -15°C to -25°C indefinitely.

Specific information on the genomic tissue analysis will be included

in Study Specific Procedure No. 1.

Reason for Amendment: This change is being made to clarify the procedures for collection and storage of blood and tissues.

Chris Bartlett
Sponsor Representative
ScitoVation, LLC

9/29/16
Date

Michael A. Streicker, B.S., LATG
Study Director
Integrated Laboratory Systems, Inc.

30 Sept 2016
Date

Reviewed by:

Cheryl Hobbs, Ph.D.
Director of Toxicology
Integrated Laboratory Systems, Inc.

30 Sept 2016
Date

Leslie Recio, Ph.D., DABT
Vice President, Research and Development
Integrated Laboratory Systems, Inc.

30 Sept 2016
Date
